# Yeast-based automated high-throughput screens to identify anti-parasitic lead compounds

**DOI:** 10.1098/rsob.120158

**Published:** 2013-02

**Authors:** Elizabeth Bilsland, Andrew Sparkes, Kevin Williams, Harry J. Moss, Michaela de Clare, Pınar Pir, Jem Rowland, Wayne Aubrey, Ron Pateman, Mike Young, Mark Carrington, Ross D. King, Stephen G. Oliver

**Affiliations:** 1 Cambridge Systems Biology Centre and Department of Biochemistry, University of Cambridge, Sanger Building, 80 Tennis Court Road, Cambridge CB2 1GA, UK; 2 Department of Computer Science, Aberystwyth University, Aberystwyth SY23 3DB, UK; 3 School of Chemical Engineering and Analytical Science, University of Manchester, Manchester M13 9PL, UK; 4 School of Computer Science, University of Manchester, Manchester M13 9PL, UK; 5 Institute of Biological, Environmental and Rural Sciences, Aberystwyth University, Aberystwyth SY23 3DD, UK

**Keywords:** drug screening, parasites, yeast, automation, tropical diseases

## Abstract

We have developed a robust, fully automated anti-parasitic drug-screening method that selects compounds specifically targeting parasite enzymes and not their host counterparts, thus allowing the early elimination of compounds with potential side effects. Our yeast system permits multiple parasite targets to be assayed in parallel owing to the strains’ expression of different fluorescent proteins. A strain expressing the human target is included in the multiplexed screen to exclude compounds that do not discriminate between host and parasite enzymes. This form of assay has the advantages of using known targets and not requiring the *in vitro* culture of parasites. We performed automated screens for inhibitors of parasite dihydrofolate reductases, *N*-myristoyltransferases and phosphoglycerate kinases, finding specific inhibitors of parasite targets. We found that our ‘hits’ have significant structural similarities to compounds with *in vitro* anti-parasitic activity, validating our screens and suggesting targets for hits identified in parasite-based assays. Finally, we demonstrate a 60 per cent success rate for our hit compounds in killing or severely inhibiting the growth of *Trypanosoma brucei*, the causative agent of African sleeping sickness.

## 2. Introduction

Parasitic diseases such as malaria, schistosomiasis, leishmaniasis, sleeping sickness and Chagas disease affect millions of people every year, leading to severe morbidity and death. For example, malaria caused by parasites of the genus *Plasmodium* kills over half a million people every year [1]. The disease is primarily treated by chloroquine, artemisinin and antifolates (e.g. pyrimethamine). However, *Plasmodium* spp. have become resistant to all of these drugs [2].

There is a pressing need for new treatments targeting these diseases, which have often been neglected because they overwhelmingly or exclusively affect the inhabitants of developing countries [3,4]. However, this is changing with the investment of funds from organizations such as the Gates Foundation, Medicines for Malaria Venture, the Drugs for Neglected Diseases initiative and the Institute for One World Health [5,6], and companies such as Novartis [7], GSK [8] and Pfizer. Various groups have developed efficient high-throughput drug-screening methods based on intact parasites [9–12]. These cell-based assays screen for compounds that inhibit or kill pathogens cultured *in vitro*. This provides assurance that the compound is active against the pathogen, but provides no information about its mechanism of action or general cytotoxicity. Moreover, for cell-based assays, the pathogen must be culturable. This requirement is particularly problematic when designing screens for anti-parasitic compounds because it may be extremely difficult or impossible to culture the parasite or one of its life cycle stages outside of an animal host. For instance, *Plasmodium vivax* (the major cause of malaria in South America and southeast Asia) cannot be continuously maintained *in vitro* [13]*,* and techniques for cultivating liver stages of plasmodia are still in their infancy, and do not generate sufficient parasites for high-throughput automated screens [14].

Conversely, the biochemical strategy involves the selection of a target protein whose activity is essential for the growth or survival of the pathogen. This approach has the advantage of selecting candidate compounds of known mechanism of action; these can be rationally improved, particularly if the target protein's structure has been determined. The biochemical strategy has the disadvantages that it provides no information about drug uptake into cells, whether the drug will kill the pathogen, or whether it will show general cytotoxicity and thus be likely to injure the host [15].

To address these issues, we have designed an anti-parasite assay based on genetically engineered yeast strains. Our method enables automated, high-throughput, live-cell, target-based screens to identify novel compounds that specifically inhibit the activity of proteins that have been suggested as targets for anti-parasite drugs. This represents a complementary approach to parasite-based methods, and is able to identify novel chemical scaffolds for further development as anti-parasitic drugs.

The yeast *Saccharomyces cerevisiae* has been successfully used as a host for the expression of heterologous proteins for over three decades. Yeast cells expressing parasite proteins can provide a well-characterized and exploitable platform for screens attempting to identify novel anti-parasitics. For example, dihydrofolate reductase (DHFR) is an anti-parasitic drug target that is present in organisms ranging from bacteria to humans. It is the target of pyrimethamine treatment of malaria and human tumours, because rapidly growing cells require folate to produce thymidine [16]. In yeast, *dfr1* mutations lead to loss of DHFR activity, and Sibley and co-workers [17–19] have achieved the complementation of such mutations by overexpression of human and *Plasmodium* DHFRs. They have also demonstrated the suitability of the mutant strains for drug screens in plate assays. Phosphoglycerate kinase (PGK) is a central enzyme in glycolysis and gluconeogenesis, and is essential for the blood stages of many parasites. However, the human enzyme is not expressed in erythrocytes, and so PGK has been proposed as a target for anti-parasitic drugs [20,21]. *N*-myristoyltransferase (NMT) is an enzyme responsible for the modification of proteins to enable their targeting to membranes [22–24]. NMTs are essential enzymes conserved from kinetoplastid parasites to humans and are successful drug targets [23,24].

We have engineered *S. cerevisiae* strains where genes encoding enzymes that are essential for yeast growth (DHFR, NMT or PGK) were deleted and their function complemented by the heterologous expression of the orthologous enzymes from either human or parasites. Yeast cultures, which can be grown rapidly and at low cost, are ideal for use in automated screens. Yeast cells are suitable hosts for the expression of enzymes essential for different life stages of parasites, some of which cannot be propagated *in vitro*, thus providing a platform for *in vivo* drug screens. Yeast cells can be refractory to drug treatments owing to a protective cell wall and the presence of multiple drug export pumps. The most pleiotropic drug export pump in *S. cerevisiae* is Pdr5p; therefore, we engineered all of our strains to lack this drug export protein and consequently sensitized them to a large range of chemical entities.

Here, we report the construction of a series of strains that are genetically identical apart from genes encoding different heterologous drug targets, and fluorescent proteins that allow the growth of multiple strains to be followed in a single culture. By these means, the drug sensitivity observed in a particular strain can be directly linked to the *in vivo* inhibition of the heterologous target. This approach also allows the early identification of compounds that exhibit general cytotoxicity, and identifies compounds that inhibit the activity of the target proteins from the parasites, but have no effect on the equivalent human protein.

In this paper, the drug targets DHFR, PGK and NMT from a range of human parasites are used as examples to demonstrate the utility of our assay. We have identified compounds that inhibit each of the target enzymes expressed in yeasts, but fail to inhibit the corresponding human enzyme. We performed Tanimoto chemical similarity searches between our *Plasmodium* hits and compounds with demonstrated anti-plasmodial activity *in vitro* [25–27], indirectly validating our anti-plasmodial hit compounds and suggesting intracellular targets for the compounds identified in parasite-based screens. Moreover, we have screened a number of our ‘hit’ compounds against *Trypanosoma brucei* grown in culture and shown that 60 per cent either kill or severely inhibit the growth of this parasite.

## 3. Material and methods

### 3.1. Strain and plasmid constructs

Plasmids expressing heterologous targets were constructed by cloning the coding regions for human or parasite DHFRs, NMTs or PGKs downstream of the TetO2 of pCM188 (between the *Bam*HI and *Pst*I sites), thus permitting regulatable expression of the target (addition of 2–20 mg l^−1^ of doxycycline to the growth medium results in a progressively lower expression from the promoter). The strain expressing the drug-resistant *P. vivax* DHFR (PvRdhfr) was constructed by mutating the following sites of the target enzyme: S58R, S117N and I173L. The plasmid was transformed into a *S. cerevisiae* yeast strain with a *dfr1Δ/DFR1 pdr5Δ/PDR5* BY4743 background. The strain was sporulated and *MAT*α haploids were selected for drug screens (for description of all other strains, see the electronic supplementary material, tables S1 and S2; see also [28]). Fluorescent plasmids were constructed by replacing the coding region of yEmRFP from yEpGAP-Cherry [29] with Venus, CFP or Sapphire [30], and replacing the *URA3* marker with *HIS3* or *LEU2* (for plasmid sequences and maps, see the electronic supplementary material). Yeast transformation and plasmid recovery were performed using standard methods.

### 3.2. Growth conditions

Standard growth conditions comprised either YPD (2% peptone, 1% yeast extract and 2% glucose) or YNB-glucose (0.68% yeast nitrogen base without amino acids, 2% ammonium sulphate and 2% glucose) with the relevant supplements for all assays. Drug screens were performed with yeast strains growing in YNB-glucose supplemented with lysine. Yeast strains expressing heterologous DHFRs were grown in the presence of 5 mg l^−1^ of doxycycline.

### 3.3. Microscopy

Fluorescent cells were examined with an Olympus BX51 microscope using filters 41028 YGFP (Venus), 49001 ET CFP (CFP), 31043 SAP/UV GFP (Sapphire) and 41043 HcRED1 (mCherry).

### 3.4. Competition experiments

Growth assays in YNB-glucose liquid media were performed using a BMG Optima plate reader with the filters Venus (excitation 500 nm/emission 540 nm), CFP (440 nm/490 nm), Sapphire (405 nm/510 nm) and mCherry (580 nm/612 nm) to allow a good discrimination between different fluorophores. The initial gain was adjusted to 10 per cent and the assays were run at 30°C, with shaking, and measurements taken every 15 min for a total of 30 h.

### 3.5. High-throughput screening, assay using laboratory automation of mixed cultures

Pre-cultures were grown in selective medium (YNB-glucose without leucine, histidine, uracil or methionine) to stationary phase and 1 ml of each culture was inoculated into 100 ml of the same medium. Pools of three strains, each labelled by the expression of a different fluorescent protein, were incubated at 30°C, with shaking, for 4 h to ensure exponential growth. Doxycycline (5 µg ml^−1^) was then added to the culture to reduce expression of the target enzyme. The culture was attached to a Thermo Combi multidrop within the automation work cell. The culture was stirred throughout and the room temperature maintained at 23°C during assay plate creation.

### 3.6. Automated assay plate creation

Fifty nanolitres of each chemical library compound (Maybridge Hitfinder library of approximately 14 400 chemically diverse compounds) were transferred to Matrix 384-well black clear-bottom assay plates. Each well of the assay plates was then inoculated with 50 µl of the pooled yeast culture (final compound concentration of 10 µM). The assay plates then entered a read-and-incubate cycle to determine the growth kinetics. Fluorescence measurements were obtained using the high-resolution BMG Polarstar plate reader, which allowed the detection of fluorescence over a much larger dynamic range than that detectable using BMG Optima, hence avoiding problems owing to detector saturation. Full details of these automated procedures will be published elsewhere [31].

### 3.7. High-throughput screening, quantification of results

Fluorescence readings were stored in a relational database. To allow comparison between the fluorescence readings taken for different strains, we modelled the former as continuous curves. In fitting a curve to the data, we followed a data-oriented approach, whereby we approximate the curve by cubic spline polynomials rather than assuming a particular curve function (e.g. exponential curve). From these growth curves, biologically relevant parameters were extracted, such as lagtime, μmax and maximum cell density (see the electronic supplementary material; see also [32]).

Because yeast strains expressing parasite and human targets bear different fluorophores, distinct growth curves can be obtained for pooled strains within a microtitre well, based on the fluorescence intensity at the given wavelength versus time. As the heterologous yeast strains were labelled with fluorescent proteins expressed from 2µ plasmids (which result in copy number variation between different cells in the population), all assay plates contained a number of control wells with the pool of yeast strains grown in the absence of the test drugs, so that the fluorescence intensity in each particular experiment could be internally controlled for and normalized.

The strain minimum doubling time (inversely proportional to the maximum growth rate) and biomass yield (net change in fluorescence from the beginning to the end of the assay) were calculated from each fitted growth curve. The yield was divided by the minimum doubling time to give a fitness score for the strain in the presence of the given drug. Within each well, the fitness of the strains expressing either parasite target (‘parasite’) was divided by the fitness score of the strain expressing human target (‘human’) present in the same well, to give a relative fitness indicating the specificity of the drug to the parasite target. Wells in which all of the strains exhibited severely compromised growth were removed from the analysis since they indicated either a technical problem with the well (e.g. autofluorescence of the drug) or a drug toxic to yeast itself.

For each microtitre plate, average fitness scores were calculated across the DMSO-only control wells. The s.d. of the fitness scores of all of the non-control wells was calculated for the plate. Where the ratio of the ‘parasite’-to-‘human’ fitness scores was more than three plate standard deviations smaller than the control-well value, the drug was deemed to be a putative hit against the given parasite target. From these candidates, if the ‘parasite’ fitness was less than 50 per cent of the ‘human’ fitness, the compound was added to the list of hits.

Using these criteria, a list of hits was assembled for each of the parasitic targets (see the electronic supplementary material, spreadsheet S1). The statistical significance of the overlap between the hit lists for different targets was calculated by applying the normal approximation to the hypergeometric distribution, given the number of hits for each parasite target, and the total number of hits and compounds screened (see the electronic supplementary material, spreadsheet S2).

### 3.8. *Trypanosoma brucei* viability assay

We selected 36 Maybridge compounds classified as *T. brucei* moderate/strong hits (growth of (strain expressing parasite target)/(strain expressing the host target) ≤ 0.4) for validation using intact bloodstream form (BSF) parasites (Lister 427). A total of 1 × 10^5^ BSF parasites were seeded into 24-well polystyrene plates in 1 ml HMI-9 supplemented with 10 per cent foetal bovine serum, 100 U ml^−1^ penicillin, 100 U ml^−1^ streptomycin and 10 µM of test compounds (or 10 µM blasticidin as a positive control). Cultures were grown in 5 per cent CO_2_ at 37°C for 48 h, when scoring the effect of the hit compounds on parasite growth was performed by counting parasite concentrations using a haemocytometer. Titration assays were then performed on compounds that killed all parasites at 10 µM, at the following concentrations: 10 µM, 1 µM, 100 nM, 10 nM and 1 nM. Some 1 × 10^6^ BSF parasites (Lister 427) were seeded into 10 ml HMI-9 (supplemented as above) and 1 µM, 100 nM or 10 nM of compound (as well as a no drug control) and were incubated in 25 cm^2^ non-adherent flasks with vented caps in 5 per cent CO_2_ at 37°C for 48 h. Proliferation was determined at 24 and 48 h by performing cell counts using a haemocytometer. A similar assay was performed using *T. brucei* EATRO 1125 grown in HMI-9 supplemented with 10 per cent rabbit serum, 100 U ml^−1^ penicillin and 100 U ml^−1^ streptomycin.

### 3.9. Validation of hits by structural similarity

Calls to Open Babel [33] calculated Tanimoto similarity coefficients between pairs of SMILES strings, using the ‘Daylight-like’ hashed FP2 fingerprinting method, which were then subtracted from 1 to give Tanimoto distance coefficients. Pairwise comparison matrices of Tanimoto distance coefficients were clustered hierarchically in R using the built-in ‘hclust’ function. Clustered pairwise comparison matrices were represented as heatmaps using the ‘heatmap.plus’ function, which displays colour-coded matrices alongside rows and columns.

Clustered pairwise comparison matrices were divided into discrete clusters using R's ‘cut’ function, which cuts dendrograms at specific heights or into specified numbers of clusters. For each cluster, the Small Molecule Subgraph Detector Toolkit [34] passed the maximal common subgraph (MCS) of each cluster to Open Babel, which wrote an .svg image showing the structures of molecules in the cluster with the MCS highlighted.

The three sets of *Plasmodium falciparum* whole-cell screening hits contained in the ChEMBL-NTD archive (https://www.ebi.ac.uk/chemblntd, accessed 20/01/2012) were combined, and duplicates deleted. Molecular structures, from both the Maybridge Hitfinder library and the ChEMBL-NTD archive, were retrieved and handled as SMILES (Simplified molecular-input line-entry system) strings [35].

## 4. Results

### 4.1. The fluorescent-yeast competition assay

We have constructed a compound-screening system that can be multiplexed. A number of yeast strains, each expressing a target protein for a different human parasite, can be grown in competition in a single well of a microtitre tray, together with a strain expressing the equivalent human protein. This serves a number of purposes. First, it increases the throughput of the screen and allows compounds with the potential to treat multiple diseases to be identified. Second, it enables the initial selection of drug candidates to be made by identifying compounds that significantly inhibit the growth of yeast cells expressing the parasite target, without inhibiting the growth of yeast expressing the equivalent human enzyme. Moreover, any compound that exhibits general cytotoxicity is identified by its inhibition of all the recombinant strains in the well, irrespective of which target proteins they express. Finally, the competition for nutrients between the different strains in the well amplifies the growth rate differences between them. Moreover, well-to-well variation in growth rate (an inherent problem of microtitre-plate growth assays) is rendered irrelevant because each well is internally controlled.

**Figure 1.**
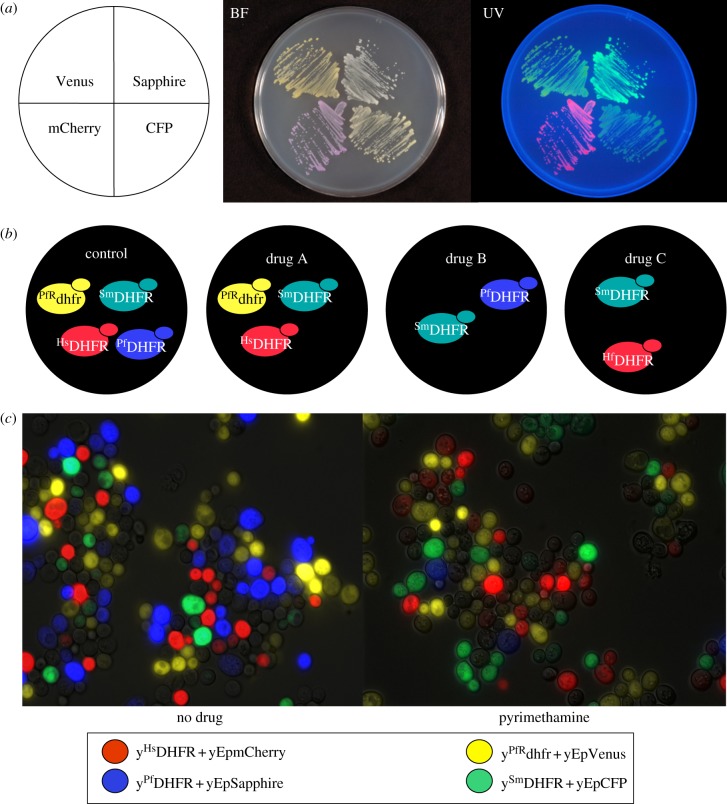
Fluorescence labelling of yeast strains. (*a*) Wild-type yeast transformed with plasmids expressing Venus (yellow fluorescent protein), Sapphire (blue fluorescent protein), mCherry (red fluorescent protein) or CFP (cyan fluorescent protein); visualized under bright field (BF) or ultraviolet (UV) light. (*b*) Schematic view of the experimental design developed for high-throughput screens: yeast strains expressing heterologous drug-resistant *Plasmodium falciparum* DHFR (^PfR^dhfr), *Schistosoma mansoni* DHFR (^Sm^DHFR), human DHFR (^Hs^DHFR) or *P. falciparum* DHFR (^Pf^DHFR) growing in the presence of candidate anti-parasitic drugs. (*c*) Pictures of fluorescently labelled yeast strains (expressing the indicated heterologous DHFRs) grown in competition in the presence or absence of the anti-malarial pyrimethamine.

To these ends, we constructed a series of yeast multicopy plasmids encoding a different fluorescent protein (mCherry, CFP, Venus or Sapphire [29,30]) from the strong *TDH3* promoter and carrying different nutritional markers. Owing to the high plasmid copy number and the *TDH3* promoter, expression of the fluorescent proteins was sufficient to allow visualization of different colony colours by the naked eye with or without UV illumination (figure 1*a*). The use of plasmids to carry the genes for the fluorescent proteins means that they can easily be swapped between strains expressing the different target proteins in order to control for any growth rate differences engendered by the expression of the fluorescent proteins themselves. The data from internal controls indicated that there were no problems with plasmid stability or copy number. However, if such problems did arise, integration of the genes for the fluorescent proteins into a yeast chromosome would be an alternative.

In our pilot experiment, we engineered strains in which the deletion of the essential yeast gene *DFR1,* which encodes DHFR, is complemented by the overexpression of DHFR coding sequences (cds) from *Homo sapiens* (^Hs^DHFR), *P. falciparum* (^Pf^DHFR), pyrimethamine-resistant *P. falciparum* (^PfR^dhfr) and *Schistosoma mansoni* (^Sm^DHFR) [28]. These cds were each placed under the control of the TetO2 promoter [36] such that they are downregulatable by the addition of doxycycline to the culture medium. Each of these strains was tagged with a different fluorescent protein (mCherry, Sapphire, Venus and CFP, respectively) [29,30], enabling them to be distinguished in a fluorescence assay for growth (figure 1*b*). It should be noted that, in these pilot experiments, fluorescence measurements were obtained using a BMG Optima plate reader, which has a limited dynamic range. For the high-throughput screens, we used a high-resolution plate reader (BMG Polarstar); this has a much larger dynamic range and avoids problems owing to detector saturation. The sensitivity to the anti-malarial drug pyrimethamine of strains expressing the wild-type *P. falciparum* DHFR and Sapphire fluorescent protein was verified (figure 1*c*).

**Figure 2.**
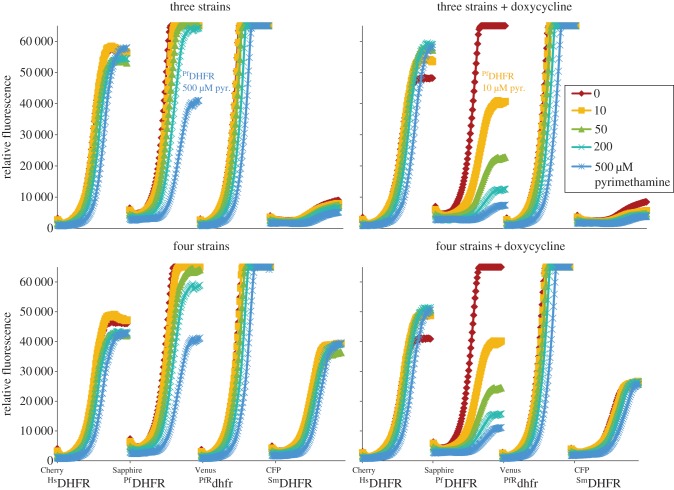
Relative fluorescence measure detected using a BMG Optima plate reader at 580 nm (excitation)/612 nm (emission) (Cherry), 405 nm (excitation)/510 nm (emission) (Sapphire), 500 nm (excitation)/540 nm (emission) (Venus) and 440 nm (excitation)/490 nm (emission) (CFP) of pooled yeast strains grown for 24 h in the presence of 0 to 500 μM pyrimethamine and 0 or 5 μg ml^−1^ of doxycycline. This plate reader has a limited dynamic range, and a higher-resolution instrument was used for the high-throughput screens. *Plasmodium falciparum* DHFR (^PfR^dhfr) labelled with Venus, *S. mansoni* DHFR (^Sm^DHFR) labelled with CFP, human DHFR (^Hs^DHFR) labelled with mCherry and *P. falciparum* DHFR (^Pf^DHFR) labelled with Venus.

To evaluate the performance of the fluorescent-yeast competition assay in a format suitable for high-throughput screens, we tested the pyrimethamine sensitivity of pools of three or four strains, each expressing a different fluorescent protein. This demonstrated that effective discrimination between the growth characteristics of the fluorescently labelled strains growing in competition had been achieved (figure 2). In addition, we determined whether the sensitivity of the assay could be increased by reducing the expression of the cds specifying the drug target by addition of 5 µg ml^−1^ of the TetO2 repressor, doxycycline. It was found that this treatment increased the pyrimethamine sensitivity of the yEpSapphire_HIS/y^Pf^DHFR strain by at least 50-fold (figure 2), which agrees with our previous results for plate assays [28].

### 4.2. High-throughput screening of a compound library using laboratory automation

**Figure 3.**
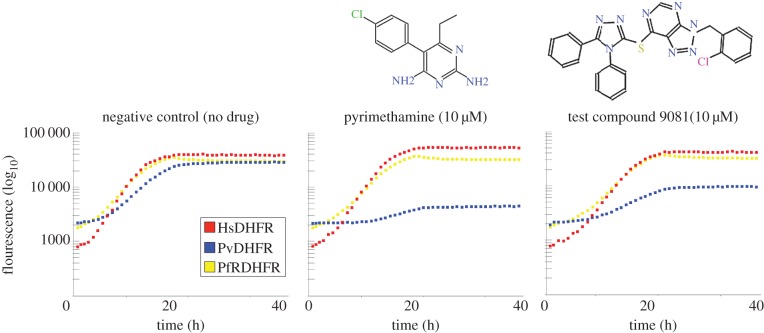
Example of a high-throughput screening result. Relative fluorescence measure detected using a BMG Polarstar plate reader at 580 nm (excitation)/612 nm (emission) (Cherry), 405 nm (excitation)/ 510 nm (emission) (Sapphire) and 500 nm (excitation)/540 nm (emission) (Venus) of pooled yeast strains grown in the presence of 5 μg ml^−1^ of doxycycline AND 10 μM pyrimethamine, 10 μM of test compound or no drug. *Plasmodium vivax* DHFR labelled with Sapphire (blue squares), human DHFR labelled with mCherry (red squares) and drug-resistant *P. falciparum* DHFR (PfRdhfr) labelled with Venus (yellow squares).

Using laboratory automation [37] we screened pools of three strains (two expressing parasite targets and one expressing the human orthologue) labelled with mCherry, Venus or Sapphire. The screens were performed in the presence of 5 µg ml^−1^ doxycycline and a library compound concentration of 10 µM (chemically diverse Maybridge Hitfinder library). Following data acquisition for each of the fluorophores, growth curves were generated (examples of growth curves derived from fluorescence measurements from three wells of one representative screen can be seen in figure 3) and smoothed, and growth scores (minimum doubling time and yield) were ascribed to each of the strains. Comparisons of the growth scores for each compound–strain combination allowed us to identify auto-fluorescent compounds or compounds that target the fluorescent marker proteins and not the parasite target. Problem wells, as well as compounds that exhibit general cytotoxicity, were also recognized. Finally, compounds that were active against the parasite target, but had no significant effect on yeast expressing the equivalent human protein, were designated as ‘hits’ (see the electronic supplementary material, spreadsheet S1).

For different screens against the same parasitic target, the overlap between the hits defined in this way is highly significant (*p* ≪ 10^−14^), demonstrating the reproducibility of our screening method. In addition, instances of significant overlap between the hits for the same molecular target in *different* parasites reflect the relatedness of the two species (see the electronic supplementary material, spreadsheet S2). This reaffirms that our hit compounds are in fact specific for some feature of the parasite target, conserved only between closely related species. *Plasmodium vivax* and *P. falciparum*, and *Trypanosoma* *cruzi* and *T. brucei* DHFRs share multiple hits, as do WT and drug-resistant *P. vivax* and *P. falciparum.* These latter compounds could represent promising leads in addressing the drug-resistance problem.

### 4.3. Validation of confirmed hit compounds by demonstrating their action against *Trypanosoma brucei* in culture

**Figure 4.**
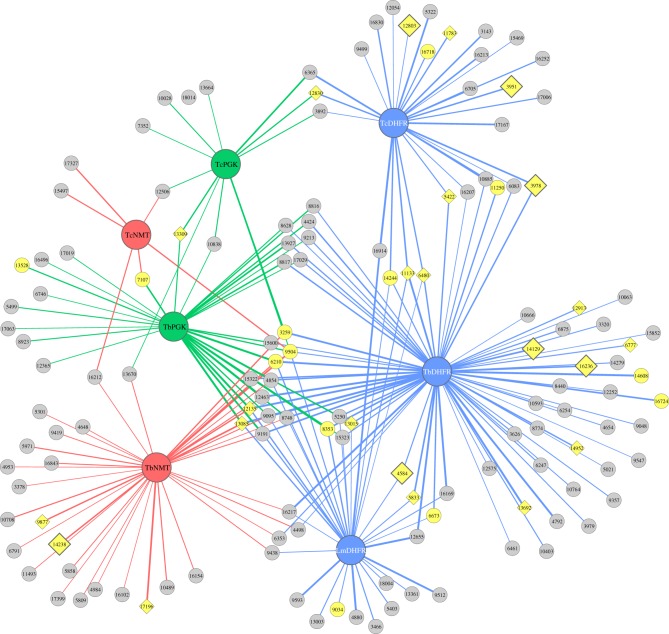
Network of connections between kinetoplastid targets and our hits. Overview of the compounds from the Maybridge hitfinder library identified as specific inhibitors of the following parasite (*Trypanosoma brucei*, Tb; *T. cruzi*, Tc; *Leishmania major*, Lm) targets: dihydrofolate reductase (DHFR), blue; *N*-myristoyltransferase (NMT), red; phosphoglycerate kinase (PGK), green. Small nodes represent hits; yellow nodes represent compounds tested in *T. brucei* cultures. Diamond nodes represent compounds active in *Trypanosoma* at 10 µM. Large diamonds represent compounds active in *T. brucei* cultures at 1 µM or less. The thickness of the edges (lines connecting the targets and compounds) represents the strength of the inhibition (thicker lines indicate stronger inhibition of growth of the yeast expressing the indicated parasite target by the connecting compound).

We selected 36 hits against yeast strains encoding *T. brucei*, *T. cruzi* or *Leishmania major* targets for validation using intact *T. brucei* parasites. 18 of the tested compounds (50%) were able to kill *T. brucei* Lister 427 bloodstream form parasites at 10 µM (after 48 h) and five additional compounds were responsible for a severely reduced parasite yield (figure 4 and table 1). The drugs capable of killing the parasite at 10 µM were tested in titration experiments to determine the minimum concentration necessary to kill *T. brucei* Lister 427 parasites. All of the 10 µM hits were confirmed and seven of the compounds showed some effect at 1 µM, four were effective at 100 nM and two were effective at 10 nM (table 1).

**Table 1.** Hit validation in *Trypanosoma brucei.* An initial (qualitative) estimate of the anti-parasitic activity of each compound was made as follows: D, dead (no viable parasites detected); S, sick (anomalous morphology) or slow growth; L, live (morphology and growth comparable to ‘no drug’ control). Those compounds that appeared active at less than or equal to 1 µM were then subjected to a quantitative analysis of their growth inhibitory effects: % growth after 24 or 48 h = 100 × (cell count, with drug)/(cell count, no drug control).

*T. brucei* target	Compound ID	Lister 427 (qualitative growth score)				Lister 427 % growth after 24 or (48 h)			EASTRO 1125 % growth after 24 or (48 h)		
		10 µM	1 µM	100 nM	10 nM	1 µM	100 nM	10 nM	1 µM	100 nM	10 nM
DHFR, NMT	3259	L									
(^Tc^DHFR)	3951	D	D	S	L	0 (0)	34 (7)	80 (88)	22 (2)	50 (18)	73 (80)
DHFR	3978	D	S	L	L	40 (0.2)	79 (57)	101 (64)			
DHFR	4584	D	D	S	S	0 (0)	5 (0.2)	36 (46)	0 (0)	0 (0)	14 (0.7)
DHFR	5422	D	L	L	L						
DHFR	5833	D	L	L	L						
DHFR, PGK, NMT	6210	L									
DHFR	6480	D	L	L	L						
DHFR	6673	L									
DHFR	6777	D	L	L	L						
PGK	7107	L									
PGK, DHFR	8353	L									
(^Lm^DHFR)	9034	L									
DHFR, PGK, NMT	9504	L									
NMT	9877	S									
DHFR	11133	D	L	L	L						
DHFR	11250	L									
(^Tc^DHFR)	11783	D	L	L	L						
DHFR, PGK, NMT	12135	D	L	L	L						
(^Tc^DHFR)	12803	D	D	L	L	20 (26)	89 (88)	111 (88)			
(^Tc^DHFR, ^Tc^PGK)	12830	D	L	L	L						
DHFR	12913	S									
DHFR, PGK	13015	D	L	L	L						
DHFR, PGK, NMT	13085	S									
PGK	13309	S									
PGK	13528	L									
DHFR	13692	S									
DHFR	14129	D	D	D	S	0 (0)	0 (0)	20 (68)	0 (0)	0 (0)	0 (0)
NMT	14238	D	D	L	L						
DHFR	14244	L									
DHFR	14608	L									
DHFR	14952	D	L	L	L						
DHFR	16236	D	D	S	L	0 (0)	32 (0)	97 (83)	43 (65)	65 (88)	76(113)
(^Tc^DHFR)	16718	L									
DHFR	16724	L									
NMT	17196	D	L	L	L

To better quantify our anti-trypanosomal compounds, we followed the growth of *T. brucei* Lister 427 (a monomorphic laboratory isolate) and EATRO 1125 (a pleomorphic isolate with limited passage history) in the presence of 1 μM, 100 nM or 10 nM of 6 or 4 (respectively) different hit compounds. We observed that compounds ID_4584(1-methyl-2-[3-(1-methyl-1,2-dihydroquinolin-2-yliden)prop-1-enyl]quinolinium iodide) and ID_14129 (2,4-dichloro-1-(2-nitrovinyl)benzene) could kill virtually all parasites after 48 h at concentrations as low as 10 nM (table 1).

### 4.4. Chemoinformatic validation of hit compounds

We compared drug hits against different parasite targets (identified by screening the Maybridge Hitfinder library using our fluorescence assay) with each other to identify structural features associated with activity against a particular target. First, molecular structures were represented as ‘fingerprints’ [38], bitstrings encoding the structural features present in a molecule and then the dissimilarities between pairs of fingerprints were quantified as Tanimoto distance coefficients (TDC). Possible TDC values range continuously from 0 (indicating identical fingerprints) to 1 (indicating completely dissimilar fingerprints). We constructed a pairwise Tanimoto distance matrix for our ‘hits’ using the Open Babel FP2 fingerprint.

We performed hierarchical clustering on this matrix to generate a ‘heatmap’ of the pairwise similarities between our hits (see the electronic supplementary material, figure S1), with hits for all parasites and targets on each axis. This showed several clusters of similar structures. Additionally, there appeared to be a correlation between these clusters and activity against particular targets. For example, five compounds contained in a tight cluster all showed activity against PfRdhfr exclusively (see the electronic supplementary material, figure S1).

The dataset dendrogram was then ‘cut’ at different levels, and the ‘MCS’ [39], or largest structural feature shared by all members of a subset, was calculated for each cluster. The size and complexity of this feature, as well as the sizes and number of constituent compounds, provide a qualitative insight as to the quality of clustering, as well as the significant structural features.

At a cut level of 0.65 we obtained clusters that showed clear distinguishing MCSs, such as for a cluster of hits showing specificity against the PfDHFR and PfRdhfr targets (see the electronic supplementary material, figure S2). The MCS may represent the defining feature of a cluster, but there is more information that can be obtained. For example, the cluster of compounds displaying specificity for PfRdhfr alone (see the electronic supplementary material, figure S2) has an oxime ester as its MCS. From inspection of the structures, it is apparent that subdivisions of the cluster would have more complex common features. These are therefore features that might work to increase the activity or specificity of a compound, in tandem with the MCS.

**Figure 5.**
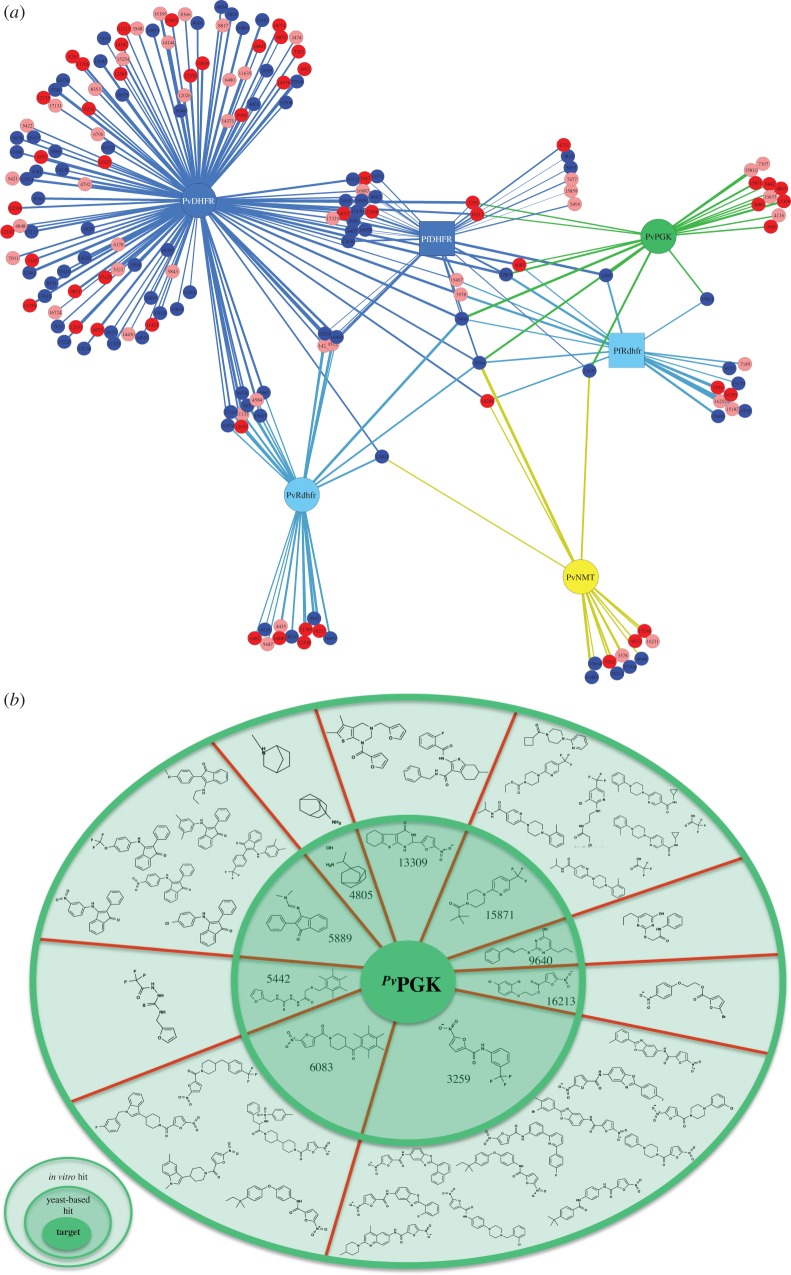
Antiplasmodial hits. (*a*) Network of connections between drug targets and our *Plasmodium* hits. Overview of the compounds from the Maybridge hitfinder library identified as specific inhibitors of the following parasite (*P. falciparum*, Pf, square nodes; *P. vivax*, Pv, large circular nodes) targets: dihydrofolate reductase (DHFR), blue; drug-resistant dihydrofolate reductase (Rdhfr), light blue; *N*-myristoyltransferase (NMT), yellow; phosphoglycerate kinase (PGK), green. Small nodes represents hits with Tanimoto distance coefficients of  less than 0.4 (red), less than 0.5 (pink) or greater than 0.5 (blue) to Chemblntd compounds identified in *P. falciparum in vitro* screens (www.ebi.ac.uk/chemblntd). (*b*) Chemblntd compounds (compounds with demonstrated activity against *P. falciparum* in *ex vivo* assays) and similar hits identified in our screens as potentially targeting PvPGK. Schematic depicting the structures of PvPGK-specific compounds identified in screens and similar compounds with demonstrated activity against *P. falciparum*.

Encouraged by the correlation between the structural similarities of the hit compounds and their specific activity against the parasite targets, we performed a similar TDC analysis on the *Plasmodium* hits (*P. falciparum*: DHFR and drug-resistant DHFR; *P. vivax*: DHFR and drug-resistant DHFR, NMT and PGK) against the library of anti-plasmodial compounds identified by GlaxoSmithKline, Novartis and St Jude Children's Hospital (www.ebi.ac.uk/chemblntd) using screens against cultures of *P. falciparum*. We found that 54 per cent of the compounds that our screen identified as differentially active against *P. falciparum* had distance coefficients of 0.5 or lower to at least one of the anti-plasmodial compounds; and 29 per cent of the *Plasmodium* hits had distance coefficients of 0.4 or lower (see figure 5*a* and electronic supplementary material, spreadsheet S3). We then arranged our anti-plasmodial hits based on their similarity and plotted these compounds in a similarity heatmap against the Chemblntd compounds that had a TDC of 0.4 or lower to our hits. In this manner, we could identify structural groups with demonstrated *in vitro* anti-plasmodial activity, as well as suggest the intracellular targets for a few of the Chemblntd compounds (see figure 5*b* and electronic supplementary material, figure S3).

Our screens identified some compounds that were pleiotropic in their anti-parasitic target effects. For instance, ID_3259 (N2-[3-(trifluoromethyl)phenyl]-5-nitro-2-furamide) was effective against NMT, PGK and DHFR targets from different parasite species (see figures 4 and 5, and electronic supplementary material, spreadsheet S1). It is possible that such ubiquitous activity represents some experimental artefact. However, this concern was mitigated by the identification of a large number of Chemblntd compounds with TDC scores less than 0.5 to ID_3259 (see electronic supplementary material, figure S5 and spreadsheet S3). Our study may thus have defined a novel chemical scaffold on which to base broad-spectrum anti-parasitic drugs.

## 5. Discussion

We have developed a fully automated drug-screening method based on engineering yeast to express parasite drug targets or their human counterparts. The assay exploits the fluorescent labelling of yeast cells to allow the growth of three to four different strains in competition in the presence of different library compounds. This approach provides high sensitivity (owing to competition between strains), minimizes plate-position effects and provides an internal control for general cytoxicity. This approach is fast, cheap and more flexible than drug screens against parasites in culture.

In this paper, we have reported the basis of our fluorescent-yeast system and the results of a primary screen carried out by using the Maybridge hitfinder library of compounds. We chose to demonstrate the utility of using the system to screen for drug candidates related to neglected tropical diseases (NTDs) for two main reasons: their medical and societal importance, and their tractability to drug discovery. NTDs including schistosomiasis (caused by *Schistosoma* spp.), leishmaniasis (*Leishmania* spp.), sleeping sickness (*T. brucei*) and Chagas disease (*T. cruzi*) kill over half a million people every year, a similar burden of disease to malaria (*Plasmodium* spp.) [40]. These diseases affect the poorest populations in Africa, Asia and Latin America. Conservative estimates indicate an annual loss of 57 million disability-adjusted life years owing to NTDs [40]. Collaborations between not-for-profit organizations and for-profit companies are developing new drug-screening methods and identifying promising new anti-parasitic compounds [41–44]. While these recent advances are encouraging, the screens are generally limited to a specific developmental stage of the target parasite.

A great strength of the system described in this work is that it enables screens against targets from parasites at any of their life-cycle stages, even if some or all of these are unculturable. Thus, it combines most of the advantages of cell-based and biochemical screens. Moreover, we have demonstrated its ability to identify compounds that can kill the target parasite by testing a subset of our anti-kinetoplastid compounds *in vitro* against *T. brucei* (which causes African sleeping sickness in humans, and nagana in cattle) and observed that over 60 per cent of our hits can successfully kill or severely inhibit growth of the parasites (figure 4 and table 1). Having performed *T. brucei in vitro* drug assays for 36 very diverse compounds, with the aid of hierarchical clustering of our hits (see the electronic supplementary material, figure S1), we are now in a position to prioritize further compounds for validation in parasites.

Some compounds that were highly active against *T. cruzi* DHFR (e.g. ID_3951 (O1-[(5-nitro-2-furyl)carbonyl]-4-[2-nitro-4-(trifluoromethyl)phenoxy]benzene-1-carbohydroximamide) and ID_12803 (4-(tert-butyl)phenyl 5-nitrothiophene-2-carboxylate)) in the fluorescent-yeast system, but not selected as ‘hits’ against *T. brucei* targets (probably due to our very stringent selection threshold), nevertheless showed activity against *T. brucei* in our *in vitro* assay (figure 4 and table 1). This suggests that these compounds have the potential to be used against multiple kinetoplastids. We also noticed that compounds active against the *Trypanosoma* gPGK (AAA32121.1) isoform screened in our system were poorly validated in *in vitro* parasite-based assays, suggesting that this enzyme might not be important for this particular parasite life-cycle stage. However, this isozyme might still be essential for a different stage of the parasite's life cycle. Therefore, the potential of our TbPGK hits as novel anti-parasitic agents should not be dismissed before validation experiments using alternative parasite life forms have been carried out.

Unlike the *T. brucei* PGK drug hits, the *P. vivax* PGK hits had an excellent *in silico* validation rate, with all of the PvPGK-only hits showing a TDC of 0.5 or less to compounds validated in *P. falciparum in vitro* screens (see figure 5 and electronic supplementary material, figure S3). This is not surprising as *Plasmodium* genomes, like that of yeast, encode only one PGK isoform. Hence, we provided good evidence supporting *Plasmodium* PGK as a promising drug target, and suggested a number of Chemblntd compounds for target-based validation in the fluorescent-yeast system, as well as in biochemical assays using recombinant *P. falciparum* and *P. vivax* PGKs.

We have demonstrated the success of the fluorescent-yeast method in identifying compounds that have high specific activity against a range of drug targets from different parasites (see the electronic supplementary material, spreadsheet S1). In all cases, the system provided assurance that the compound did not inhibit the biological activity of the corresponding human enzyme. The compounds identified as active against targets from parasites (in particular, against those from *P. vivax,* for which we obtained the largest number of hits) have great potential for use as scaffolds for further chemical syntheses.

The approach described in this work is flexible enough to be used in screens for drugs against many other parasites or bacterial pathogens, or to screen for compounds specific to particular isoforms of human proteins [45]. We have demonstrated that this approach can work synergistically with current parasite-based high-throughput screening methods. It can identify chemical scaffolds for further development by the pharmaceutical industry [42], and also suggest the mechanism of action of compounds identified in pathogen-based screens. We believe that such synthetic biology screens based on classic model organisms could provide a powerful new weapon in the armoury of drug discovery and development.

## 6. Acknowledgements

We thank Neta Dean (Stonybrook, NY) for the yEpGAP-Cherry plasmid. We thank Katherine Martin (RDK laboratory) for technical assistance in running the HTPs. We thank Balázs Papp (Biological Research Center of the Hungarian Academy of Sciences and Cambridge Systems Biology Centre) for critical reading of the manuscript. This work was supported by grant no. BB/F008228/1 from the UK Biotechnology and Biological Sciences Research Council to S.G.O. and R.D.K., and a contract from the European Commission under the FP7 Collaborative Programme, UNICELLSYS to S.G.O.

## Supplementary Material

Supplementary material - Bilsland et al.

## Supplementary Material

Supplementary Spreadsheet S1

## Supplementary Material

Supplementary Spreadsheet S2

## Supplementary Material

Supplementary Spreadsheet S3
