# Supplementary material for: Yeast-based automated high-throughput screens to identify anti-parasitic lead compounds
Source: Open Biol. 2013 Feb;3(2):120158. doi: 10.1098/rsob.120158 (PMC3603448; doi:10.1098/rsob.120158)
Supplement: Supplementary material - Bilsland et al. [file rsob120158-s1.pdf]

Supplementary material – Bilsland et al.

Table of Contents

Supplementary table S1. Plasmids used in this study 2

Supplementary table S2. Strains used in this study........ 3

Supplementary figure S1. Structural similarities between our Maybridge Hitfinder hits. 5

Supplementary figure S2. Structures of clustered Nyfedge Maybridge Hitfinder hits. ... 6

Supplementary figure S3. Structural similarities between Maybridge Hitfinder antiplasmodial hits and Chemblntd antiplasmodial compounds. 7

Supplementary figure S4. Plasmid map of yEp_CFP_HIS3. 8

Supplementary figure S5. Plasmid map of yEp_CFP_LEU2. 8

Supplementary figure S6. Plasmid map of yEp_CFP_URA3. 9

Supplementary figure S7. Plasmid map of yEp_Cherry_HIS3. 9

Supplementary figure S9. Plasmid map of yEpGAP_ Cherry. 10

Supplementary figure S10. Plasmid map of yEp_Sapphire_HIS3. 11

Supplementary figure S11. Plasmid map of yEp_ Sapphire _LEU2. 11

Supplementary figure S12. Plasmid map of yEp_ Sapphire _URA3. 12

Supplementary figure S13. Plasmid map of yEp_Venus_HIS3. 12

Supplementary figure S14. Plasmid map of yEp_Venus_LEU2. 13

Supplementary figure S15. Plasmid map of yEp_Venus_URA3. 13

Supplementary plasmid sequences. Sequences of fluorescent plasmids described in this work. 14

Supplementary Spreadsheet S1. Nyfedge Maybridge Hits. 38

Supplementary Spreadsheet S2. Number Chemblntd compounds with similarities to Nyfedge's antiplasmodial hits at different thresholds. 38

Supplementary Spreadsheet S3. Overlap between compounds identified as hits in Nyfedge high-throughput screens. 38

Supplementary references........ 39

Supplementary table S1. *Plasmids used in this study*

Plasmid name	Features	Derived from	Marker	Source
pCM188	CEN plasmid; TetO2 promoter		*URA3*	[1]
pCM^{Pf}*DHFR*	$^{Pf}DHFR$	pCM188	*URA3*	[2]
pCM^{PfR}*dhfr*	Drug resistant $^{Pf}dhfr^{51I,59R,108N}$	pCM188	*URA3*	[2]
pCM^{Pv}*DHFR*	$^{Pv}DHFR$	pCM188	*URA3*	[2]
pCM^{PvR}*dhfr*	Drug resistant $^{Pv}dhfr^{58R,117N,172L}$	pCM188	*URA3*	This work
pCM^{Sm}*DHFR*	$^{Sm}DHFR$	pCM188	*URA3*	[2]
pCM^{Tb}*DHFR*	$^{Tb}DHFR$	pCM188	*URA3*	[2]
pCM^{Tc}*DHFR*	$^{Tc}DHFR$	pCM188	*URA3*	[2]
pCM^{Lm}*DHFR*	$^{Lm}DHFR$	pCM188	*URA3*	[2]
pCM^{Hs}*DHFR*	$^{Hs}DHFR$	pCM188	*URA3*	[2]
pCM^{Pv}PGK	^{Pv}PGK	pCM188	URA3	[2]
pCM^{Sm}PGK	^{Sm}PGK	pCM188	URA3	[2]
pCM^{Tb}PGK	^{Tb}PGK	pCM188	URA3	[2]
pCM^{Tc}PGK	^{Tc}PGK	pCM188	URA3	[2]
pCM^{Hs}PGK	^{Hs}PGK	pCM188	URA3	[2]
pCM^{Pv}NMT	^{Pv}NMT	pCM188	URA3	[2]
pCM^{Sm}NMT	^{Sm}NMT	pCM188	URA3	[2]
pCM^{Tb}NMT	^{Tb}NMT	pCM188	URA3	[2]
pCM^{Tc}NMT	^{Tc}NMT	pCM188	URA3	[2]
pCM^{Hs}NMT	^{Hs}NMT	pCM188	URA3	[2]
pKT90	yEVenus			[3]
pKT101	yECFP			[3]
pKT149	yESapphire			[3]
pRS423			*HIS3*	[4]
pRS425			*LEU2*	[4]
yEpGAP-Cherry	2μ; TDH3-promoter-driven yEmRFP		*URA3*	[5]
yEpVenus_URA	2μ; TDH3-promoter-driven Venus (YFP)	yEpGAP-Cherry	*URA3*	This work
yEpCFP_URA	2μ; TDH3-promoter-driven CFP	yEpGAP-Cherry	*URA3*	This work

yEpSapphire_URA	2µ; TDH3-promoter-driven Sapphire (BFP)	yEpGAP-Cherry	*URA3*	This work
yEpVenus_HIS	2µ; TDH3-promoter-driven Venus (YFP)	yEpVenus_URA	*HIS3*	This work
yEpCFP_HIS	2µ; TDH3-promoter-driven CFP	yEpCFP_URA	*HIS3*	This work
yEpSapphire_HIS	2µ; TDH3-promoter-driven Sapphire (BFP)	yEpSapphire_URA	*HIS3*	This work
yEpCherry_HIS	2µ; TDH3-promoter-driven yEmRFP	yEpGAP-Cherry	*HIS3*	This work
yEpVenus_LEU	2µ; TDH3-promoter-driven CFP	yEpVenus_URA	*LEU2*	This work
yEpCFP_LEU	2µ; TDH3-promoter-driven Sapphire (BFP)	yEpCFP_URA	*LEU2*	This work
yEpSapphire_LEU	2µ; TDH3-promoter-driven Sapphire (BFP)	yEpSapphire_URA	*LEU2*	This work
yEpCherry_LEU	2µ; TDH3-promoter-driven yEmRFP	yEpGAP-Cherry	*LEU2*	This work

Supplementary table S2. *Strains used in this study*

Strain name	Genotype	Plasmid	Source
BY4743	*MAT**a**/MATα his3Δ1/his3Δ1 leu2Δ0/leu2Δ0 met15Δ0/MET15 LYS2/lys2Δ0 ura3Δ0/ura3Δ0*		[6]
y^{Pf}DHFR_p	*dfr1Δ::KanMx pdr5Δ::HisMX his3Δ1 leu2Δ0 MET15 lys2Δ0 MATα*	pCM^{Pf}*DHFR*	[2]
y^{PfR}dhfr_p	*dfr1Δ::KanMx pdr5Δ::HisMX his3Δ1 leu2Δ0 MET15 lys2Δ0 MATα*	pCM^{PfR}*dhfr*	[2]
y^{Pv}DHFR_p	*dfr1Δ::KanMx pdr5Δ::HisMX his3Δ1 leu2Δ0 MET15 lys2Δ0 MATα*	pCM^{Pv}*DHFR*	[2]
y^{PvR}dhfr_p	*dfr1Δ::KanMx pdr5Δ::HisMX his3Δ1 leu2Δ0 MET15 lys2Δ0 MATα*	pCM^{PvR}*dhfr*	This work
y^{Sm}DHFR_p	*dfr1Δ::KanMx pdr5Δ::HisMX his3Δ1 leu2Δ0 MET15 lys2Δ0 MATα*	pCM^{Sm}*DHFR*	[2]
y^{Tc}DHFR_p	*dfr1Δ::KanMx pdr5Δ::HisMX his3Δ1 leu2Δ0*	pCM^{Tc}*DHFR*	[2]

	MET15 lys2Δ0 MATα		
y^{Tb}DHFR_p	*dfr1Δ::KanMx pdr5Δ::HisMX his3Δ1 leu2Δ0 MET15 lys2Δ0 MATα*	pCM^{Tb}*DHFR*	[2]
Y^{Lm}DHFR_p	*dfr1Δ::KanMx pdr5Δ::HisMX his3Δ1 leu2Δ0 MET15 lys2Δ0 MATα*	pCM^{Lm}*DHFR*	[2]
y^{Hs}DHFR_p	*dfr1Δ::KanMx pdr5Δ::HisMX his3Δ1 leu2Δ0 MET15 lys2Δ0 MATα*	pCM^{Hs}*DHFR*	[2]
y^{Pv}PGK_p	*pgk1Δ::KanMx pdr5Δ::HisMX his3Δ1 leu2Δ0 MET15 lys2Δ0 MATα*	pCM^{Pv}*PGK*	[2]
y^{Sm}PGK_p	*pgk1Δ::KanMx pdr5Δ::HisMX his3Δ1 leu2Δ0 MET15 lys2Δ0 MATα*	pCM^{Sm}*PGK*	[2]
y^{Tc}PGK_p	*pgk1Δ::KanMx pdr5Δ::HisMX his3Δ1 leu2Δ0 MET15 lys2Δ0 MATα*	pCM^{Tc}*PGK*	[2]
y^{Tb}PGK_p	*pgk1Δ::KanMx pdr5Δ::HisMX his3Δ1 leu2Δ0 MET15 lys2Δ0 MATα*	pCM^{Tb}*PGK*	[2]
y^{Hs}PGK_p	*pgk1Δ::KanMx pdr5Δ::HisMX his3Δ1 leu2Δ0 MET15 lys2Δ0 MATα*	pCM^{Hs}*PGK*	[2]
yPvNMT_p	*NMT1Δ::KanMx pdr5Δ::HisMX his3Δ1 leu2Δ0 MET15 lys2Δ0 MATα*	pCMPvNMT	[2]
ySmNMT_p	*NMT1Δ::KanMx pdr5Δ::HisMX his3Δ1 leu2Δ0 MET15 lys2Δ0 MATα*	pCMSmNMT	[2]
yTcNMT_p	*NMT1Δ::KanMx pdr5Δ::HisMX his3Δ1 leu2Δ0 MET15 lys2Δ0 MATα*	pCMTcNMT	[2]
yTbNMT_p	*NMT1Δ::KanMx pdr5Δ::HisMX his3Δ1 leu2Δ0 MET15 lys2Δ0 MATα*	pCMTbNMT	[2]
yHsNMT_p	*NMT1Δ::KanMx pdr5Δ::HisMX his3Δ1 leu2Δ0 MET15 lys2Δ0 MATα*	pCMHsNMT	[2]

Supplementary figure S1. *Structural similarities between our Maybridge Hitfinder hits.*

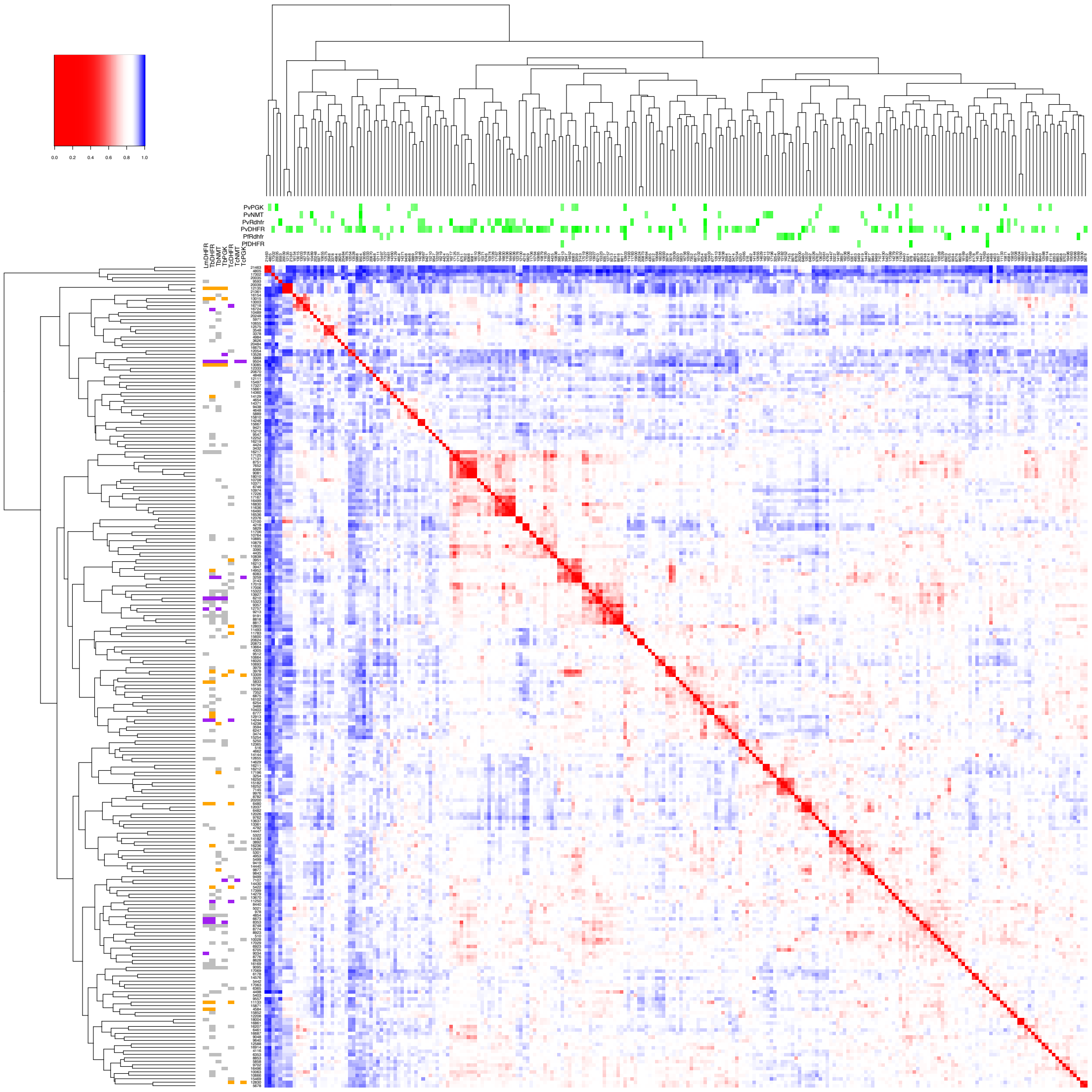

Hierarchically clustered heatmap of pairwise Tanimoto dissimilarity scores between our Maybridge Hitfinder hits against all targets and parasites. Structures were compared using the Open Babel FP2 fingerprint. Red colour indicates maximum similarity whereas blue indicates maximum distance. Left-hand colour matrix shows whether the compounds were active *in vitro* against *T.brucei* (orange), not validated (purple) or not tested (grey). Top colour matrix shows the degree of inhibition high and specific (green) or non-specific (white) of each hit compound against six *Plasmodium* targets screened.

Supplementary figure S2. *Structures of clustered Nyfedge Maybridge Hitfinder hits.*

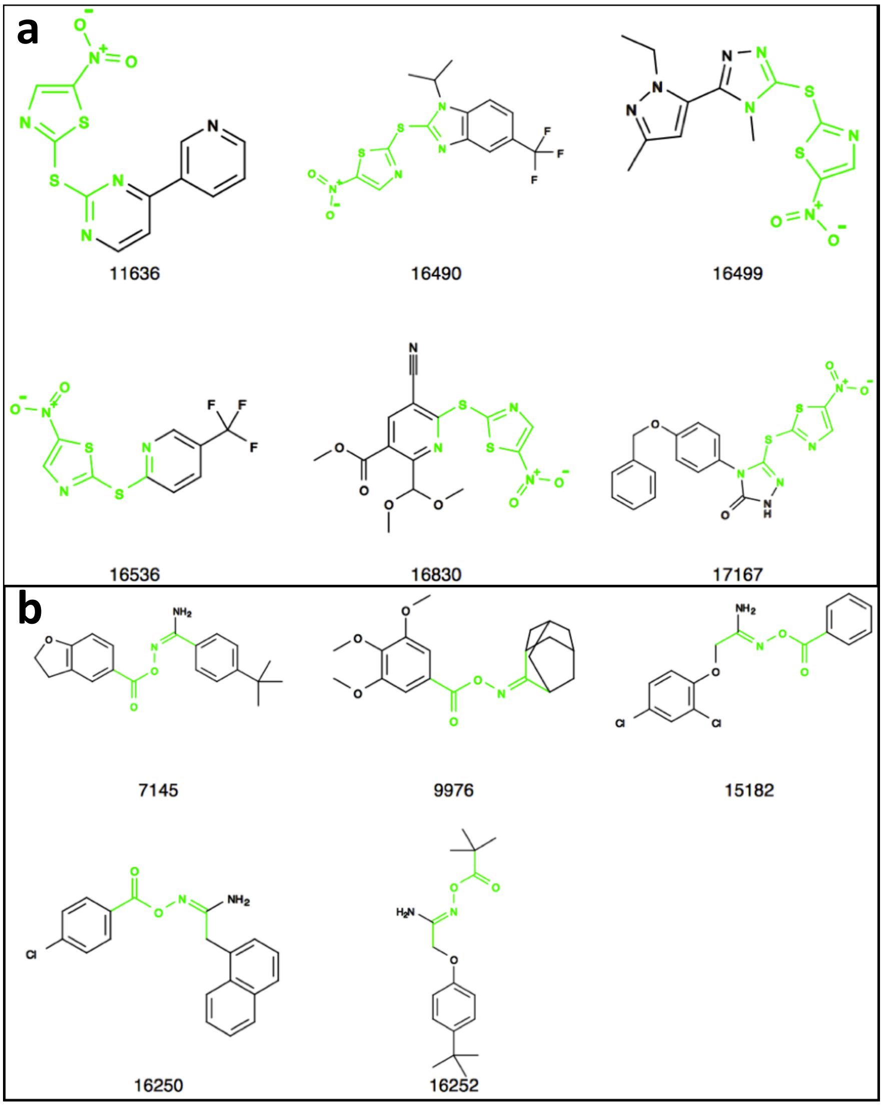

All Nyfedge Maybridge Hitfinder hits were hierarchically clustered, and the resulting dendrogram cut at a height of 0.65. The Maximum Common Subgraph of each cluster was determined, and highlighted in the structure of each compound contained in the cluster. (**a**) The structures of the six compounds contained in a cluster which all contain a nitrothiazole thioether subgroup. (**b**) The structures of the five compounds contained in a cluster which all exhibit specific inhibition of drug-resistant *P. falciparum* dhfr.

Supplementary figure S3. *Structural similarities between Maybridge Hitfinder antiplasmodial hits and Chemblntd antiplasmodial compounds.*

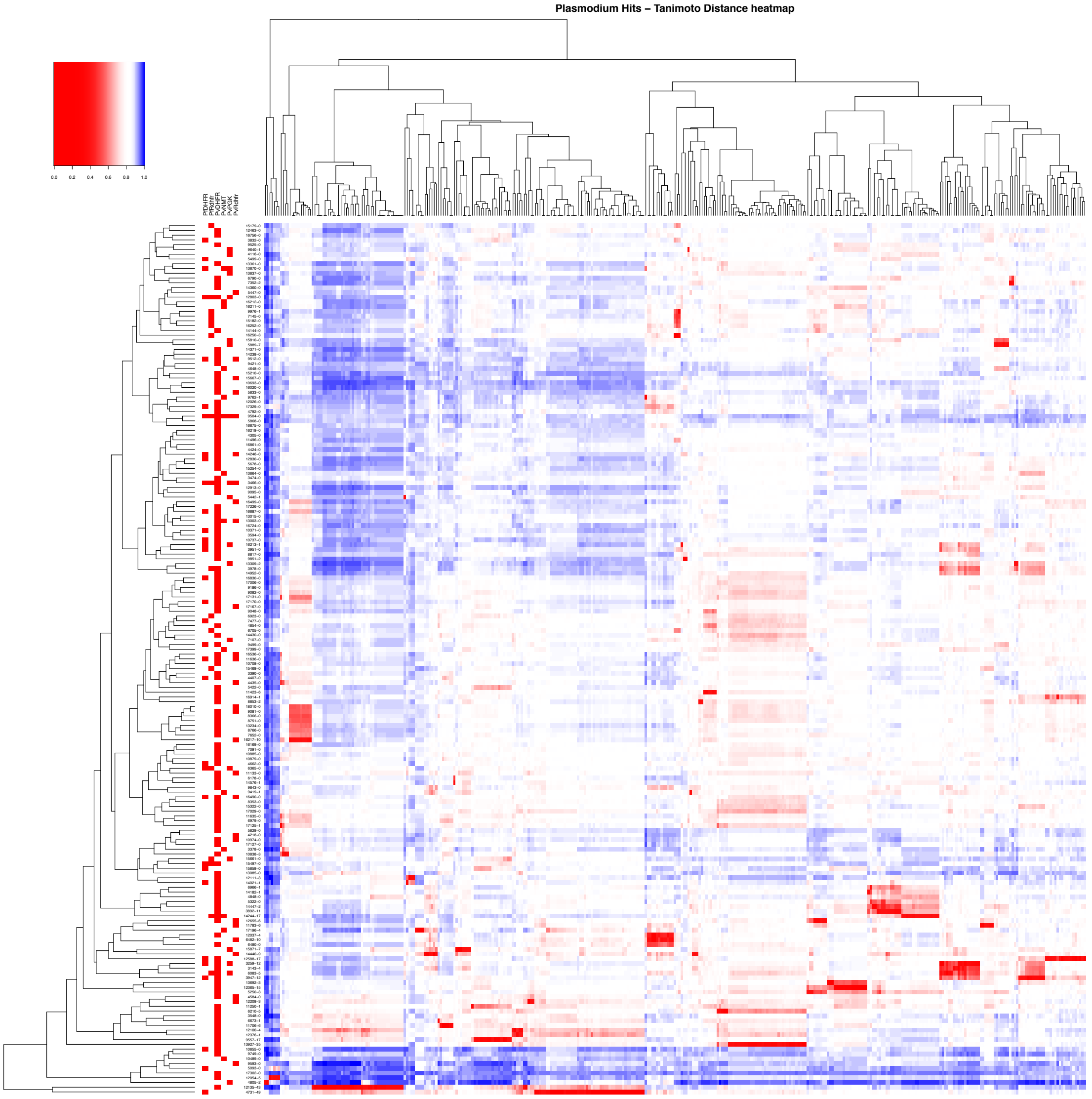

Hierarchically clustered heatmap of pairwise Tanimoto dissimilarity scores between Maybridge Hitfinder hits against *Plasmodium falciparum* (Pf) or *P.vivax* (Pv) dihydrofolate reductase (DHFR), drug resistant DHFR (Rdhfr), N-myristoyl transferase (NMT) or phosphoglycerate kinase (PGK). Structures were compared using the Open Babel FP2 fingerprint. Red colour indicates maximum similarity whereas blue indicates maximum distance. Left-hand colour matrix shows whether the specificity of compounds against each of the targets (in the Nyfedge system).

Supplementary figure S4. *Plasmid map of yEp_CFP_HIS3.*

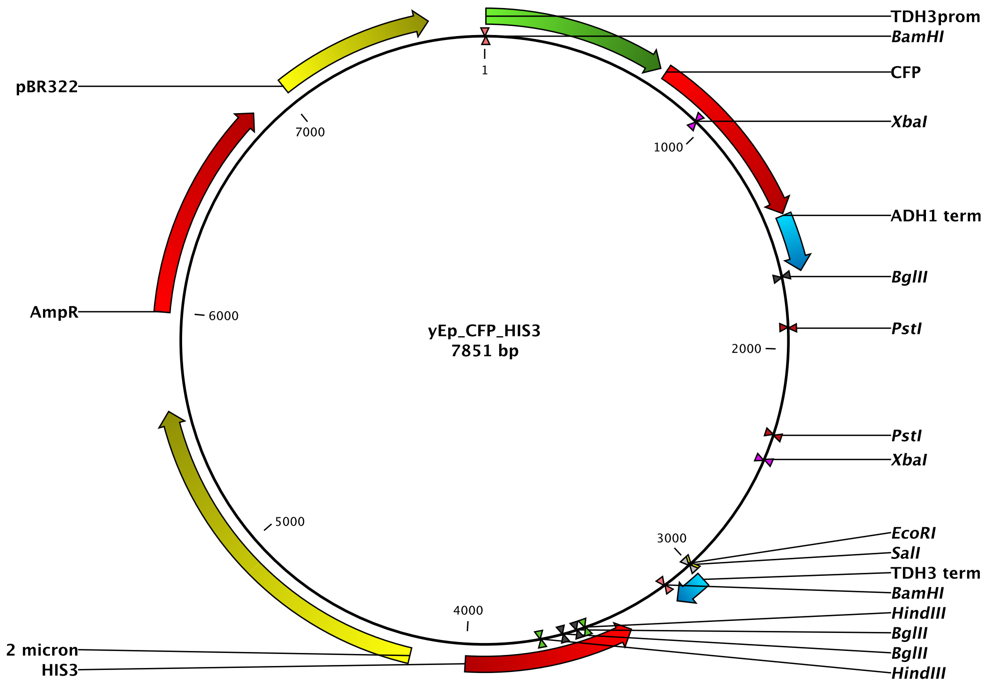

Supplementary figure S5. *Plasmid map of yEp_CFP_LEU2.*

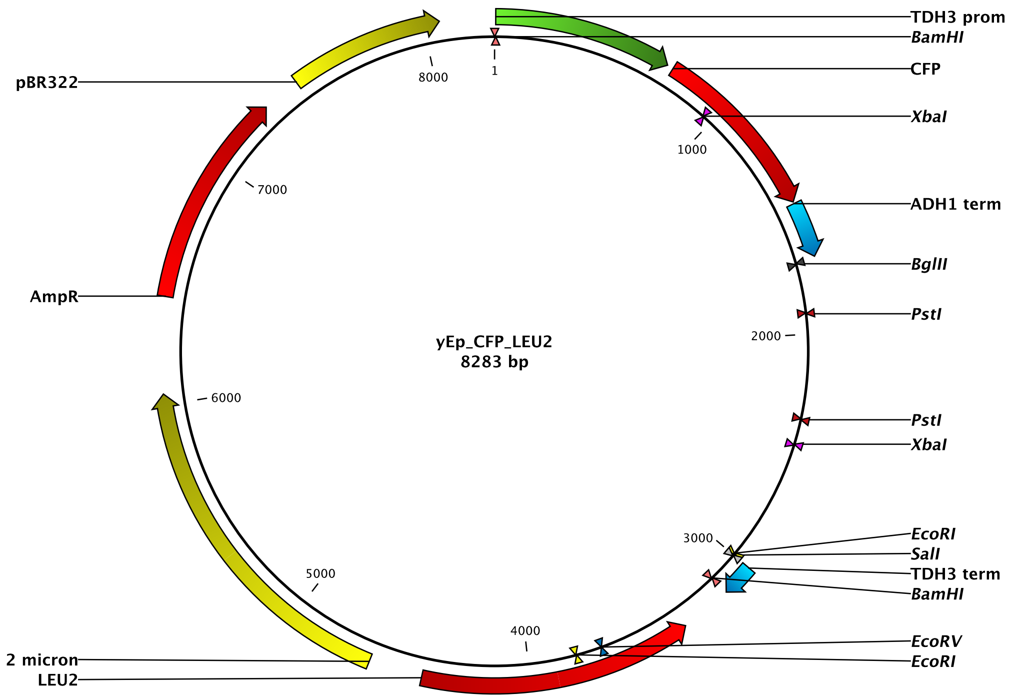

Supplementary figure S6. *Plasmid map of yEp_CFP_URA3.*

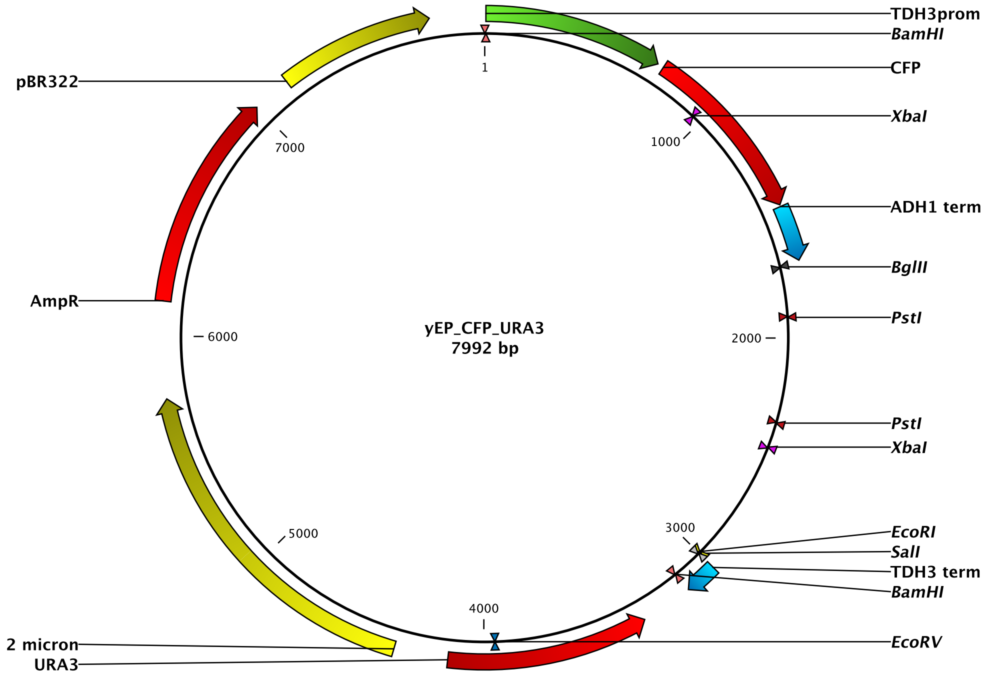

Supplementary figure S7. *Plasmid map of yEp_Cherry_HIS3.*

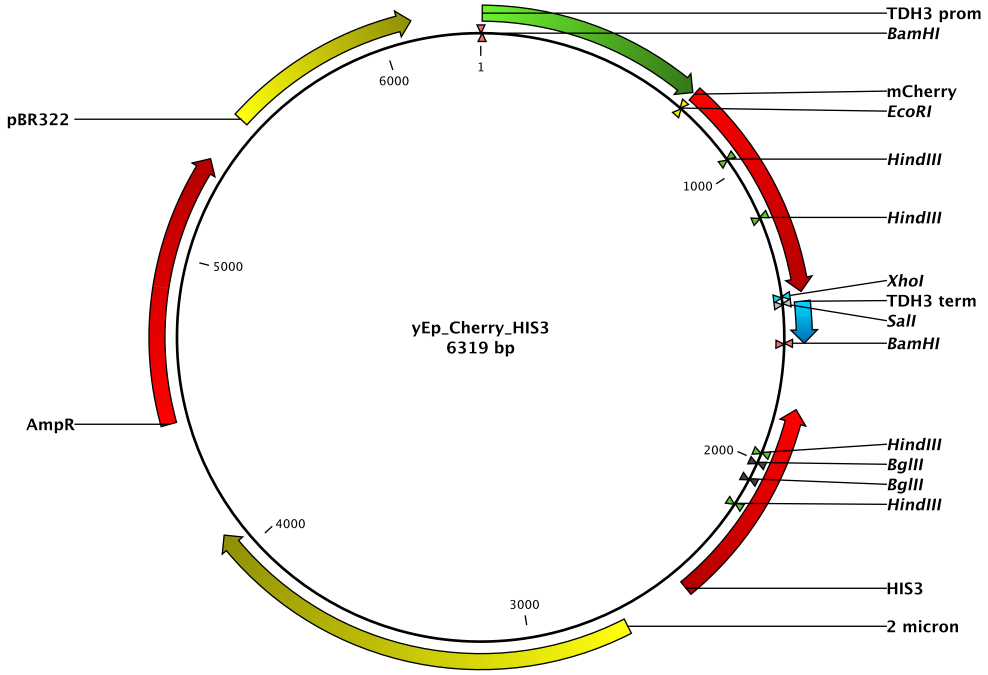

Supplementary figure S8. *Plasmid map of yEp_ Cherry_LEU2.*

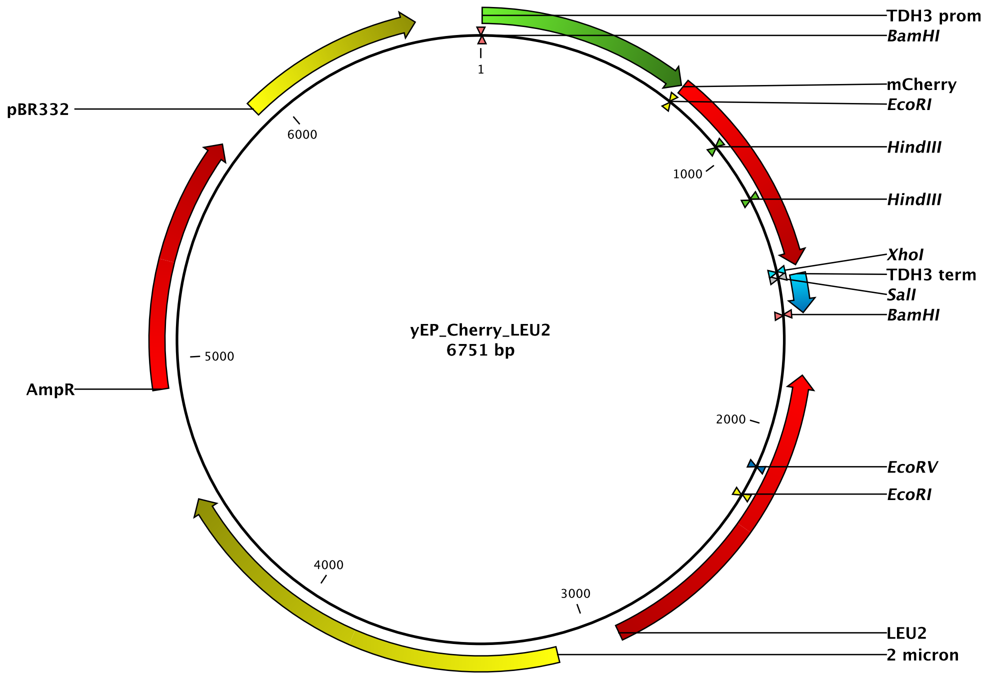

Supplementary figure S9. *Plasmid map of yEpGAP_ Cherry.*

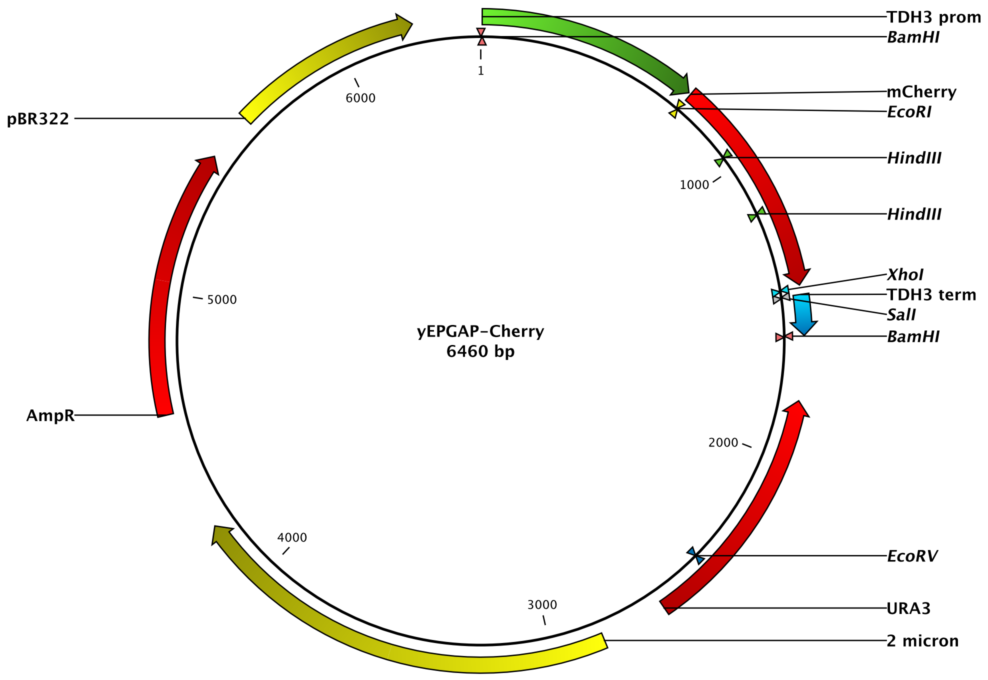

Supplementary figure S10. Plasmid map of yEp_Sapphire_HIS3.

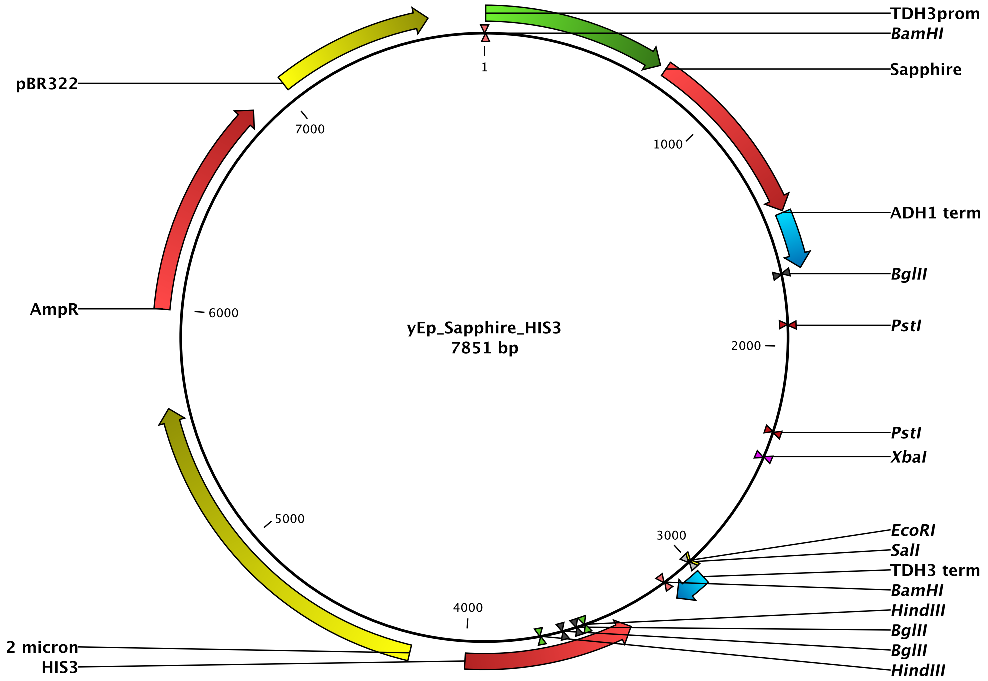

Supplementary figure S11. Plasmid map of yEp_ Sapphire _LEU2.

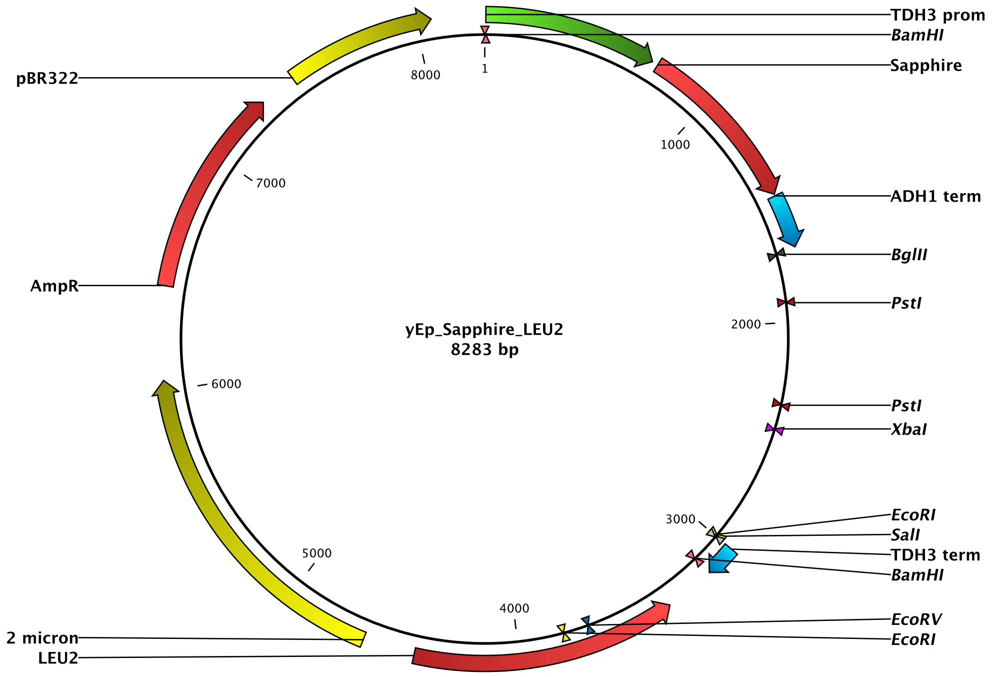

Supplementary figure S12. *Plasmid map of yEp_ Sapphire _URA3.*

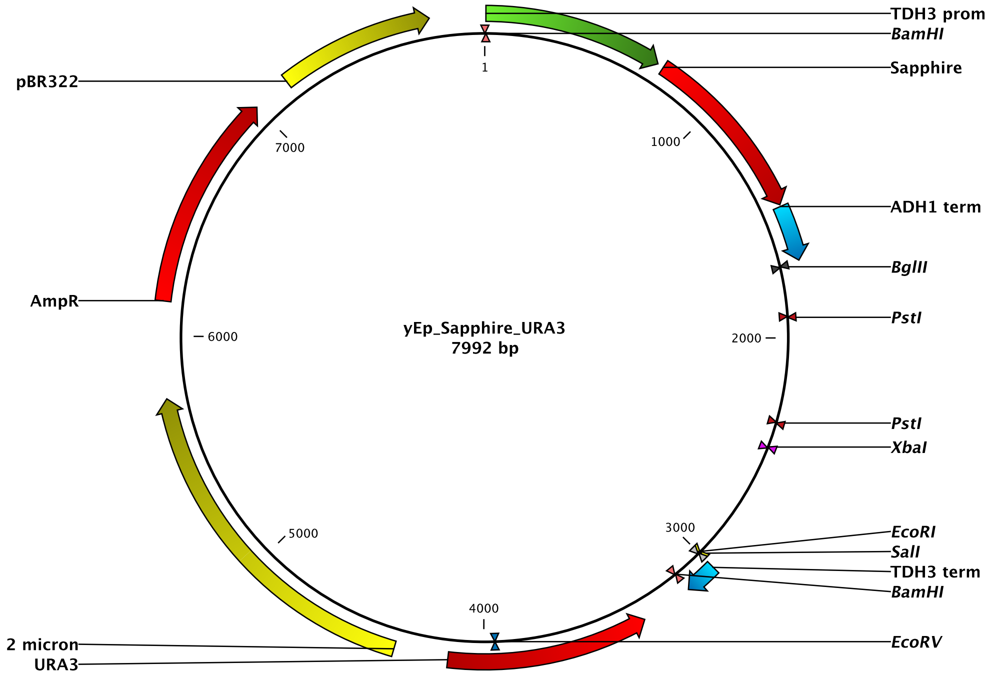

Supplementary figure S13. *Plasmid map of yEp_Venus_HIS3.*

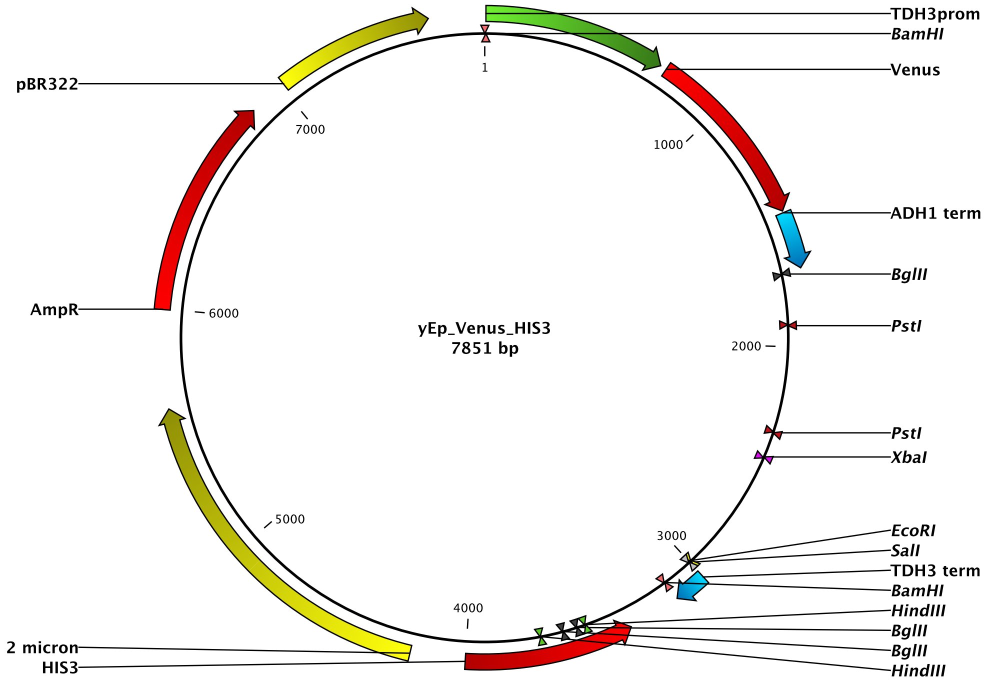

Supplementary figure S14. _Plasmid map of yEp_Venus_LEU2._

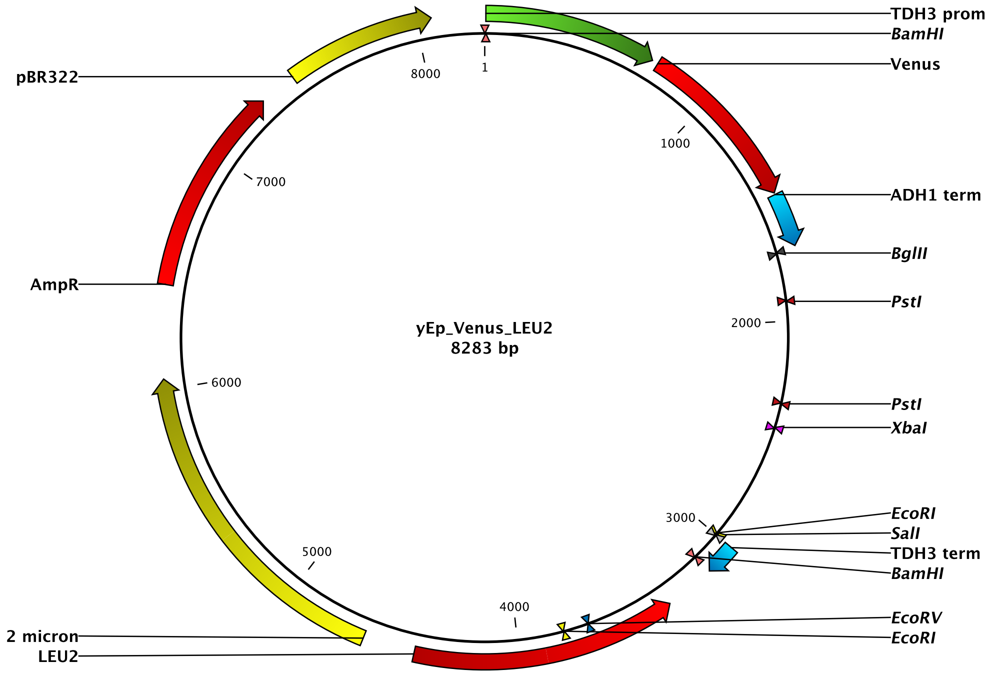

Supplementary figure S15. _Plasmid map of yEp_Venus_URA3._

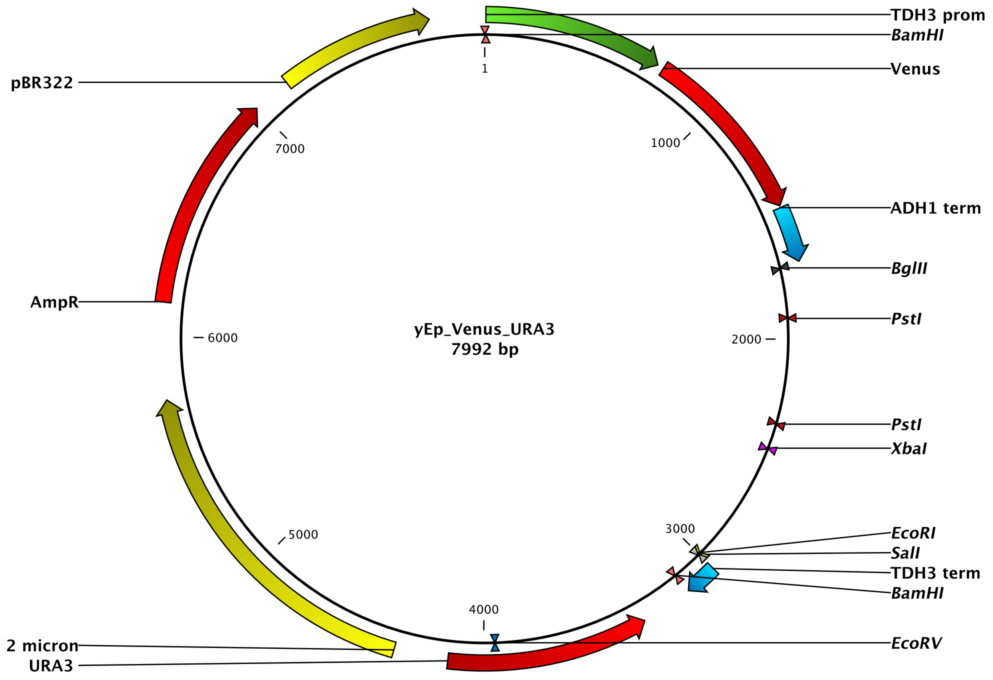

Supplementary plasmid sequences. *Sequences of fluorescent plasmids described in this work.*

```
LOCUS       YEP_VENUS_URA3      7992 BP    DNA     CIRCULAR UNA 10-JAN-2011
DEFINITION
FEATURES             LOCATION/QUALIFIERS
     PROMOTER        6..719
                     /LABEL="TDH3 PROM"
     CDS             745..1461
                     /LABEL=VENUS
     TERMINATOR      1469..1691
                     /LABEL="ADH1 TERM"
     TERMINATOR      3000..3130
                     /LABEL="TDH3 TERM"
     CDS             COMPLEMENT(3339..4142)
                     /LABEL=URA3
     REP_ORIGIN      4359..5753
                     /LABEL="2 MICRON"
     CDS             6140..7000
                     /LABEL=AMPR
     REP_ORIGIN      7155..7774
                     /LABEL=PBR322
ORIGIN
        1 GGATCCGTTA GAATCATTTT GAATAAAAAA CACGCTTTTT CAGTTCGAGT TTATCATTAT
       61 CAATACTGCC ATTTCAAAGA ATACGTAAAT AATTAATAGT AGTGATTTTC CTAACTTTAT
      121 TTAGTCAAAA AATTAGCCTT TTAATTCTGC TGTAACCCGT ACATGCCCAA AATAGGGGGC
      181 GGGTTACACA GAATATATAA CATCGTAGGT GTCTGGGTGA ACAGTTTATT CCTGGCATCC
      241 ACTAAATATA ATGGAGCCCG CTTTTTAAGC TGGCATCCAG AAAAAAAAAG AATCCCAGCA
      301 CCAAAATATT GTTTTCTTCA CCAACCATCA GTTCATAGGT CCATTCTCTT AGCGCAACTA
      361 CAGAGAACAG GGGCACAAAC AGGCAAAAAA CGGGCACAAC CTCAATGGAG TGATGCAACC
      421 TGCCTGGAGT AAATGATGAC ACAAGGCAAT TGACCCACGC ATGTATCTAT CTCATTTTCT
      481 TACACCTTCT ATTACCTTCT GCTCTCTCTG ATTTGGAAAA AGCTGAAAAA AAAGGTTGAA
      541 ACCAGTTCCC TGAAATTATT CCCCTACTTG ACTAATAAGT ATATAAAGAC GGTAGGTATT
      601 GATTGTAATT CTGTAAATCT ATTTCTTAAA CTTCTTAAAT TCTACTTTTA TAGTTAGTCT
      661 TTTTTTTAGT TTTAAAACAC CAAGAACTTA GTTTCGAATA AACACACATA AACAAACAAA
      721 GGTGACGGTG CTGGTTTAAT TAACATGTCT AAAGGTGAAG AATTATTCAC TGGTGTTGTC
      781 CCAATTTTGG TTGAATTAGA TGGTGATGTT AATGGTCACA AATTTTCTGT CTCCGGTGAA
      841 GGTGAAGGTG ATGCTACTTA CGGTAAATTG ACCTTAAAAT TGATTTGTAC TACTGGTAAA
      901 TTGCCAGTTC CATGGCCAAC CTTAGTCACT ACTTTAGGTT ATGGTTTGCA ATGTTTTGCT
      961 AGATACCCAG ATCATATGAA ACAACATGAC TTTTTCAAGT CTGCCATGCC AGAAGGTTAT
     1021 GTTCAAGAAA GAACTATTTT TTTCAAAGAT GACGGTAACT ACAAGACCAG AGCTGAAGTC
     1081 AAGTTTGAAG GTGATACCTT AGTTAATAGA ATCGAATTAA AAGGTATTGA TTTTAAAGAA
     1141 GATGGTAACA TTTTAGGTCA CAAATTGGAA TACAACTATA ACTCTCACAA TGTTTACATC
     1201 ACTGCTGACA AACAAAAGAA TGGTATCAAA GCTAACTTCA AAATTAGACA CAACATTGAA
     1261 GATGGTGGTG TTCAATTAGC TGACCATTAT CAACAAAATA CTCCAATTGG TGATGGTCCA
     1321 GTCTTGTTAC CAGACAACCA TTACTTATCC TATCAATCTG CCTTATCCAA AGATCCAAAC
     1381 GAAAAGAGAG ACCACATGGT CTTGTTAGAA TTTGTTACTG CTGCTGGTAT TACCCATGGT
     1441 ATGGATGAAT TGTACAAATA AGGCGCGCCA CTTCTAAATA AGCGAATTTC TTATGATTTA
     1501 TGATTTTTAT TATTAAATAA GTTATAAAAA AAATAAGTGT ATACAAATTT TAAAGTGACT
     1561 CTTAGGTTTT AAAACGAAAA TTCTTATTCT TGAGTAACTC TTTCCTGTAG GTCAGGTTGC
     1621 TTTCTCAGGT ATAGTATGAG GTCGCTCTTA TTGACCACAC CTCTACCGGC AGATCCGCTA
     1681 GGGATAACAG GGTAATATAG ATCTGTTTAG CTTGCCTCGT CCCCGCCGGG TCACCCGGCC
     1741 AGCGACATGG AGGCCCAGAA TACCCTCCTT GACAGTCTTG ACGTGCGCAG CTCAGGGGCA
     1801 TGATGTGACT GTCGCCCGTA CATTTAGCCC ATACATCCCC ATGTATAATC ATTTGCATCC
     1861 ATACATTTTG ATGGCCGCAC GGCGCGAAGC AAAAATTACG GCTCCTCGCT GCAGACCTGC
     1921 GAGCAGGGAA ACGCTCCCCT CACAGACGCG TTGAATTGTC CCCACGCCGC GCCCCTGTAG
     1981 AGAAATATAA AAGGTTAGGA TTTGCCACTG AGGTTCTTCT TTCATATACT TCCTTTTAAA
     2041 ATCTTGCTAG GATACAGTTC TCACATCACA TCCGAACATA AACAACCATG GGTAGGAGGG
     2101 CTTTTGTAGA AAGAAATACG AACGAAACGA AAATCAGCGT TGCCATCGCT TTGGACAAAG
     2161 CTCCCTTACC TGAAGAGTCG AATTTTATTG ATGAACTTAT AACTTCCAAG CATGCAAACC
     2221 AAAAGGGAGA ACAAGTAATC CAAGTAGACA CGGGAATTGG ATTCTTGGAT CACATGTATC
```

2281 ATGCACTGGC TAAACATGCA GGCTGGAGCT TACGACTTTA CTCAAGAGGT GATTTAATCA
2341 TCGATGATCA TCACACTGCA GAAGATACTG CTATTGCACT TGGTATTGCA TTCAAGCAGG
2401 CTATGGGTAA CTTTGCCGGC GTTAAAAGAT TTGGACATGC TTATTGTCCA CTTGACGAAG
2461 CTCTTTCTAG AAGCGTAGTT GACTTGTCGG GACGGCCCTA TGCTGTTATC GATTTGGGAT
2521 TAAAGCGTGA AAAGGTTGGG GAATTGTCCT GTGAAATGAT CCCTCACTTA CTATATTCCT
2581 TTTCGGTAGC AGCTGGAATT ACTTTGCATG TTACCTGCTT ATATGGTAGT AATGACCATC
2641 ATCGTGCTGA AAGCGCTTTT AAATCTCTGG CTGTTGCCAT GCGCGCGGCT ACTAGTCTTA
2701 CTGGAAGTTC TGAAGTCCCA AGCACGAAGG GAGTGTTGTA AAGAGTACTG ACAATAAAAA
2761 GATTCTTGTT TTCAAGAACT TGTCATTTGT ATAGTTTTTT TATATTGTAG TTGTTCTATT
2821 TTAATCAAAT GTTAGCGTGA TTTATATTTT TTTTCGCCTC GACATCATCT GCCCAGATGC
2881 GAAGTTAAGT GCGCAGAAAG TAATATCATG CGTCAATCGT ATGTGAATGC TGGTCGCTAT
2941 ACTGCTGTCG ATTCGATACT AACGCCGCCA TCCAGTTTAA ACGAGCTCGA ATTCATCGAG
3001 TCGACTTGGT TGAACACGTT GCCAAGGCTT AAGTGAATTT ACTTTAAATC TTGCATTTAA
3061 ATAAATTTTC TTTTTATAGC TTTATGACTT AGTTTCAATT TATATACTAT TTTAATGACA
3121 TTTTCGATTC GGATCCCTGG CGTAATAGCG AAGAGGCCCG CACCGATCGC CCTTCCCAAC
3181 AGTTGCGCAG CCTGAATGGC GAATGGCGCC TGATGCGGTA TTTTCTCCTT ACGCATCTGT
3241 GCGGTATTTC ACACCGCATA GGGTAATAAC TGATATAATT AAATTGAAGC TCTAATTTGT
3301 GAGTTTAGTA TACATGCATT TACTTATAAT ACAGTTTTTT AGTTTTGCTG GCCGCATCTT
3361 CTCAAATATG CTTCCCAGCC TGCTTTTCTG TAACGTTCAC CCTCTACCTT AGCATCCCTT
3421 CCCTTTGCAA ATAGTCCTCT TCCAACAATA ATAATGTCAG ATCCTGTAGA GACCACATCA
3481 TCCACGGTTC TATACTGTTG ACCCAATGCG TCTCCCTTGT CATCTAAACC CACACCGGGT
3541 GTCATAATCA ACCAATCGTA ACCTTCATCT CTTCCACCCA TGTCTCTTTG AGCAATAAAG
3601 CCGATAACAA AATCTTTGTC GCTCTTCGCA ATGTCAACAG TACCCTTAGT ATATTCTCCA
3661 GTAGATAGGG AGCCCTTGCA TGACAATTCT GCTAACATCA AAAGGCCTCT AGGTTCCTTT
3721 GTTACTTCTT CTGCCGCCTG CTTCAAACCG CTAACAATAC CTGGGCCCAC CACACCGTGT
3781 GCATTCGTAA TGTCTGCCCA TTCTGCTATT CTGTATACAC CCGCAGAGTA CTGCAATTTG
3841 ACTGTATTAC CAATGTCAGC AAATTTTCTG TCTTCGAAGA GTAAAAAATT GTACTTGGCG
3901 GATAATGCCT TTAGCGGCTT AACTGTGCCC TCCATGGAAA AATCAGTCAA GATATCCACA
3961 TGTGTTTTTA GTAAACAAAT TTTGGGACCT AATGCTTCAA CTAACTCCAG TAATTCCTTG
4021 GTGGTACGAA CATCCAATGA AGCACACAAG TTTGTTTGCT TTTCGTGCAT GATATTAAAT
4081 AGCTTGGCAG CAACAGGACT AGGATGAGTA GCAGCACGTT CCTTATATGT AGCTTTCGAC
4141 ATGATTTATC TTCGTTTCGG TTTTTGTTCT GTGCAGTTGG GTTAAGAATA CTGGGCAATT
4201 TCATGTTTCT TCAACACTAC ATATGCGTAT ATATACCAAT CTAAGTCTGT GCTCCTTCCT
4261 TCGTTCTTCC TTCTGTTCGG AGATTACCGA ATCAAAAAAA TTTCAAAGAA ACCGAAATCA
4321 AAAAAAAGAA TAAAAAAAAA ATGATGAATT GAAAAGCTCT TGTTACCCAT CATTGAATTT
4381 TGAACATCCG AACCTGGGAG TTTTCCCTGA AACAGATAGT ATATTTGAAC CTGTATAATA
4441 ATATATAGTC TAGCGCTTTA CGGAAGACAA TGTATGTATT TCGGTTCCTG GAGAAACTAT
4501 TGCATCTATT GCATAGGTAA TCTTGCACGT CGCATCCCCG GTTCATTTTC TGCGTTTCCA
4561 TCTTGCACTT CAATAGCATA TCTTTGTTAA CGAAGCATCT GTGCTTCATT TTGTAGAACA
4621 AAAATGCAAC GCGAGAGCGC TAATTTTTCA AACAAAGAAT CTGAGCTGCA TTTTTACAGA
4681 ACAGAAATGC AACGCGAAAG CGCTATTTTA CCAACGAAGA ATCTGTGCTT CATTTTTGTA
4741 AAACAAAAAT GCAACGCGAG AGCGCTAATT TTTCAAACAA AGAATCTGAG CTGCATTTTT
4801 ACAGAACAGA AATGCAACGC GAGAGCGCTA TTTTACCAAC AAAGAATCTA TACTTCTTTT
4861 TTGTTCTACA AAAATGCATC CCGAGAGCGC TATTTTTCTA ACAAAGCATC TTAGATTACT
4921 TTTTTTCTCC TTTGTGCGCT CTATAATGCA GTCTCTTGAT AACTTTTTGC ACTGTAGGTC
4981 CGTTAAGGTT AGAAGAAGGC TACTTTGGTG TCTATTTTCT CTTCCATAAA AAAAGCCTGA
5041 CTCCACTTCC CGCGTTTACT GATTACTAGC GAAGCTGCGG GTGCATTTTT TCAAGATAAA
5101 GGCATCCCCG ATTATATTCT ATACCGATGT GGATTGCGCA TACTTTGTGA ACAGAAAGTG
5161 ATAGCGTTGA TGATTCTTCA TTGGTCAGAA AATTATGAAC GGTTTCTTCT ATTTTGTCTC
5221 TATATACTAC GTATAGGAAA TGTTTACATT TTCGTATTGT TTTCGATTCA CTCTATGAAT
5281 AGTTCTTACT ACAATTTTTT TGTCTAAAGA GTAATACTAG AGATAAACAT AAAAAATGTA
5341 GAGGTCGAGT TTAGATGCAA GTTCAAGGAG CGAAAGGTGG ATGGGTAGGT TATATAGGGA
5401 TATAGCACAG AGATATATAG CAAAGAGATA CTTTTGAGCA ATGTTTGTGG AAGCGGTATT
5461 CGCAATATTT TAGTAGCTCG TTACAGTCCG GTGCGTTTTT GGTTTTTTGA AAGTGCGTCT
5521 TCAGAGCGCT TTTGGTTTTC AAAAGCGCTC TGAAGTTCCT ATACTTTCTA GCTAGAGAAT
5581 AGGAACTTCG GAATAGGAAC TTCAAAGCGT TTCCGAAAAC GAGCGCTTCC GAAAATGCAA
5641 CGCGAGCTGC GCACATACAG CTCACTGTTC ACGTCGCACC TATATCTGCG TGTTGCCTGT
5701 ATATATATAT ACATGAGAAG AACGGCATAG TGCGTGTTTA TGCTTAAATG CGTTATGGTG
5761 CACTCTCAGT ACAATCTGCT CTGATGCCGC ATAGTTAAGC CAGCCCCGAC ACCCGCCAAC
5821 ACCCGCTGAC GCGCCCTGAC GGGCTTGTCT GCTCCCGGCA TCCGCTTACA GACAAGCTGT
5881 GACCGTCTCC GGGAGCTGCA TGTGTCAGAG GTTTTCACCG TCATCACCGA AACGCGCGAG
5941 ACGAAAGGGC CTCGTGATAC GCCTATTTTT ATAGGTTAAT GTCATGATAA TAATGGTTTC
6001 TTAGACGTCA GGTGGCACTT TTCGGGGAAA TGTGCGCGGA ACCCCTATTT GTTTATTTTT
6061 CTAAATACAT TCAAATATGT ATCCGCTCAT GAGACAATAA CCCTGATAAA TGCTTCAATA
6121 ATATTGAAAA AGGAAGAGTA TGAGTATTCA ACATTTCCGT GTCGCCCTTA TTCCCTTTTT
6181 TGCGGCATTT TGCCTTCCTG TTTTTGCTCA CCCAGAAACG CTGGTGAAAG TAAAAGATGC
6241 TGAAGATCAG TTGGGTGCAC GAGTGGGTTA CATCGAACTG GATCTCAACA GCGGTAAGAT
6301 CCTTGAGAGT TTTCGCCCCG AAGAACGTTT TCCAATGATG AGCACTTTTA AAGTTCTGCT
6361 ATGTGGCGCG GTATTATCCC GTATTGACGC CGGGCAAGAG CAACTCGGTC GCCGCATACA
6421 CTATTCTCAG AATGACTTGG TTGAGTACTC ACCAGTCACA GAAAAGCATC TTACGGATGG

```
     6481 CATGACAGTA AGAGAATTAT GCAGTGCTGC CATAACCATG AGTGATAACA CTGCGGCCAA
     6541 CTTACTTCTG ACAACGATCG GAGGACCGAA GGAGCTAACC GCTTTTTTGC ACAACATGGG
     6601 GGATCATGTA ACTCGCCTTG ATCGTTGGGA ACCGGAGCTG AATGAAGCCA TACCAAACGA
     6661 CGAGCGTGAC ACCACGATGC CTGTAGCAAT GGCAACAACG TTGCGCAAAC TATTAACTGG
     6721 CGAACTACTT ACTCTAGCTT CCCGGCAACA ATTAATAGAC TGGATGGAGG CGGATAAAGT
     6781 TGCAGGACCA CTTCTGCGCT CGGCCCTTCC GGCTGGCTGG TTTATTGCTG ATAAATCTGG
     6841 AGCCGGTGAG CGTGGGTCTC GCGGTATCAT TGCAGCACTG GGGCCAGATG GTAAGCCCTC
     6901 CCGTATCGTA GTTATCTACA CGACGGGGAG TCAGGCAACT ATGGATGAAC GAAATAGACA
     6961 GATCGCTGAG ATAGGTGCCT CACTGATTAA GCATTGGTAA CTGTCAGACC AAGTTTACTC
     7021 ATATATACTT TAGATTGATT TAAAACTTCA TTTTTAATTT AAAAGGATCT AGGTGAAGAT
     7081 CCTTTTTGAT AATCTCATGA CCAAAATCCC TTAACGTGAG TTTTCGTTCC ACTGAGCGTC
     7141 AGACCCCGTA GAAAAGATCA AAGGATCTTC TTGAGATCCT TTTTTTCTGC GCGTAATCTG
     7201 CTGCTTGCAA ACAAAAAAAC CACCGCTACC AGCGGTGGTT TGTTTGCCGG ATCAAGAGCT
     7261 ACCAACTCTT TTTCCGAAGG TAACTGGCTT CAGCAGAGCG CAGATACCAA ATACTGTCCT
     7321 TCTAGTGTAG CCGTAGTTAG GCCACCACTT CAAGAACTCT GTAGCACCGC CTACATACCT
     7381 CGCTCTGCTA ATCCTGTTAC CAGTGGCTGC TGCCAGTGGC GATAAGTCGT GTCTTACCGG
     7441 GTTGGACTCA AGACGATAGT TACCGGATAA GGCGCAGCGG TCGGGCTGAA CGGGGGGTTC
     7501 GTGCACACAG CCCAGCTTGG AGCGAACGAC CTACACCGAA CTGAGATACC TACAGCGTGA
     7561 GCATTGAGAA AGCGCCACGC TTCCCGAAGG GAGAAAGGCG GACAGGTATC CGGTAAGCGG
     7621 CAGGGTCGGA ACAGGAGAGC GCACGAGGGA GCTTCCAGGG GGAAACGCCT GGTATCTTTA
     7681 TAGTCCTGTC GGGTTTCGCC ACCTCTGACT TGAGCGTCGA TTTTTGTGAT GCTCGTCAGG
     7741 GGGGCGGAGC CTATGGAAAA ACGCCAGCAA CGCGGCCTTT TTACGGTTCC TGGCCTTTTG
     7801 CTGGCCTTTT GCTCACATGT TCTTTCCTGC GTTATCCCCT GATTCTGTGG ATAACCGTAT
     7861 TACCGCCTTT GAGTGAGCTG ATACCGCTCG CCGCAGCCGA ACGACCGAGC GCAGCGAGTC
     7921 AGTGAGCGAG GAAGCGGAAG AGCGCCCAAT ACGCAAACCG CCTCTCCCCG CGCGTTGGCC
     7981 GATTCATTAA TC
//
LOCUS       YEP_CFP_URA3      7992 BP    DNA     CIRCULAR UNA 10-JAN-2011
DEFINITION
FEATURES             LOCATION/QUALIFIERS
     PROMOTER        6..719
                     /LABEL=TDH3PROM
     CDS             745..1461
                     /LABEL=CFP
     TERMINATOR      1469..1691
                     /LABEL="ADH1 TERM"
     TERMINATOR      3000..3130
                     /LABEL="TDH3 TERM"
     CDS             COMPLEMENT(3339..4142)
                     /LABEL=URA3
     REP_ORIGIN      4359..5753
                     /LABEL="2 MICRON"
     CDS             6140..7000
                     /LABEL=AMPR
     REP_ORIGIN      7155..7774
                     /LABEL=PBR322
ORIGIN
        1 GGATCCGTTA GAATCATTTT GAATAAAAAA CACGCTTTTT CAGTTCGAGT TTATCATTAT
       61 CAATACTGCC ATTTCAAAGA ATACGTAAAT AATTAATAGT AGTGATTTTC CTAACTTTAT
      121 TTAGTCAAAA AATTAGCCTT TTAATTCTGC TGTAACCCGT ACATGCCCAA AATAGGGGGC
      181 GGGTTACACA GAATATATAA CATCGTAGGT GTCTGGGTGA ACAGTTTATT CCTGGCATCC
      241 ACTAAATATA ATGGAGCCCG CTTTTTAAGC TGGCATCCAG AAAAAAAAAG AATCCCAGCA
      301 CCAAAATATT GTTTTCTTCA CCAACCATCA GTTCATAGGT CCATTCTCTT AGCGCAACTA
      361 CAGAGAACAG GGGCACAAAC AGGCAAAAAA CGGGCACAAC CTCAATGGAG TGATGCAACC
      421 TGCCTGGAGT AAATGATGAC ACAAGGCAAT TGACCCACGC ATGTATCTAT CTCATTTTCT
      481 TACACCTTCT ATTACCTTCT GCTCTCTCTG ATTTGGAAAA AGCTGAAAAA AAAGGTTGAA
      541 ACCAGTTCCC TGAAATTATT CCCCTACTTG ACTAATAAGT ATATAAAGAC GGTAGGTATT
      601 GATTGTAATT CTGTAAATCT ATTTCTTAAA CTTCTTAAAT TCTACTTTTA TAGTTAGTCT
      661 TTTTTTTAGT TTTAAAACAC CAAGAACTTA GTTTCGAATA AACACACATA AACAAACAAA
      721 GGTGACGGTG CTGGTTTAAT TAACATGTCT AAAGGTGAAG AATTATTCAC TGGTGTTGTC
      781 CCAATTTTGG TTGAATTAGA TGGTGATGTT AATGGTCACA AATTTTCTGT CTCCGGTGAA
      841 GGTGAAGGTG ATGCTACTTA CGGTAAATTG ACCTTAAAAT TTATTTGTAC TACTGGTAAA
      901 TTGCCAGTTC CATGGCCAAC CTTAGTCACT ACTTTAACTT GGGGTGTTCA ATGTTTTTCT
      961 AGATACCCAG ATCATATGAA ACAACATGAC TTTTTCAAGT CTGCCATGCC AGAAGGTTAT
     1021 GTTCAAGAAA GAACTATTTT TTTCAAAGAT GACGGTAACT ACAAGACCAG AGCTGAAGTC
     1081 AAGTTTGAAG GTGATACCTT AGTTAATAGA ATCGAATTAA AAGGTATTGA TTTTAAAGAA
     1141 GATGGTAACA TTTTAGGTCA CAAATTGGAA TACATTTATA ACTCTCACAA TGTTTACATC
     1201 ACTGCTGACA AACAAAAGAA TGGTATCAAA GCTAACTTCA AAATTAGACA CAACATTGAA
     1261 GATGGTTCTG TTCAATTAGC TGACCATTAT CAACAAAATA CTCCAATTGG TGATGGTCCA
     1321 GTCTTGTTAC CAGACAACCA TTACTTATCC ACTCAATCTG CCTTATCCAA AGATCCAAAC
```

1381 GAAAAGAGAG ACCACATGGT CTTGTTAGAA TTTGTTACTG CTGCTGGTAT TACCCATGGT
1441 ATGGATGAAT TGTACAAATA AGGCGCGCCA CTTCTAAATA AGCGAATTTC TTATGATTTA
1501 TGATTTTTAT TATTAAATAA GTTATAAAAA AAATAAGTGT ATACAAATTT TAAAGTGACT
1561 CTTAGGTTTT AAAACGAAAA TTCTTATTCT TGAGTAACTC TTTCCTGTAG GTCAGGTTGC
1621 TTTCTCAGGT ATAGTATGAG GTCGCTCTTA TTGACCACAC CTCTACCGGC AGATCCGCTA
1681 GGGATAACAG GGTAATATAG ATCTGTTTAG CTTGCCTCGT CCCCGCCGGG TCACCCGGCC
1741 AGCGACATGG AGGCCCAGAA TACCCTCCTT GACAGTCTTG ACGTGCGCAG CTCAGGGGCA
1801 TGATGTGACT GTCGCCCGTA CATTTAGCCC ATACATCCCC ATGTATAATC ATTTGCATCC
1861 ATACATTTTG ATGGCCGCAC GGCGCGAAGC AAAAATTACG GCTCCTCGCT GCAGACCTGC
1921 GAGCAGGGAA ACGCTCCCCT CACAGACGCG TTGAATTGTC CCCACGCCGC GCCCCTGTAG
1981 AGAAATATAA AAGGTTAGGA TTTGCCACTG AGGTTCTTCT TTCATATACT TCCTTTTAAA
2041 ATCTTGCTAG GATACAGTTC TCACATCACA TCCGAACATA AACAACCATG GGTAGGAGGG
2101 CTTTTGTAGA AAGAAATACG AACGAAACGA AAATCAGCGT TGCCATCGCT TTGGACAAAG
2161 CTCCCTTACC TGAAGAGTCG AATTTTATTG ATGAACTTAT AACTTCCAAG CATGCAAACC
2221 AAAAGGGAGA ACAAGTAATC CAAGTAGACA CGGGAATTGG ATTCTTGGAT CACATGTATC
2281 ATGCACTGGC TAAACATGCA GGCTGGAGCT TACGACTTTA CTCAAGAGGT GATTTAATCA
2341 TCGATGATCA TCACACTGCA GAAGATACTG CTATTGCACT TGGTATTGCA TTCAAGCAGG
2401 CTATGGGTAA CTTTGCCGGC GTTAAAAGAT TTGGACATGC TTATTGTCCA CTTGACGAAG
2461 CTCTTTCTAG AAGCGTAGTT GACTTGTCGG GACGGCCCTA TGCTGTTATC GATTTGGGAT
2521 TAAAGCGTGA AAAGGTTGGG GAATTGTCCT GTGAAATGAT CCCTCACTTA CTATATTCCT
2581 TTTCGGTAGC AGCTGGAATT ACTTTGCATG TTACCTGCTT ATATGGTAGT AATGACCATC
2641 ATCGTGCTGA AAGCGCTTTT AAATCTCTGG CTGTTGCCAT GCGCGCGGCT ACTAGTCTTA
2701 CTGGAAGTTC TGAAGTCCCA AGCACGAAGG GAGTGTTGTA AAGAGTACTG ACAATAAAAA
2761 GATTCTTGTT TTCAAGAACT TGTCATTTGT ATAGTTTTTT TATATTGTAG TTGTTCTATT
2821 TTAATCAAAT GTTAGCGTGA TTTATATTTT TTTTCGCCTC GACATCATCT GCCCAGATGC
2881 GAAGTTAAGT GCGCAGAAAG TAATATCATG CGTCAATCGT ATGTGAATGC TGGTCGCTAT
2941 ACTGCTGTCG ATTCGATACT AACGCCGCCA TCCAGTTTAA ACGAGCTCGA ATTCATCGAG
3001 TCGACTTGGT TGAACACGTT GCCAAGGCTT AAGTGAATTT ACTTTAAATC TTGCATTTAA
3061 ATAAATTTTC TTTTTATAGC TTTATGACTT AGTTTCAATT TATATACTAT TTTAATGACA
3121 TTTTCGATTC GGATCCCTGG CGTAATAGCG AAGAGGCCCG CACCGATCGC CCTTCCCAAC
3181 AGTTGCGCAG CCTGAATGGC GAATGGCGCC TGATGCGGTA TTTTCTCCTT ACGCATCTGT
3241 GCGGTATTTC ACACCGCATA GGGTAATAAC TGATATAATT AAATTGAAGC TCTAATTTGT
3301 GAGTTTAGTA TACATGCATT TACTTATAAT ACAGTTTTTT AGTTTTGCTG GCCGCATCTT
3361 CTCAAATATG CTTCCCAGCC TGCTTTTCTG TAACGTTCAC CCTCTACCTT AGCATCCCTT
3421 CCCTTTGCAA ATAGTCCTCT TCCAACAATA ATAATGTCAG ATCCTGTAGA GACCACATCA
3481 TCCACGGTTC TATACTGTTG ACCCAATGCG TCTCCCTTGT CATCTAAACC CACACCGGGT
3541 GTCATAATCA ACCAATCGTA ACCTTCATCT CTTCCACCCA TGTCTCTTTG AGCAATAAAG
3601 CCGATAACAA AATCTTTGTC GCTCTTCGCA ATGTCAACAG TACCCTTAGT ATATTCTCCA
3661 GTAGATAGGG AGCCCTTGCA TGACAATTCT GCTAACATCA AAAGGCCTCT AGGTTCCTTT
3721 GTTACTTCTT CTGCCGCCTG CTTCAAACCG CTAACAATAC CTGGGCCCAC CACACCGTGT
3781 GCATTCGTAA TGTCTGCCCA TTCTGCTATT CTGTATACAC CCGCAGAGTA CTGCAATTTG
3841 ACTGTATTAC CAATGTCAGC AAATTTTCTG TCTTCGAAGA GTAAAAAATT GTACTTGGCG
3901 GATAATGCCT TTAGCGGCTT AACTGTGCCC TCCATGGAAA AATCAGTCAA GATATCCACA
3961 TGTGTTTTTA GTAAACAAAT TTTGGGACCT AATGCTTCAA CTAACTCCAG TAATTCCTTG
4021 GTGGTACGAA CATCCAATGA AGCACACAAG TTTGTTTGCT TTTCGTGCAT GATATTAAAT
4081 AGCTTGGCAG CAACAGGACT AGGATGAGTA GCAGCACGTT CCTTATATGT AGCTTTCGAC
4141 ATGATTTATC TTCGTTTCGG TTTTTGTTCT GTGCAGTTGG GTTAAGAATA CTGGGCAATT
4201 TCATGTTTCT TCAACACTAC ATATGCGTAT ATATACCAAT CTAAGTCTGT GCTCCTTCCT
4261 TCGTTCTTCC TTCTGTTCGG AGATTACCGA ATCAAAAAAA TTTCAAAGAA ACCGAAATCA
4321 AAAAAAAGAA TAAAAAAAAA ATGATGAATT GAAAAGCTCT TGTTACCCAT CATTGAATTT
4381 TGAACATCCG AACCTGGGAG TTTTCCCTGA AACAGATAGT ATATTTGAAC CTGTATAATA
4441 ATATATAGTC TAGCGCTTTA CGGAAGACAA TGTATGTATT TCGGTTCCTG GAGAAACTAT
4501 TGCATCTATT GCATAGGTAA TCTTGCACGT CGCATCCCCG GTTCATTTTC TGCGTTTCCA
4561 TCTTGCACTT CAATAGCATA TCTTTGTTAA CGAAGCATCT GTGCTTCATT TTGTAGAACA
4621 AAAATGCAAC GCGAGAGCGC TAATTTTTCA AACAAAGAAT CTGAGCTGCA TTTTTACAGA
4681 ACAGAAATGC AACGCGAAAG CGCTATTTTA CCAACGAAGA ATCTGTGCTT CATTTTTGTA
4741 AAACAAAAAT GCAACGCGAG AGCGCTAATT TTTCAAACAA AGAATCTGAG CTGCATTTTT
4801 ACAGAACAGA AATGCAACGC GAGAGCGCTA TTTTACCAAC AAAGAATCTA TACTTCTTTT
4861 TTGTTCTACA AAAATGCATC CCGAGAGCGC TATTTTTCTA ACAAAGCATC TTAGATTACT
4921 TTTTTTCTCC TTTGTGCGCT CTATAATGCA GTCTCTTGAT AACTTTTTGC ACTGTAGGTC
4981 CGTTAAGGTT AGAAGAAGGC TACTTTGGTG TCTATTTTCT CTTCCATAAA AAAAGCCTGA
5041 CTCCACTTCC CGCGTTTACT GATTACTAGC GAAGCTGCGG GTGCATTTTT TCAAGATAAA
5101 GGCATCCCCG ATTATATTCT ATACCGATGT GGATTGCGCA TACTTTGTGA ACAGAAAGTG
5161 ATAGCGTTGA TGATTCTTCA TTGGTCAGAA AATTATGAAC GGTTTCTTCT ATTTTGTCTC
5221 TATATACTAC GTATAGGAAA TGTTTACATT TTCGTATTGT TTTCGATTCA CTCTATGAAT
5281 AGTTCTTACT ACAATTTTTT TGTCTAAAGA GTAATACTAG AGATAAACAT AAAAAATGTA
5341 GAGGTCGAGT TTAGATGCAA GTTCAAGGAG CGAAAGGTGG ATGGGTAGGT TATATAGGGA
5401 TATAGCACAG AGATATATAG CAAAGAGATA CTTTTGAGCA ATGTTTGTGG AAGCGGTATT
5461 CGCAATATTT TAGTAGCTCG TTACAGTCCG GTGCGTTTTT GGTTTTTTGA AAGTGCGTCT
5521 TCAGAGCGCT TTTGGTTTTC AAAAGCGCTC TGAAGTTCCT ATACTTTCTA GCTAGAGAAT

```
     5581 AGGAACTTCG GAATAGGAAC TTCAAAGCGT TTCCGAAAAC GAGCGCTTCC GAAAATGCAA
     5641 CGCGAGCTGC GCACATACAG CTCACTGTTC ACGTCGCACC TATATCTGCG TGTTGCCTGT
     5701 ATATATATAT ACATGAGAAG AACGGCATAG TGCGTGTTTA TGCTTAAATG CGTTATGGTG
     5761 CACTCTCAGT ACAATCTGCT CTGATGCCGC ATAGTTAAGC CAGCCCCGAC ACCCGCCAAC
     5821 ACCCGCTGAC GCGCCCTGAC GGGCTTGTCT GCTCCCGGCA TCCGCTTACA GACAAGCTGT
     5881 GACCGTCTCC GGGAGCTGCA TGTGTCAGAG GTTTTCACCG TCATCACCGA AACGCGCGAG
     5941 ACGAAAGGGC CTCGTGATAC GCCTATTTTT ATAGGTTAAT GTCATGATAA TAATGGTTTC
     6001 TTAGACGTCA GGTGGCACTT TTCGGGGAAA TGTGCGCGGA ACCCCTATTT GTTTATTTTT
     6061 CTAAATACAT TCAAATATGT ATCCGCTCAT GAGACAATAA CCCTGATAAA TGCTTCAATA
     6121 ATATTGAAAA AGGAAGAGTA TGAGTATTCA ACATTTCCGT GTCGCCCTTA TTCCCTTTTT
     6181 TGCGGCATTT TGCCTTCCTG TTTTTGCTCA CCCAGAAACG CTGGTGAAAG TAAAAGATGC
     6241 TGAAGATCAG TTGGGTGCAC GAGTGGGTTA CATCGAACTG GATCTCAACA GCGGTAAGAT
     6301 CCTTGAGAGT TTTCGCCCCG AAGAACGTTT TCCAATGATG AGCACTTTTA AAGTTCTGCT
     6361 ATGTGGCGCG GTATTATCCC GTATTGACGC CGGGCAAGAG CAACTCGGTC GCCGCATACA
     6421 CTATTCTCAG AATGACTTGG TTGAGTACTC ACCAGTCACA GAAAAGCATC TTACGGATGG
     6481 CATGACAGTA AGAGAATTAT GCAGTGCTGC CATAACCATG AGTGATAACA CTGCGGCCAA
     6541 CTTACTTCTG ACAACGATCG GAGGACCGAA GGAGCTAACC GCTTTTTTGC ACAACATGGG
     6601 GGATCATGTA ACTCGCCTTG ATCGTTGGGA ACCGGAGCTG AATGAAGCCA TACCAAACGA
     6661 CGAGCGTGAC ACCACGATGC CTGTAGCAAT GGCAACAACG TTGCGCAAAC TATTAACTGG
     6721 CGAACTACTT ACTCTAGCTT CCCGGCAACA ATTAATAGAC TGGATGGAGG CGGATAAAGT
     6781 TGCAGGACCA CTTCTGCGCT CGGCCCTTCC GGCTGGCTGG TTTATTGCTG ATAAATCTGG
     6841 AGCCGGTGAG CGTGGGTCTC GCGGTATCAT TGCAGCACTG GGGCCAGATG GTAAGCCCTC
     6901 CCGTATCGTA GTTATCTACA CGACGGGGAG TCAGGCAACT ATGGATGAAC GAAATAGACA
     6961 GATCGCTGAG ATAGGTGCCT CACTGATTAA GCATTGGTAA CTGTCAGACC AAGTTTACTC
     7021 ATATATACTT TAGATTGATT TAAAACTTCA TTTTTAATTT AAAAGGATCT AGGTGAAGAT
     7081 CCTTTTTGAT AATCTCATGA CCAAAATCCC TTAACGTGAG TTTTCGTTCC ACTGAGCGTC
     7141 AGACCCCGTA GAAAAGATCA AAGGATCTTC TTGAGATCCT TTTTTTCTGC GCGTAATCTG
     7201 CTGCTTGCAA ACAAAAAAAC CACCGCTACC AGCGGTGGTT TGTTTGCCGG ATCAAGAGCT
     7261 ACCAACTCTT TTTCCGAAGG TAACTGGCTT CAGCAGAGCG CAGATACCAA ATACTGTCCT
     7321 TCTAGTGTAG CCGTAGTTAG GCCACCACTT CAAGAACTCT GTAGCACCGC CTACATACCT
     7381 CGCTCTGCTA ATCCTGTTAC CAGTGGCTGC TGCCAGTGGC GATAAGTCGT GTCTTACCGG
     7441 GTTGGACTCA AGACGATAGT TACCGGATAA GGCGCAGCGG TCGGGCTGAA CGGGGGGTTC
     7501 GTGCACACAG CCCAGCTTGG AGCGAACGAC CTACACCGAA CTGAGATACC TACAGCGTGA
     7561 GCATTGAGAA AGCGCCACGC TTCCCGAAGG GAGAAAGGCG GACAGGTATC CGGTAAGCGG
     7621 CAGGGTCGGA ACAGGAGAGC GCACGAGGGA GCTTCCAGGG GGAAACGCCT GGTATCTTTA
     7681 TAGTCCTGTC GGGTTTCGCC ACCTCTGACT TGAGCGTCGA TTTTTGTGAT GCTCGTCAGG
     7741 GGGGCGGAGC CTATGGAAAA ACGCCAGCAA CGCGGCCTTT TTACGGTTCC TGGCCTTTTG
     7801 CTGGCCTTTT GCTCACATGT TCTTTCCTGC GTTATCCCCT GATTCTGTGG ATAACCGTAT
     7861 TACCGCCTTT GAGTGAGCTG ATACCGCTCG CCGCAGCCGA ACGACCGAGC GCAGCGAGTC
     7921 AGTGAGCGAG GAAGCGGAAG AGCGCCCAAT ACGCAAACCG CCTCTCCCCG CGCGTTGGCC
     7981 GATTCATTAA TC
//
LOCUS       YEP_SAPPHIRE_URA3      7992 BP    DNA     CIRCULAR UNA 10-JAN-
2011
DEFINITION
FEATURES             LOCATION/QUALIFIERS
     PROMOTER        6..719
                     /LABEL="TDH3 PROM"
     CDS             745..1461
                     /LABEL=SAPPHIRE
     TERMINATOR      1469..1691
                     /LABEL="ADH1 TERM"
     TERMINATOR      3000..3130
                     /LABEL="TDH3 TERM"
     CDS             COMPLEMENT(3339..4142)
                     /LABEL=URA3
     REP_ORIGIN      4359..5753
                     /LABEL="2 MICRON"
     CDS             6140..7000
                     /LABEL=AMPR
     REP_ORIGIN      7155..7774
                     /LABEL=PBR322
ORIGIN
        1 GGATCCGTTA GAATCATTTT GAATAAAAAA CACGCTTTTT CAGTTCGAGT TTATCATTAT
       61 CAATACTGCC ATTTCAAAGA ATACGTAAAT AATTAATAGT AGTGATTTTC CTAACTTTAT
      121 TTAGTCAAAA AATTAGCCTT TTAATTCTGC TGTAACCCGT ACATGCCCAA AATAGGGGGC
      181 GGGTTACACA GAATATATAA CATCGTAGGT GTCTGGGTGA ACAGTTTATT CCTGGCATCC
      241 ACTAAATATA ATGGAGCCCG CTTTTTAAGC TGGCATCCAG AAAAAAAAAG AATCCCAGCA
      301 CCAAAATATT GTTTTCTTCA CCAACCATCA GTTCATAGGT CCATTCTCTT AGCGCAACTA
      361 CAGAGAACAG GGGCACAAAC AGGCAAAAAA CGGGCACAAC CTCAATGGAG TGATGCAACC
```

421 TGCCTGGAGT AAATGATGAC ACAAGGCAAT TGACCCACGC ATGTATCTAT CTCATTTTCT
481 TACACCTTCT ATTACCTTCT GCTCTCTCTG ATTTGGAAAA AGCTGAAAAA AAAGGTTGAA
541 ACCAGTTCCC TGAAATTATT CCCCTACTTG ACTAATAAGT ATATAAAGAC GGTAGGTATT
601 GATTGTAATT CTGTAAATCT ATTTCTTAAA CTTCTTAAAT TCTACTTTTA TAGTTAGTCT
661 TTTTTTTAGT TTTAAAACAC CAAGAACTTA GTTTCGAATA AACACACATA AACAAACAAA
721 GGTGACGGTG CTGGTTTAAT TAACATGTCT AAAGGTGAAG AATTATTCAC TGGTGTTGTC
781 CCAATTTTGG TTGAATTAGA TGGTGATGTT AATGGTCACA AATTTTCTGT CTCCGGTGAA
841 GGTGAAGGTG ATGCTACTTA CGGTAAATTG ACCTTAAAAT TTATTTGTAC TACTGGTAAA
901 TTGCCAGTTC CATGGCCAAC CTTAGTCACT ACTTTTTCTT ATGGTGTTCA ATGTTTTGCT
961 AGATACCCAG ATCATATGAA ACAACATGAC TTTTTCAAGT CTGCCATGCC AGAAGGTTAT
1021 GTTCAAGAAA GAACTATTTT TTTCAAAGAT GACGGTAACT ACAAGACCAG AGCTGAAGTC
1081 AAGTTTGAAG GTGATACCTT AGTTAATAGA ATCGAATTAA AAGGTATTGA TTTTAAAGAA
1141 GATGGTAACA TTTTAGGTCA CAAATTGGAA TACAACTTTA ACTCTCACAA TGTTTACATC
1201 ATGGCTGACA AACAAAAGAA TGGTATCAAA GTTAACTTCA AAATTAGACA CAACATTGAA
1261 GATGGTTCTG TTCAATTAGC TGACCATTAT CAACAAAATA CTCCAATTGG TGATGGTCCA
1321 GTCTTGTTAC CAGACAACCA TTACTTATCC ATTCAATCTG CCTTATCCAA AGATCCAAAC
1381 GAAAAGAGAG ACCACATGGT CTTGTTAGAA TTTGTTACTG CTGCTGGTAT TACCCATGGT
1441 ATGGATGAAT TGTACAAATA AGGCGCGCCA CTTCTAAATA AGCGAATTTC TTATGATTTA
1501 TGATTTTTAT TATTAAATAA GTTATAAAAA AAATAAGTGT ATACAAATTT TAAAGTGACT
1561 CTTAGGTTTT AAAACGAAAA TTCTTATTCT TGAGTAACTC TTTCCTGTAG GTCAGGTTGC
1621 TTTCTCAGGT ATAGTATGAG GTCGCTCTTA TTGACCACAC CTCTACCGGC AGATCCGCTA
1681 GGGATAACAG GGTAATATAG ATCTGTTTAG CTTGCCTCGT CCCCGCCGGG TCACCCGGCC
1741 AGCGACATGG AGGCCCAGAA TACCCTCCTT GACAGTCTTG ACGTGCGCAG CTCAGGGGCA
1801 TGATGTGACT GTCGCCCGTA CATTTAGCCC ATACATCCCC ATGTATAATC ATTTGCATCC
1861 ATACATTTTG ATGGCCGCAC GGCGCGAAGC AAAAATTACG GCTCCTCGCT GCAGACCTGC
1921 GAGCAGGGAA ACGCTCCCCT CACAGACGCG TTGAATTGTC CCCACGCCGC GCCCCTGTAG
1981 AGAAATATAA AAGGTTAGGA TTTGCCACTG AGGTTCTTCT TTCATATACT TCCTTTTAAA
2041 ATCTTGCTAG GATACAGTTC TCACATCACA TCCGAACATA AACAACCATG GGTAGGAGGG
2101 CTTTTGTAGA AAGAAATACG AACGAAACGA AAATCAGCGT TGCCATCGCT TTGGACAAAG
2161 CTCCCTTACC TGAAGAGTCG AATTTTATTG ATGAACTTAT AACTTCCAAG CATGCAAACC
2221 AAAAGGGAGA ACAAGTAATC CAAGTAGACA CGGGAATTGG ATTCTTGGAT CACATGTATC
2281 ATGCACTGGC TAAACATGCA GGCTGGAGCT TACGACTTTA CTCAAGAGGT GATTTAATCA
2341 TCGATGATCA TCACACTGCA GAAGATACTG CTATTGCACT TGGTATTGCA TTCAAGCAGG
2401 CTATGGGTAA CTTTGCCGGC GTTAAAAGAT TTGGACATGC TTATTGTCCA CTTGACGAAG
2461 CTCTTTCTAG AAGCGTAGTT GACTTGTCGG GACGGCCCTA TGCTGTTATC GATTTGGGAT
2521 TAAAGCGTGA AAAGGTTGGG GAATTGTCCT GTGAAATGAT CCCTCACTTA CTATATTCCT
2581 TTTCGGTAGC AGCTGGAATT ACTTTGCATG TTACCTGCTT ATATGGTAGT AATGACCATC
2641 ATCGTGCTGA AAGCGCTTTT AAATCTCTGG CTGTTGCCAT GCGCGCGGCT ACTAGTCTTA
2701 CTGGAAGTTC TGAAGTCCCA AGCACGAAGG GAGTGTTGTA AAGAGTACTG ACAATAAAAA
2761 GATTCTTGTT TTCAAGAACT TGTCATTTGT ATAGTTTTTT TATATTGTAG TTGTTCTATT
2821 TTAATCAAAT GTTAGCGTGA TTTATATTTT TTTTCGCCTC GACATCATCT GCCCAGATGC
2881 GAAGTTAAGT GCGCAGAAAG TAATATCATG CGTCAATCGT ATGTGAATGC TGGTCGCTAT
2941 ACTGCTGTCG ATTCGATACT AACGCCGCCA TCCAGTTTAA ACGAGCTCGA ATTCATCGAG
3001 TCGACTTGGT TGAACACGTT GCCAAGGCTT AAGTGAATTT ACTTTAAATC TTGCATTTAA
3061 ATAAATTTTC TTTTTATAGC TTTATGACTT AGTTTCAATT TATATACTAT TTTAATGACA
3121 TTTTCGATTC GGATCCCTGG CGTAATAGCG AAGAGGCCCG CACCGATCGC CCTTCCCAAC
3181 AGTTGCGCAG CCTGAATGGC GAATGGCGCC TGATGCGGTA TTTTCTCCTT ACGCATCTGT
3241 GCGGTATTTC ACACCGCATA GGGTAATAAC TGATATAATT AAATTGAAGC TCTAATTTGT
3301 GAGTTTAGTA TACATGCATT TACTTATAAT ACAGTTTTTT AGTTTTGCTG GCCGCATCTT
3361 CTCAAATATG CTTCCCAGCC TGCTTTTCTG TAACGTTCAC CCTCTACCTT AGCATCCCTT
3421 CCCTTTGCAA ATAGTCCTCT TCCAACAATA ATAATGTCAG ATCCTGTAGA GACCACATCA
3481 TCCACGGTTC TATACTGTTG ACCCAATGCG TCTCCCTTGT CATCTAAACC CACACCGGGT
3541 GTCATAATCA ACCAATCGTA ACCTTCATCT CTTCCACCCA TGTCTCTTTG AGCAATAAAG
3601 CCGATAACAA AATCTTTGTC GCTCTTCGCA ATGTCAACAG TACCCTTAGT ATATTCTCCA
3661 GTAGATAGGG AGCCCTTGCA TGACAATTCT GCTAACATCA AAAGGCCTCT AGGTTCCTTT
3721 GTTACTTCTT CTGCCGCCTG CTTCAAACCG CTAACAATAC CTGGGCCCAC CACACCGTGT
3781 GCATTCGTAA TGTCTGCCCA TTCTGCTATT CTGTATACAC CCGCAGAGTA CTGCAATTTG
3841 ACTGTATTAC CAATGTCAGC AAATTTTCTG TCTTCGAAGA GTAAAAAATT GTACTTGGCG
3901 GATAATGCCT TTAGCGGCTT AACTGTGCCC TCCATGGAAA AATCAGTCAA GATATCCACA
3961 TGTGTTTTTA GTAAACAAAT TTTGGGACCT AATGCTTCAA CTAACTCCAG TAATTCCTTG
4021 GTGGTACGAA CATCCAATGA AGCACACAAG TTTGTTTGCT TTTCGTGCAT GATATTAAAT
4081 AGCTTGGCAG CAACAGGACT AGGATGAGTA GCAGCACGTT CCTTATATGT AGCTTTCGAC
4141 ATGATTTATC TTCGTTTCGG TTTTTGTTCT GTGCAGTTGG GTTAAGAATA CTGGGCAATT
4201 TCATGTTTCT TCAACACTAC ATATGCGTAT ATATACCAAT CTAAGTCTGT GCTCCTTCCT
4261 TCGTTCTTCC TTCTGTTCGG AGATTACCGA ATCAAAAAAA TTTCAAAGAA ACCGAAATCA
4321 AAAAAAAGAA TAAAAAAAAA ATGATGAATT GAAAAGCTCT TGTTACCCAT CATTGAATTT
4381 TGAACATCCG AACCTGGGAG TTTTCCCTGA AACAGATAGT ATATTTGAAC CTGTATAATA
4441 ATATATAGTC TAGCGCTTTA CGGAAGACAA TGTATGTATT TCGGTTCCTG GAGAAACTAT
4501 TGCATCTATT GCATAGGTAA TCTTGCACGT CGCATCCCCG GTTCATTTTC TGCGTTTCCA
4561 TCTTGCACTT CAATAGCATA TCTTTGTTAA CGAAGCATCT GTGCTTCATT TTGTAGAACA

```
     4621 AAAATGCAAC GCGAGAGCGC TAATTTTTCA AACAAAGAAT CTGAGCTGCA TTTTTACAGA
     4681 ACAGAAATGC AACGCGAAAG CGCTATTTTA CCAACGAAGA ATCTGTGCTT CATTTTTGTA
     4741 AAACAAAAAT GCAACGCGAG AGCGCTAATT TTTCAAACAA AGAATCTGAG CTGCATTTTT
     4801 ACAGAACAGA AATGCAACGC GAGAGCGCTA TTTTACCAAC AAAGAATCTA TACTTCTTTT
     4861 TTGTTCTACA AAAATGCATC CCGAGAGCGC TATTTTTCTA ACAAAGCATC TTAGATTACT
     4921 TTTTTTCTCC TTTGTGCGCT CTATAATGCA GTCTCTTGAT AACTTTTTGC ACTGTAGGTC
     4981 CGTTAAGGTT AGAAGAAGGC TACTTTGGTG TCTATTTTCT CTTCCATAAA AAAAGCCTGA
     5041 CTCCACTTCC CGCGTTTACT GATTACTAGC GAAGCTGCGG GTGCATTTTT TCAAGATAAA
     5101 GGCATCCCCG ATTATATTCT ATACCGATGT GGATTGCGCA TACTTTGTGA ACAGAAAGTG
     5161 ATAGCGTTGA TGATTCTTCA TTGGTCAGAA AATTATGAAC GGTTTCTTCT ATTTTGTCTC
     5221 TATATACTAC GTATAGGAAA TGTTTACATT TTCGTATTGT TTTCGATTCA CTCTATGAAT
     5281 AGTTCTTACT ACAATTTTTT TGTCTAAAGA GTAATACTAG AGATAAACAT AAAAAATGTA
     5341 GAGGTCGAGT TTAGATGCAA GTTCAAGGAG CGAAAGGTGG ATGGGTAGGT TATATAGGGA
     5401 TATAGCACAG AGATATATAG CAAAGAGATA CTTTTGAGCA ATGTTTGTGG AAGCGGTATT
     5461 CGCAATATTT TAGTAGCTCG TTACAGTCCG GTGCGTTTTT GGTTTTTTGA AAGTGCGTCT
     5521 TCAGAGCGCT TTTGGTTTTC AAAAGCGCTC TGAAGTTCCT ATACTTTCTA GCTAGAGAAT
     5581 AGGAACTTCG GAATAGGAAC TTCAAAGCGT TTCCGAAAAC GAGCGCTTCC GAAAATGCAA
     5641 CGCGAGCTGC GCACATACAG CTCACTGTTC ACGTCGCACC TATATCTGCG TGTTGCCTGT
     5701 ATATATATAT ACATGAGAAG AACGGCATAG TGCGTGTTTA TGCTTAAATG CGTTATGGTG
     5761 CACTCTCAGT ACAATCTGCT CTGATGCCGC ATAGTTAAGC CAGCCCCGAC ACCCGCCAAC
     5821 ACCCGCTGAC GCGCCCTGAC GGGCTTGTCT GCTCCCGGCA TCCGCTTACA GACAAGCTGT
     5881 GACCGTCTCC GGGAGCTGCA TGTGTCAGAG GTTTTCACCG TCATCACCGA AACGCGCGAG
     5941 ACGAAAGGGC CTCGTGATAC GCCTATTTTT ATAGGTTAAT GTCATGATAA TAATGGTTTC
     6001 TTAGACGTCA GGTGGCACTT TTCGGGGAAA TGTGCGCGGA ACCCCTATTT GTTTATTTTT
     6061 CTAAATACAT TCAAATATGT ATCCGCTCAT GAGACAATAA CCCTGATAAA TGCTTCAATA
     6121 ATATTGAAAA AGGAAGAGTA TGAGTATTCA ACATTTCCGT GTCGCCCTTA TTCCCTTTTT
     6181 TGCGGCATTT TGCCTTCCTG TTTTTGCTCA CCCAGAAACG CTGGTGAAAG TAAAAGATGC
     6241 TGAAGATCAG TTGGGTGCAC GAGTGGGTTA CATCGAACTG GATCTCAACA GCGGTAAGAT
     6301 CCTTGAGAGT TTTCGCCCCG AAGAACGTTT TCCAATGATG AGCACTTTTA AAGTTCTGCT
     6361 ATGTGGCGCG GTATTATCCC GTATTGACGC CGGGCAAGAG CAACTCGGTC GCCGCATACA
     6421 CTATTCTCAG AATGACTTGG TTGAGTACTC ACCAGTCACA GAAAAGCATC TTACGGATGG
     6481 CATGACAGTA AGAGAATTAT GCAGTGCTGC CATAACCATG AGTGATAACA CTGCGGCCAA
     6541 CTTACTTCTG ACAACGATCG GAGGACCGAA GGAGCTAACC GCTTTTTTGC ACAACATGGG
     6601 GGATCATGTA ACTCGCCTTG ATCGTTGGGA ACCGGAGCTG AATGAAGCCA TACCAAACGA
     6661 CGAGCGTGAC ACCACGATGC CTGTAGCAAT GGCAACAACG TTGCGCAAAC TATTAACTGG
     6721 CGAACTACTT ACTCTAGCTT CCCGGCAACA ATTAATAGAC TGGATGGAGG CGGATAAAGT
     6781 TGCAGGACCA CTTCTGCGCT CGGCCCTTCC GGCTGGCTGG TTTATTGCTG ATAAATCTGG
     6841 AGCCGGTGAG CGTGGGTCTC GCGGTATCAT TGCAGCACTG GGGCCAGATG GTAAGCCCTC
     6901 CCGTATCGTA GTTATCTACA CGACGGGGAG TCAGGCAACT ATGGATGAAC GAAATAGACA
     6961 GATCGCTGAG ATAGGTGCCT CACTGATTAA GCATTGGTAA CTGTCAGACC AAGTTTACTC
     7021 ATATATACTT TAGATTGATT TAAAACTTCA TTTTTAATTT AAAAGGATCT AGGTGAAGAT
     7081 CCTTTTTGAT AATCTCATGA CCAAAATCCC TTAACGTGAG TTTTCGTTCC ACTGAGCGTC
     7141 AGACCCCGTA GAAAAGATCA AAGGATCTTC TTGAGATCCT TTTTTTCTGC GCGTAATCTG
     7201 CTGCTTGCAA ACAAAAAAAC CACCGCTACC AGCGGTGGTT TGTTTGCCGG ATCAAGAGCT
     7261 ACCAACTCTT TTTCCGAAGG TAACTGGCTT CAGCAGAGCG CAGATACCAA ATACTGTCCT
     7321 TCTAGTGTAG CCGTAGTTAG GCCACCACTT CAAGAACTCT GTAGCACCGC CTACATACCT
     7381 CGCTCTGCTA ATCCTGTTAC CAGTGGCTGC TGCCAGTGGC GATAAGTCGT GTCTTACCGG
     7441 GTTGGACTCA AGACGATAGT TACCGGATAA GGCGCAGCGG TCGGGCTGAA CGGGGGGTTC
     7501 GTGCACACAG CCCAGCTTGG AGCGAACGAC CTACACCGAA CTGAGATACC TACAGCGTGA
     7561 GCATTGAGAA AGCGCCACGC TTCCCGAAGG GAGAAAGGCG GACAGGTATC CGGTAAGCGG
     7621 CAGGGTCGGA ACAGGAGAGC GCACGAGGGA GCTTCCAGGG GGAAACGCCT GGTATCTTTA
     7681 TAGTCCTGTC GGGTTTCGCC ACCTCTGACT TGAGCGTCGA TTTTTGTGAT GCTCGTCAGG
     7741 GGGGCGGAGC CTATGGAAAA ACGCCAGCAA CGCGGCCTTT TTACGGTTCC TGGCCTTTTG
     7801 CTGGCCTTTT GCTCACATGT TCTTTCCTGC GTTATCCCCT GATTCTGTGG ATAACCGTAT
     7861 TACCGCCTTT GAGTGAGCTG ATACCGCTCG CCGCAGCCGA ACGACCGAGC GCAGCGAGTC
     7921 AGTGAGCGAG GAAGCGGAAG AGCGCCCAAT ACGCAAACCG CCTCTCCCCG CGCGTTGGCC
     7981 GATTCATTAA TC
//
LOCUS       YEP_CHERRY_HIS3      6319 BP    DNA     CIRCULAR UNA 10-JAN-2011
DEFINITION
FEATURES             LOCATION/QUALIFIERS
     PROMOTER        6..719
                     /LABEL="TDH3 PROM"
     CDS             727..1437
                     /LABEL=MCHERRY
     TERMINATOR      1468..1598
                     /LABEL="TDH3 TERM"
     CDS             COMPLEMENT(1807..2469)
                     /LABEL=HIS3
     REP_ORIGIN      2686..4080
```

```
                     /LABEL="2 MICRON"
     CDS             4467..5327
                     /LABEL=AMPR
     REP_ORIGIN      5482..6101
                     /LABEL=PBR322
ORIGIN
        1 GGATCCGTTA GAATCATTTT GAATAAAAAA CACGCTTTTT CAGTTCGAGT TTATCATTAT
       61 CAATACTGCC ATTTCAAAGA ATACGTAAAT AATTAATAGT AGTGATTTTC CTAACTTTAT
      121 TTAGTCAAAA AATTAGCCTT TTAATTCTGC TGTAACCCGT ACATGCCCAA AATAGGGGGC
      181 GGGTTACACA GAATATATAA CATCGTAGGT GTCTGGGTGA ACAGTTTATT CCTGGCATCC
      241 ACTAAATATA ATGGAGCCCG CTTTTTAAGC TGGCATCCAG AAAAAAAAAG AATCCCAGCA
      301 CCAAAATATT GTTTTCTTCA CCAACCATCA GTTCATAGGT CCATTCTCTT AGCGCAACTA
      361 CAGAGAACAG GGGCACAAAC AGGCAAAAAA CGGGCACAAC CTCAATGGAG TGATGCAACC
      421 TGCCTGGAGT AAATGATGAC ACAAGGCAAT TGACCCACGC ATGTATCTAT CTCATTTTCT
      481 TACACCTTCT ATTACCTTCT GCTCTCTCTG ATTTGGAAAA AGCTGAAAAA AAAGGTTGAA
      541 ACCAGTTCCC TGAAATTATT CCCCTACTTG ACTAATAAGT ATATAAAGAC GGTAGGTATT
      601 GATTGTAATT CTGTAAATCT ATTTCTTAAA CTTCTTAAAT TCTACTTTTA TAGTTAGTCT
      661 TTTTTTTAGT TTTAAAACAC CAAGAACTTA GTTTCGAATA AACACACATA AACAAACAAA
      721 GAATTCATGG TTTCAAAAGG TGAAGAAGAT AATATGGCTA TTATTAAAGA ATTTATGAGA
      781 TTTAAAGTTC ATATGGAAGG TTCAGTTAAT GGTCATGAAT TTGAAATTGA AGGTGAAGGT
      841 GAAGGTAGAC CATATGAAGG TACTCAAACT GCTAAATTGA AAGTTACTAA AGGTGGTCCA
      901 TTACCATTTG CTTGGGATAT TTTGTCACCA CAATTTATGT ATGGTTCAAA AGCTTATGTT
      961 AAACATCCAG CTGATATTCC AGATTATTTA AAATTGTCAT TTCCAGAAGG TTTTAAATGG
     1021 GAAAGAGTTA TGAATTTTGA AGATGGTGGT GTTGTTACTG TTACTCAAGA TTCATCATTA
     1081 CAAGATGGTG AATTTATTTA TAAAGTTAAA TTGAGAGGTA CTAATTTTCC ATCAGATGGT
     1141 CCAGTTATGC AAAAAAAAAC TATGGGTTGG GAAGCTTCAT CAGAAAGAAT GTATCCAGAA
     1201 GATGGTGCTT TAAAAGGTGA AATTAAACAA AGATTGAAAT TAAAAGATGG TGGTCATTAT
     1261 GATGCTGAAG TTAAAACTAC TTATAAAGCT AAAAAACCAG TTCAATTACC AGGTGCTTAT
     1321 AATGTTAATA TTAAATTGGA TATTACTTCA CATAATGAAG ATTATACTAT TGTTGAACAA
     1381 TATGAAAGAG CTGAAGGTAG ACATTCAACT GGTGGTATGG ATGAATTATA TAAATAAGGT
     1441 ACCGCTCGAG CAGCTGTGAT TGATTGAGTC GACTTGGTTG AACACGTTGC CAAGGCTTAA
     1501 GTGAATTTAC TTTAAATCTT GCATTTAAAT AAATTTTCTT TTTATAGCTT TATGACTTAG
     1561 TTTCAATTTA TATACTATTT TAATGACATT TTCGATTCGG ATCCCTGGCG TAATAGCGAA
     1621 GAGGCCCGCA CCGATCGCCC TTCCCAACAG TTGCGCAGCC TGAATGGCGA ATGGCGCCTG
     1681 ATGCGGTATT TTCTCCTTAC GCATCTGTGC GGTATTTCAC ACCGCATAGG GTAATAACTG
     1741 ATATAATTAA ATTGAAGCTC TAATTTGTGA GTTTAGTATA CATGCATTTA CTTATAATAC
     1801 AGTTTTCTAC ATAAGAACAC CTTTGGTGGA GGGAACATCG TTGGTACCAT TGGGCGAGGT
     1861 GGCTTCTCTT ATGGCAACCG CAAGAGCCTT GAACGCACTC TCACTACGGT GATGATCATT
     1921 CTTGCCTCGC AGACAATCAA CGTGGAGGGT AATTCTGCTA GCCTCTGCAA AGCTTTCAAG
     1981 AAAATGCGGG ATCATCTCGC AAGAGAGATC TCCTACTTTC TCCCTTTGCA AACCAAGTTC
     2041 GACAACTGCG TACGGCCTGT TCGAAAGATC TACCACCGCT CTGGAAAGTG CCTCATCCAA
     2101 AGGCGCAAAT CCTGATCCAA ACCTTTTTAC TCCACGCACG GCCCCTAGGG CCTCTTTAAA
     2161 AGCTTGACCG AGAGCAATCC CGCAGTCTTC AGTGGTGTGA TGGTCGTCTA TGTGTAAGTC
     2221 ACCAATGCAC TCAACGATTA GCGACCAGCC GGAATGCTTG GCCAGAGCAT GTATCATATG
     2281 GTCCAGAAAC CCTATACCTG TGTGGACGTT AATCACTTGC GATTGTGTGG CCTGTTCTGC
     2341 TACTGCTTCT GCCTCTTTTT CTGGGAAGAT CGAGTGCTCT ATCGCTAGGG GACCACCCTT
     2401 TAAAGAGATC GCAATCTGAA TCTTGGTTTC ATTTGTAATA CGCTTTACTA GGGCTTTCTG
     2461 CTCTGTCATG ATTTATCTTC GTTTCGGTTT TTGTTCTGTG CAGTTGGGTT AAGAATACTG
     2521 GGCAATTTCA TGTTTCTTCA ACACTACATA TGCGTATATA TACCAATCTA AGTCTGTGCT
     2581 CCTTCCTTCG TTCTTCCTTC TGTTCGGAGA TTACCGAATC AAAAAAATTT CAAAGAAACC
     2641 GAAATCAAAA AAAAGAATAA AAAAAAAATG ATGAATTGAA AAGCTCTTGT TACCCATCAT
     2701 TGAATTTTGA ACATCCGAAC CTGGGAGTTT TCCCTGAAAC AGATAGTATA TTTGAACCTG
     2761 TATAATAATA TATAGTCTAG CGCTTTACGG AAGACAATGT ATGTATTTCG GTTCCTGGAG
     2821 AAACTATTGC ATCTATTGCA TAGGTAATCT TGCACGTCGC ATCCCCGGTT CATTTTCTGC
     2881 GTTTCCATCT TGCACTTCAA TAGCATATCT TTGTTAACGA AGCATCTGTG CTTCATTTTG
     2941 TAGAACAAAA ATGCAACGCG AGAGCGCTAA TTTTTCAAAC AAAGAATCTG AGCTGCATTT
     3001 TTACAGAACA GAAATGCAAC GCGAAAGCGC TATTTTACCA ACGAAGAATC TGTGCTTCAT
     3061 TTTTGTAAAA CAAAAATGCA ACGCGAGAGC GCTAATTTTT CAAACAAAGA ATCTGAGCTG
     3121 CATTTTTACA GAACAGAAAT GCAACGCGAG AGCGCTATTT TACCAACAAA GAATCTATAC
     3181 TTCTTTTTTG TTCTACAAAA ATGCATCCCG AGAGCGCTAT TTTTCTAACA AAGCATCTTA
     3241 GATTACTTTT TTTCTCCTTT GTGCGCTCTA TAATGCAGTC TCTTGATAAC TTTTTGCACT
     3301 GTAGGTCCGT TAAGGTTAGA AGAAGGCTAC TTTGGTGTCT ATTTTCTCTT CCATAAAAAA
     3361 AGCCTGACTC CACTTCCCGC GTTTACTGAT TACTAGCGAA GCTGCGGGTG CATTTTTTCA
     3421 AGATAAAGGC ATCCCCGATT ATATTCTATA CCGATGTGGA TTGCGCATAC TTTGTGAACA
     3481 GAAAGTGATA GCGTTGATGA TTCTTCATTG GTCAGAAAAT TATGAACGGT TTCTTCTATT
     3541 TTGTCTCTAT ATACTACGTA TAGGAAATGT TTACATTTTC GTATTGTTTT CGATTCACTC
     3601 TATGAATAGT TCTTACTACA ATTTTTTTGT CTAAAGAGTA ATACTAGAGA TAAACATAAA
     3661 AAATGTAGAG GTCGAGTTTA GATGCAAGTT CAAGGAGCGA AAGGTGGATG GGTAGGTTAT
     3721 ATAGGGATAT AGCACAGAGA TATATAGCAA AGAGATACTT TTGAGCAATG TTTGTGGAAG
     3781 CGGTATTCGC AATATTTTAG TAGCTCGTTA CAGTCCGGTG CGTTTTTGGT TTTTTGAAAG
```

```
     3841 TGCGTCTTCA GAGCGCTTTT GGTTTTCAAA AGCGCTCTGA AGTTCCTATA CTTTCTAGCT
     3901 AGAGAATAGG AACTTCGGAA TAGGAACTTC AAAGCGTTTC CGAAAACGAG CGCTTCCGAA
     3961 AATGCAACGC GAGCTGCGCA CATACAGCTC ACTGTTCACG TCGCACCTAT ATCTGCGTGT
     4021 TGCCTGTATA TATATATACA TGAGAAGAAC GGCATAGTGC GTGTTTATGC TTAAATGCGT
     4081 TATGGTGCAC TCTCAGTACA ATCTGCTCTG ATGCCGCATA GTTAAGCCAG CCCCGACACC
     4141 CGCCAACACC CGCTGACGCG CCCTGACGGG CTTGTCTGCT CCCGGCATCC GCTTACAGAC
     4201 AAGCTGTGAC CGTCTCCGGG AGCTGCATGT GTCAGAGGTT TTCACCGTCA TCACCGAAAC
     4261 GCGCGAGACG AAAGGGCCTC GTGATACGCC TATTTTTATA GGTTAATGTC ATGATAATAA
     4321 TGGTTTCTTA GACGTCAGGT GGCACTTTTC GGGGAAATGT GCGCGGAACC CCTATTTGTT
     4381 TATTTTTCTA AATACATTCA AATATGTATC CGCTCATGAG ACAATAACCC TGATAAATGC
     4441 TTCAATAATA TTGAAAAAGG AAGAGTATGA GTATTCAACA TTTCCGTGTC GCCCTTATTC
     4501 CCTTTTTTGC GGCATTTTGC CTTCCTGTTT TTGCTCACCC AGAAACGCTG GTGAAAGTAA
     4561 AAGATGCTGA AGATCAGTTG GGTGCACGAG TGGGTTACAT CGAACTGGAT CTCAACAGCG
     4621 GTAAGATCCT TGAGAGTTTT CGCCCCGAAG AACGTTTTCC AATGATGAGC ACTTTTAAAG
     4681 TTCTGCTATG TGGCGCGGTA TTATCCCGTA TTGACGCCGG GCAAGAGCAA CTCGGTCGCC
     4741 GCATACACTA TTCTCAGAAT GACTTGGTTG AGTACTCACC AGTCACAGAA AAGCATCTTA
     4801 CGGATGGCAT GACAGTAAGA GAATTATGCA GTGCTGCCAT AACCATGAGT GATAACACTG
     4861 CGGCCAACTT ACTTCTGACA ACGATCGGAG GACCGAAGGA GCTAACCGCT TTTTTGCACA
     4921 ACATGGGGGA TCATGTAACT CGCCTTGATC GTTGGGAACC GGAGCTGAAT GAAGCCATAC
     4981 CAAACGACGA GCGTGACACC ACGATGCCTG TAGCAATGGC AACAACGTTG CGCAAACTAT
     5041 TAACTGGCGA ACTACTTACT CTAGCTTCCC GGCAACAATT AATAGACTGG ATGGAGGCGG
     5101 ATAAAGTTGC AGGACCACTT CTGCGCTCGG CCCTTCCGGC TGGCTGGTTT ATTGCTGATA
     5161 AATCTGGAGC CGGTGAGCGT GGGTCTCGCG GTATCATTGC AGCACTGGGG CCAGATGGTA
     5221 AGCCCTCCCG TATCGTAGTT ATCTACACGA CGGGGAGTCA GGCAACTATG GATGAACGAA
     5281 ATAGACAGAT CGCTGAGATA GGTGCCTCAC TGATTAAGCA TTGGTAACTG TCAGACCAAG
     5341 TTTACTCATA TATACTTTAG ATTGATTTAA AACTTCATTT TTAATTTAAA AGGATCTAGG
     5401 TGAAGATCCT TTTTGATAAT CTCATGACCA AAATCCCTTA ACGTGAGTTT TCGTTCCACT
     5461 GAGCGTCAGA CCCCGTAGAA AAGATCAAAG GATCTTCTTG AGATCCTTTT TTTCTGCGCG
     5521 TAATCTGCTG CTTGCAAACA AAAAAACCAC CGCTACCAGC GGTGGTTTGT TTGCCGGATC
     5581 AAGAGCTACC AACTCTTTTT CCGAAGGTAA CTGGCTTCAG CAGAGCGCAG ATACCAAATA
     5641 CTGTCCTTCT AGTGTAGCCG TAGTTAGGCC ACCACTTCAA GAACTCTGTA GCACCGCCTA
     5701 CATACCTCGC TCTGCTAATC CTGTTACCAG TGGCTGCTGC CAGTGGCGAT AAGTCGTGTC
     5761 TTACCGGGTT GGACTCAAGA CGATAGTTAC CGGATAAGGC GCAGCGGTCG GGCTGAACGG
     5821 GGGGTTCGTG CACACAGCCC AGCTTGGAGC GAACGACCTA CACCGAACTG AGATACCTAC
     5881 AGCGTGAGCA TTGAGAAAGC GCCACGCTTC CCGAAGGGAG AAAGGCGGAC AGGTATCCGG
     5941 TAAGCGGCAG GGTCGGAACA GGAGAGCGCA CGAGGGAGCT TCCAGGGGGA AACGCCTGGT
     6001 ATCTTTATAG TCCTGTCGGG TTTCGCCACC TCTGACTTGA GCGTCGATTT TTGTGATGCT
     6061 CGTCAGGGGG GCGGAGCCTA TGGAAAAACG CCAGCAACGC GGCCTTTTTA CGGTTCCTGG
     6121 CCTTTTGCTG GCCTTTTGCT CACATGTTCT TTCCTGCGTT ATCCCCTGAT TCTGTGGATA
     6181 ACCGTATTAC CGCCTTTGAG TGAGCTGATA CCGCTCGCCG CAGCCGAACG ACCGAGCGCA
     6241 GCGAGTCAGT GAGCGAGGAA GCGGAAGAGC GCCCAATACG CAAACCGCCT CTCCCCGCGC
     6301 GTTGGCCGAT TCATTAATC
//
LOCUS       YEP_VENUS_HIS3      7851 BP    DNA     CIRCULAR UNA 11-JAN-2011
DEFINITION
FEATURES             LOCATION/QUALIFIERS
     PROMOTER        6..719
                     /LABEL=TDH3PROM
     CDS             745..1461
                     /LABEL=VENUS
     TERMINATOR      1469..1691
                     /LABEL="ADH1 TERM"
     TERMINATOR      3000..3130
                     /LABEL="TDH3 TERM"
     CDS             COMPLEMENT(3339..4001)
                     /LABEL=HIS3
     REP_ORIGIN      4218..5612
                     /LABEL="2 MICRON"
     CDS             5999..6859
                     /LABEL=AMPR
     REP_ORIGIN      7014..7633
                     /LABEL=PBR322
ORIGIN
        1 GGATCCGTTA GAATCATTTT GAATAAAAAA CACGCTTTTT CAGTTCGAGT TTATCATTAT
       61 CAATACTGCC ATTTCAAAGA ATACGTAAAT AATTAATAGT AGTGATTTTC CTAACTTTAT
      121 TTAGTCAAAA AATTAGCCTT TTAATTCTGC TGTAACCCGT ACATGCCCAA AATAGGGGGC
      181 GGGTTACACA GAATATATAA CATCGTAGGT GTCTGGGTGA ACAGTTTATT CCTGGCATCC
      241 ACTAAATATA ATGGAGCCCG CTTTTTAAGC TGGCATCCAG AAAAAAAAAG AATCCCAGCA
      301 CCAAAATATT GTTTTCTTCA CCAACCATCA GTTCATAGGT CCATTCTCTT AGCGCAACTA
      361 CAGAGAACAG GGGCACAAAC AGGCAAAAAA CGGGCACAAC CTCAATGGAG TGATGCAACC
```

421 TGCCTGGAGT AAATGATGAC ACAAGGCAAT TGACCCACGC ATGTATCTAT CTCATTTTCT
481 TACACCTTCT ATTACCTTCT GCTCTCTCTG ATTTGGAAAA AGCTGAAAAA AAAGGTTGAA
541 ACCAGTTCCC TGAAATTATT CCCCTACTTG ACTAATAAGT ATATAAAGAC GGTAGGTATT
601 GATTGTAATT CTGTAAATCT ATTTCTTAAA CTTCTTAAAT TCTACTTTTA TAGTTAGTCT
661 TTTTTTTAGT TTTAAAACAC CAAGAACTTA GTTTCGAATA AACACACATA AACAAACAAA
721 GGTGACGGTG CTGGTTTAAT TAACATGTCT AAAGGTGAAG AATTATTCAC TGGTGTTGTC
781 CCAATTTTGG TTGAATTAGA TGGTGATGTT AATGGTCACA AATTTTCTGT CTCCGGTGAA
841 GGTGAAGGTG ATGCTACTTA CGGTAAATTG ACCTTAAAAT TGATTTGTAC TACTGGTAAA
901 TTGCCAGTTC CATGGCCAAC CTTAGTCACT ACTTTAGGTT ATGGTTTGCA ATGTTTTGCT
961 AGATACCCAG ATCATATGAA ACAACATGAC TTTTTCAAGT CTGCCATGCC AGAAGGTTAT
1021 GTTCAAGAAA GAACTATTTT TTTCAAAGAT GACGGTAACT ACAAGACCAG AGCTGAAGTC
1081 AAGTTTGAAG GTGATACCTT AGTTAATAGA ATCGAATTAA AAGGTATTGA TTTTAAAGAA
1141 GATGGTAACA TTTTAGGTCA CAAATTGGAA TACAACTATA ACTCTCACAA TGTTTACATC
1201 ACTGCTGACA AACAAAAGAA TGGTATCAAA GCTAACTTCA AAATTAGACA CAACATTGAA
1261 GATGGTGGTG TTCAATTAGC TGACCATTAT CAACAAAATA CTCCAATTGG TGATGGTCCA
1321 GTCTTGTTAC CAGACAACCA TTACTTATCC TATCAATCTG CCTTATCCAA AGATCCAAAC
1381 GAAAAGAGAG ACCACATGGT CTTGTTAGAA TTTGTTACTG CTGCTGGTAT TACCCATGGT
1441 ATGGATGAAT TGTACAAATA AGGCGCGCCA CTTCTAAATA AGCGAATTTC TTATGATTTA
1501 TGATTTTTAT TATTAAATAA GTTATAAAAA AAATAAGTGT ATACAAATTT TAAAGTGACT
1561 CTTAGGTTTT AAAACGAAAA TTCTTATTCT TGAGTAACTC TTTCCTGTAG GTCAGGTTGC
1621 TTTCTCAGGT ATAGTATGAG GTCGCTCTTA TTGACCACAC CTCTACCGGC AGATCCGCTA
1681 GGGATAACAG GGTAATATAG ATCTGTTTAG CTTGCCTCGT CCCCGCCGGG TCACCCGGCC
1741 AGCGACATGG AGGCCCAGAA TACCCTCCTT GACAGTCTTG ACGTGCGCAG CTCAGGGGCA
1801 TGATGTGACT GTCGCCCGTA CATTTAGCCC ATACATCCCC ATGTATAATC ATTTGCATCC
1861 ATACATTTTG ATGGCCGCAC GGCGCGAAGC AAAAATTACG GCTCCTCGCT GCAGACCTGC
1921 GAGCAGGGAA ACGCTCCCCT CACAGACGCG TTGAATTGTC CCCACGCCGC GCCCCTGTAG
1981 AGAAATATAA AAGGTTAGGA TTTGCCACTG AGGTTCTTCT TTCATATACT TCCTTTTAAA
2041 ATCTTGCTAG GATACAGTTC TCACATCACA TCCGAACATA AACAACCATG GGTAGGAGGG
2101 CTTTTGTAGA AAGAAATACG AACGAAACGA AAATCAGCGT TGCCATCGCT TTGGACAAAG
2161 CTCCCTTACC TGAAGAGTCG AATTTTATTG ATGAACTTAT AACTTCCAAG CATGCAAACC
2221 AAAAGGGAGA ACAAGTAATC CAAGTAGACA CGGGAATTGG ATTCTTGGAT CACATGTATC
2281 ATGCACTGGC TAAACATGCA GGCTGGAGCT TACGACTTTA CTCAAGAGGT GATTTAATCA
2341 TCGATGATCA TCACACTGCA GAAGATACTG CTATTGCACT TGGTATTGCA TTCAAGCAGG
2401 CTATGGGTAA CTTTGCCGGC GTTAAAAGAT TTGGACATGC TTATTGTCCA CTTGACGAAG
2461 CTCTTTCTAG AAGCGTAGTT GACTTGTCGG GACGGCCCTA TGCTGTTATC GATTTGGGAT
2521 TAAAGCGTGA AAAGGTTGGG GAATTGTCCT GTGAAATGAT CCCTCACTTA CTATATTCCT
2581 TTTCGGTAGC AGCTGGAATT ACTTTGCATG TTACCTGCTT ATATGGTAGT AATGACCATC
2641 ATCGTGCTGA AAGCGCTTTT AAATCTCTGG CTGTTGCCAT GCGCGCGGCT ACTAGTCTTA
2701 CTGGAAGTTC TGAAGTCCCA AGCACGAAGG GAGTGTTGTA AAGAGTACTG ACAATAAAAA
2761 GATTCTTGTT TTCAAGAACT TGTCATTTGT ATAGTTTTTT TATATTGTAG TTGTTCTATT
2821 TTAATCAAAT GTTAGCGTGA TTTATATTTT TTTTCGCCTC GACATCATCT GCCCAGATGC
2881 GAAGTTAAGT GCGCAGAAAG TAATATCATG CGTCAATCGT ATGTGAATGC TGGTCGCTAT
2941 ACTGCTGTCG ATTCGATACT AACGCCGCCA TCCAGTTTAA ACGAGCTCGA ATTCATCGAG
3001 TCGACTTGGT TGAACACGTT GCCAAGGCTT AAGTGAATTT ACTTTAAATC TTGCATTTAA
3061 ATAAATTTTC TTTTTATAGC TTTATGACTT AGTTTCAATT TATATACTAT TTTAATGACA
3121 TTTTCGATTC GGATCCCTGG CGTAATAGCG AAGAGGCCCG CACCGATCGC CCTTCCCAAC
3181 AGTTGCGCAG CCTGAATGGC GAATGGCGCC TGATGCGGTA TTTTCTCCTT ACGCATCTGT
3241 GCGGTATTTC ACACCGCATA GGGTAATAAC TGATATAATT AAATTGAAGC TCTAATTTGT
3301 GAGTTTAGTA TACATGCATT TACTTATAAT ACAGTTTTCT ACATAAGAAC ACCTTTGGTG
3361 GAGGGAACAT CGTTGGTACC ATTGGGCGAG GTGGCTTCTC TTATGGCAAC CGCAAGAGCC
3421 TTGAACGCAC TCTCACTACG GTGATGATCA TTCTTGCCTC GCAGACAATC AACGTGGAGG
3481 GTAATTCTGC TAGCCTCTGC AAAGCTTTCA AGAAAATGCG GGATCATCTC GCAAGAGAGA
3541 TCTCCTACTT TCTCCCTTTG CAAACCAAGT TCGACAACTG CGTACGGCCT GTTCGAAAGA
3601 TCTACCACCG CTCTGGAAAG TGCCTCATCC AAAGGCGCAA ATCCTGATCC AAACCTTTTT
3661 ACTCCACGCA CGGCCCCTAG GGCCTCTTTA AAAGCTTGAC CGAGAGCAAT CCCGCAGTCT
3721 TCAGTGGTGT GATGGTCGTC TATGTGTAAG TCACCAATGC ACTCAACGAT TAGCGACCAG
3781 CCGGAATGCT TGGCCAGAGC ATGTATCATA TGGTCCAGAA ACCCTATACC TGTGTGGACG
3841 TTAATCACTT GCGATTGTGT GGCCTGTTCT GCTACTGCTT CTGCCTCTTT TTCTGGGAAG
3901 ATCGAGTGCT CTATCGCTAG GGGACCACCC TTTAAAGAGA TCGCAATCTG AATCTTGGTT
3961 TCATTTGTAA TACGCTTTAC TAGGGCTTTC TGCTCTGTCA TGATTTATCT TCGTTTCGGT
4021 TTTTGTTCTG TGCAGTTGGG TTAAGAATAC TGGGCAATTT CATGTTTCTT CAACACTACA
4081 TATGCGTATA TATACCAATC TAAGTCTGTG CTCCTTCCTT CGTTCTTCCT TCTGTTCGGA
4141 GATTACCGAA TCAAAAAAAT TTCAAAGAAA CCGAAATCAA AAAAAGAAT AAAAAAAAAA
4201 TGATGAATTG AAAAGCTCTT GTTACCCATC ATTGAATTTT GAACATCCGA ACCTGGGAGT
4261 TTTCCCTGAA ACAGATAGTA TATTTGAACC TGTATAATAA TATATAGTCT AGCGCTTTAC
4321 GGAAGACAAT GTATGTATTT CGGTTCCTGG AGAAACTATT GCATCTATTG CATAGGTAAT
4381 CTTGCACGTC GCATCCCCGG TTCATTTTCT GCGTTTCCAT CTTGCACTTC AATAGCATAT
4441 CTTTGTTAAC GAAGCATCTG TGCTTCATTT TGTAGAACAA AAATGCAACG CGAGAGCGCT
4501 AATTTTTCAA ACAAAGAATC TGAGCTGCAT TTTTACAGAA CAGAAATGCA ACGCGAAAGC
4561 GCTATTTTAC CAACGAAGAA TCTGTGCTTC ATTTTTGTAA AACAAAAATG CAACGCGAGA

```
     4621 GCGCTAATTT TTCAAACAAA GAATCTGAGC TGCATTTTTA CAGAACAGAA ATGCAACGCG
     4681 AGAGCGCTAT TTTACCAACA AAGAATCTAT ACTTCTTTTT TGTTCTACAA AAATGCATCC
     4741 CGAGAGCGCT ATTTTTCTAA CAAAGCATCT TAGATTACTT TTTTTCTCCT TTGTGCGCTC
     4801 TATAATGCAG TCTCTTGATA ACTTTTTGCA CTGTAGGTCC GTTAAGGTTA GAAGAAGGCT
     4861 ACTTTGGTGT CTATTTTCTC TTCCATAAAA AAAGCCTGAC TCCACTTCCC GCGTTTACTG
     4921 ATTACTAGCG AAGCTGCGGG TGCATTTTTT CAAGATAAAG GCATCCCCGA TTATATTCTA
     4981 TACCGATGTG GATTGCGCAT ACTTTGTGAA CAGAAAGTGA TAGCGTTGAT GATTCTTCAT
     5041 TGGTCAGAAA ATTATGAACG GTTTCTTCTA TTTTGTCTCT ATATACTACG TATAGGAAAT
     5101 GTTTACATTT TCGTATTGTT TTCGATTCAC TCTATGAATA GTTCTTACTA CAATTTTTTT
     5161 GTCTAAAGAG TAATACTAGA GATAAACATA AAAAATGTAG AGGTCGAGTT TAGATGCAAG
     5221 TTCAAGGAGC GAAAGGTGGA TGGGTAGGTT ATATAGGGAT ATAGCACAGA GATATATAGC
     5281 AAAGAGATAC TTTTGAGCAA TGTTTGTGGA AGCGGTATTC GCAATATTTT AGTAGCTCGT
     5341 TACAGTCCGG TGCGTTTTTG GTTTTTTGAA AGTGCGTCTT CAGAGCGCTT TTGGTTTTCA
     5401 AAAGCGCTCT GAAGTTCCTA TACTTTCTAG CTAGAGAATA GGAACTTCGG AATAGGAACT
     5461 TCAAAGCGTT TCCGAAAACG AGCGCTTCCG AAAATGCAAC GCGAGCTGCG CACATACAGC
     5521 TCACTGTTCA CGTCGCACCT ATATCTGCGT GTTGCCTGTA TATATATATA CATGAGAAGA
     5581 ACGGCATAGT GCGTGTTTAT GCTTAAATGC GTTATGGTGC ACTCTCAGTA CAATCTGCTC
     5641 TGATGCCGCA TAGTTAAGCC AGCCCCGACA CCCGCCAACA CCCGCTGACG CGCCCTGACG
     5701 GGCTTGTCTG CTCCCGGCAT CCGCTTACAG ACAAGCTGTG ACCGTCTCCG GGAGCTGCAT
     5761 GTGTCAGAGG TTTTCACCGT CATCACCGAA ACGCGCGAGA CGAAAGGGCC TCGTGATACG
     5821 CCTATTTTTA TAGGTTAATG TCATGATAAT AATGGTTTCT TAGACGTCAG GTGGCACTTT
     5881 TCGGGGAAAT GTGCGCGGAA CCCCTATTTG TTTATTTTTC TAAATACATT CAAATATGTA
     5941 TCCGCTCATG AGACAATAAC CCTGATAAAT GCTTCAATAA TATTGAAAAA GGAAGAGTAT
     6001 GAGTATTCAA CATTTCCGTG TCGCCCTTAT TCCCTTTTTT GCGGCATTTT GCCTTCCTGT
     6061 TTTTGCTCAC CCAGAAACGC TGGTGAAAGT AAAAGATGCT GAAGATCAGT TGGGTGCACG
     6121 AGTGGGTTAC ATCGAACTGG ATCTCAACAG CGGTAAGATC CTTGAGAGTT TTCGCCCCGA
     6181 AGAACGTTTT CCAATGATGA GCACTTTTAA AGTTCTGCTA TGTGGCGCGG TATTATCCCG
     6241 TATTGACGCC GGGCAAGAGC AACTCGGTCG CCGCATACAC TATTCTCAGA ATGACTTGGT
     6301 TGAGTACTCA CCAGTCACAG AAAAGCATCT TACGGATGGC ATGACAGTAA GAGAATTATG
     6361 CAGTGCTGCC ATAACCATGA GTGATAACAC TGCGGCCAAC TTACTTCTGA CAACGATCGG
     6421 AGGACCGAAG GAGCTAACCG CTTTTTTGCA CAACATGGGG GATCATGTAA CTCGCCTTGA
     6481 TCGTTGGGAA CCGGAGCTGA ATGAAGCCAT ACCAAACGAC GAGCGTGACA CCACGATGCC
     6541 TGTAGCAATG GCAACAACGT TGCGCAAACT ATTAACTGGC GAACTACTTA CTCTAGCTTC
     6601 CCGGCAACAA TTAATAGACT GGATGGAGGC GGATAAAGTT GCAGGACCAC TTCTGCGCTC
     6661 GGCCCTTCCG GCTGGCTGGT TTATTGCTGA TAAATCTGGA GCCGGTGAGC GTGGGTCTCG
     6721 CGGTATCATT GCAGCACTGG GGCCAGATGG TAAGCCCTCC CGTATCGTAG TTATCTACAC
     6781 GACGGGGAGT CAGGCAACTA TGGATGAACG AAATAGACAG ATCGCTGAGA TAGGTGCCTC
     6841 ACTGATTAAG CATTGGTAAC TGTCAGACCA AGTTTACTCA TATATACTTT AGATTGATTT
     6901 AAAACTTCAT TTTTAATTTA AAAGGATCTA GGTGAAGATC CTTTTTGATA ATCTCATGAC
     6961 CAAAATCCCT TAACGTGAGT TTTCGTTCCA CTGAGCGTCA GACCCCGTAG AAAAGATCAA
     7021 AGGATCTTCT TGAGATCCTT TTTTTCTGCG CGTAATCTGC TGCTTGCAAA CAAAAAAACC
     7081 ACCGCTACCA GCGGTGGTTT GTTTGCCGGA TCAAGAGCTA CCAACTCTTT TTCCGAAGGT
     7141 AACTGGCTTC AGCAGAGCGC AGATACCAAA TACTGTCCTT CTAGTGTAGC CGTAGTTAGG
     7201 CCACCACTTC AAGAACTCTG TAGCACCGCC TACATACCTC GCTCTGCTAA TCCTGTTACC
     7261 AGTGGCTGCT GCCAGTGGCG ATAAGTCGTG TCTTACCGGG TTGGACTCAA GACGATAGTT
     7321 ACCGGATAAG GCGCAGCGGT CGGGCTGAAC GGGGGGTTCG TGCACACAGC CCAGCTTGGA
     7381 GCGAACGACC TACACCGAAC TGAGATACCT ACAGCGTGAG CATTGAGAAA GCGCCACGCT
     7441 TCCCGAAGGG AGAAAGGCGG ACAGGTATCC GGTAAGCGGC AGGGTCGGAA CAGGAGAGCG
     7501 CACGAGGGAG CTTCCAGGGG GAAACGCCTG GTATCTTTAT AGTCCTGTCG GGTTTCGCCA
     7561 CCTCTGACTT GAGCGTCGAT TTTTGTGATG CTCGTCAGGG GGGCGGAGCC TATGGAAAAA
     7621 CGCCAGCAAC GCGGCCTTTT TACGGTTCCT GGCCTTTTGC TGGCCTTTTG CTCACATGTT
     7681 CTTTCCTGCG TTATCCCCTG ATTCTGTGGA TAACCGTATT ACCGCCTTTG AGTGAGCTGA
     7741 TACCGCTCGC CGCAGCCGAA CGACCGAGCG CAGCGAGTCA GTGAGCGAGG AAGCGGAAGA
     7801 GCGCCCAATA CGCAAACCGC CTCTCCCCGC GCGTTGGCCG ATTCATTAAT C
//
LOCUS       YEP_CFP_HIS3      7851 BP    DNA     CIRCULAR UNA 10-JAN-2011
DEFINITION
FEATURES             LOCATION/QUALIFIERS
     PROMOTER        6..719
                     /LABEL=TDH3PROM
     CDS             745..1461
                     /LABEL=CFP
     TERMINATOR      1469..1691
                     /LABEL="ADH1 TERM"
     TERMINATOR      3000..3130
                     /LABEL="TDH3 TERM"
     CDS             COMPLEMENT(3339..4001)
                     /LABEL=HIS3
     REP_ORIGIN      4218..5612
                     /LABEL="2 MICRON"
```

```
     CDS             5999..6859
                     /LABEL=AMPR
     REP_ORIGIN      7014..7633
                     /LABEL="PBR322 ORI"
                     /LABEL=PBR322
ORIGIN
        1 GGATCCGTTA GAATCATTTT GAATAAAAAA CACGCTTTTT CAGTTCGAGT TTATCATTAT
       61 CAATACTGCC ATTTCAAAGA ATACGTAAAT AATTAATAGT AGTGATTTTC CTAACTTTAT
      121 TTAGTCAAAA AATTAGCCTT TTAATTCTGC TGTAACCCGT ACATGCCCAA AATAGGGGGC
      181 GGGTTACACA GAATATATAA CATCGTAGGT GTCTGGGTGA ACAGTTTATT CCTGGCATCC
      241 ACTAAATATA ATGGAGCCCG CTTTTTAAGC TGGCATCCAG AAAAAAAAAG AATCCCAGCA
      301 CCAAAATATT GTTTTCTTCA CCAACCATCA GTTCATAGGT CCATTCTCTT AGCGCAACTA
      361 CAGAGAACAG GGGCACAAAC AGGCAAAAAA CGGGCACAAC CTCAATGGAG TGATGCAACC
      421 TGCCTGGAGT AAATGATGAC ACAAGGCAAT TGACCCACGC ATGTATCTAT CTCATTTTCT
      481 TACACCTTCT ATTACCTTCT GCTCTCTCTG ATTTGGAAAA AGCTGAAAAA AAAGGTTGAA
      541 ACCAGTTCCC TGAAATTATT CCCCTACTTG ACTAATAAGT ATATAAAGAC GGTAGGTATT
      601 GATTGTAATT CTGTAAATCT ATTTCTTAAA CTTCTTAAAT TCTACTTTTA TAGTTAGTCT
      661 TTTTTTTAGT TTTAAAACAC CAAGAACTTA GTTTCGAATA AACACACATA AACAAACAAA
      721 GGTGACGGTG CTGGTTTAAT TAACATGTCT AAAGGTGAAG AATTATTCAC TGGTGTTGTC
      781 CCAATTTTGG TTGAATTAGA TGGTGATGTT AATGGTCACA AATTTTCTGT CTCCGGTGAA
      841 GGTGAAGGTG ATGCTACTTA CGGTAAATTG ACCTTAAAAT TTATTTGTAC TACTGGTAAA
      901 TTGCCAGTTC CATGGCCAAC CTTAGTCACT ACTTTAACTT GGGGTGTTCA ATGTTTTTCT
      961 AGATACCCAG ATCATATGAA ACAACATGAC TTTTTCAAGT CTGCCATGCC AGAAGGTTAT
     1021 GTTCAAGAAA GAACTATTTT TTTCAAAGAT GACGGTAACT ACAAGACCAG AGCTGAAGTC
     1081 AAGTTTGAAG GTGATACCTT AGTTAATAGA ATCGAATTAA AAGGTATTGA TTTTAAAGAA
     1141 GATGGTAACA TTTTAGGTCA CAAATTGGAA TACATTTATA ACTCTCACAA TGTTTACATC
     1201 ACTGCTGACA AACAAAAGAA TGGTATCAAA GCTAACTTCA AAATTAGACA CAACATTGAA
     1261 GATGGTTCTG TTCAATTAGC TGACCATTAT CAACAAAATA CTCCAATTGG TGATGGTCCA
     1321 GTCTTGTTAC CAGACAACCA TTACTTATCC ACTCAATCTG CCTTATCCAA AGATCCAAAC
     1381 GAAAAGAGAG ACCACATGGT CTTGTTAGAA TTTGTTACTG CTGCTGGTAT TACCCATGGT
     1441 ATGGATGAAT TGTACAAATA AGGCGCGCCA CTTCTAAATA AGCGAATTTC TTATGATTTA
     1501 TGATTTTTAT TATTAAATAA GTTATAAAAA AAATAAGTGT ATACAAATTT TAAAGTGACT
     1561 CTTAGGTTTT AAAACGAAAA TTCTTATTCT TGAGTAACTC TTTCCTGTAG GTCAGGTTGC
     1621 TTTCTCAGGT ATAGTATGAG GTCGCTCTTA TTGACCACAC CTCTACCGGC AGATCCGCTA
     1681 GGGATAACAG GGTAATATAG ATCTGTTTAG CTTGCCTCGT CCCCGCCGGG TCACCCGGCC
     1741 AGCGACATGG AGGCCCAGAA TACCCTCCTT GACAGTCTTG ACGTGCGCAG CTCAGGGGCA
     1801 TGATGTGACT GTCGCCCGTA CATTTAGCCC ATACATCCCC ATGTATAATC ATTTGCATCC
     1861 ATACATTTTG ATGGCCGCAC GGCGCGAAGC AAAAATTACG GCTCCTCGCT GCAGACCTGC
     1921 GAGCAGGGAA ACGCTCCCCT CACAGACGCG TTGAATTGTC CCCACGCCGC GCCCCTGTAG
     1981 AGAAATATAA AAGGTTAGGA TTTGCCACTG AGGTTCTTCT TTCATATACT TCCTTTTAAA
     2041 ATCTTGCTAG GATACAGTTC TCACATCACA TCCGAACATA AACAACCATG GGTAGGAGGG
     2101 CTTTTGTAGA AAGAAATACG AACGAAACGA AAATCAGCGT TGCCATCGCT TTGGACAAAG
     2161 CTCCCTTACC TGAAGAGTCG AATTTTATTG ATGAACTTAT AACTTCCAAG CATGCAAACC
     2221 AAAAGGGAGA ACAAGTAATC CAAGTAGACA CGGGAATTGG ATTCTTGGAT CACATGTATC
     2281 ATGCACTGGC TAAACATGCA GGCTGGAGCT TACGACTTTA CTCAAGAGGT GATTTAATCA
     2341 TCGATGATCA TCACACTGCA GAAGATACTG CTATTGCACT TGGTATTGCA TTCAAGCAGG
     2401 CTATGGGTAA CTTTGCCGGC GTTAAAAGAT TTGGACATGC TTATTGTCCA CTTGACGAAG
     2461 CTCTTTCTAG AAGCGTAGTT GACTTGTCGG GACGGCCCTA TGCTGTTATC GATTTGGGAT
     2521 TAAAGCGTGA AAAGGTTGGG GAATTGTCCT GTGAAATGAT CCCTCACTTA CTATATTCCT
     2581 TTTCGGTAGC AGCTGGAATT ACTTTGCATG TTACCTGCTT ATATGGTAGT AATGACCATC
     2641 ATCGTGCTGA AAGCGCTTTT AAATCTCTGG CTGTTGCCAT GCGCGCGGCT ACTAGTCTTA
     2701 CTGGAAGTTC TGAAGTCCCA AGCACGAAGG GAGTGTTGTA AAGAGTACTG ACAATAAAAA
     2761 GATTCTTGTT TTCAAGAACT TGTCATTTGT ATAGTTTTTT TATATTGTAG TTGTTCTATT
     2821 TTAATCAAAT GTTAGCGTGA TTTATATTTT TTTTCGCCTC GACATCATCT GCCCAGATGC
     2881 GAAGTTAAGT GCGCAGAAAG TAATATCATG CGTCAATCGT ATGTGAATGC TGGTCGCTAT
     2941 ACTGCTGTCG ATTCGATACT AACGCCGCCA TCCAGTTTAA ACGAGCTCGA ATTCATCGAG
     3001 TCGACTTGGT TGAACACGTT GCCAAGGCTT AAGTGAATTT ACTTTAAATC TTGCATTTAA
     3061 ATAAATTTTC TTTTTATAGC TTTATGACTT AGTTTCAATT TATATACTAT TTTAATGACA
     3121 TTTTCGATTC GGATCCCTGG CGTAATAGCG AAGAGGCCCG CACCGATCGC CCTTCCCAAC
     3181 AGTTGCGCAG CCTGAATGGC GAATGGCGCC TGATGCGGTA TTTTCTCCTT ACGCATCTGT
     3241 GCGGTATTTC ACACCGCATA GGGTAATAAC TGATATAATT AAATTGAAGC TCTAATTTGT
     3301 GAGTTTAGTA TACATGCATT TACTTATAAT ACAGTTTTCT ACATAAGAAC ACCTTTGGTG
     3361 GAGGGAACAT CGTTGGTACC ATTGGGCGAG GTGGCTTCTC TTATGGCAAC CGCAAGAGCC
     3421 TTGAACGCAC TCTCACTACG GTGATGATCA TTCTTGCCTC GCAGACAATC AACGTGGAGG
     3481 GTAATTCTGC TAGCCTCTGC AAAGCTTTCA AGAAAATGCG GGATCATCTC GCAAGAGAGA
     3541 TCTCCTACTT TCTCCCTTTG CAAACCAAGT TCGACAACTG CGTACGGCCT GTTCGAAAGA
     3601 TCTACCACCG CTCTGGAAAG TGCCTCATCC AAAGGCGCAA ATCCTGATCC AAACCTTTTT
     3661 ACTCCACGCA CGGCCCCTAG GGCCTCTTTA AAAGCTTGAC CGAGAGCAAT CCCGCAGTCT
     3721 TCAGTGGTGT GATGGTCGTC TATGTGTAAG TCACCAATGC ACTCAACGAT TAGCGACCAG
     3781 CCGGAATGCT TGGCCAGAGC ATGTATCATA TGGTCCAGAA ACCCTATACC TGTGTGGACG
```

```
     3841 TTAATCACTT GCGATTGTGT GGCCTGTTCT GCTACTGCTT CTGCCTCTTT TTCTGGGAAG
     3901 ATCGAGTGCT CTATCGCTAG GGGACCACCC TTTAAAGAGA TCGCAATCTG AATCTTGGTT
     3961 TCATTTGTAA TACGCTTTAC TAGGGCTTTC TGCTCTGTCA TGATTTATCT TCGTTTCGGT
     4021 TTTTGTTCTG TGCAGTTGGG TTAAGAATAC TGGGCAATTT CATGTTTCTT CAACACTACA
     4081 TATGCGTATA TATACCAATC TAAGTCTGTG CTCCTTCCTT CGTTCTTCCT TCTGTTCGGA
     4141 GATTACCGAA TCAAAAAAAT TTCAAAGAAA CCGAAATCAA AAAAAAGAAT AAAAAAAAAA
     4201 TGATGAATTG AAAAGCTCTT GTTACCCATC ATTGAATTTT GAACATCCGA ACCTGGGAGT
     4261 TTTCCCTGAA ACAGATAGTA TATTTGAACC TGTATAATAA TATATAGTCT AGCGCTTTAC
     4321 GGAAGACAAT GTATGTATTT CGGTTCCTGG AGAAACTATT GCATCTATTG CATAGGTAAT
     4381 CTTGCACGTC GCATCCCCGG TTCATTTTCT GCGTTTCCAT CTTGCACTTC AATAGCATAT
     4441 CTTTGTTAAC GAAGCATCTG TGCTTCATTT TGTAGAACAA AAATGCAACG CGAGAGCGCT
     4501 AATTTTTCAA ACAAAGAATC TGAGCTGCAT TTTTACAGAA CAGAAATGCA ACGCGAAAGC
     4561 GCTATTTTAC CAACGAAGAA TCTGTGCTTC ATTTTTGTAA AACAAAAATG CAACGCGAGA
     4621 GCGCTAATTT TTCAAACAAA GAATCTGAGC TGCATTTTTA CAGAACAGAA ATGCAACGCG
     4681 AGAGCGCTAT TTTACCAACA AAGAATCTAT ACTTCTTTTT TGTTCTACAA AAATGCATCC
     4741 CGAGAGCGCT ATTTTTCTAA CAAAGCATCT TAGATTACTT TTTTTCTCCT TTGTGCGCTC
     4801 TATAATGCAG TCTCTTGATA ACTTTTTGCA CTGTAGGTCC GTTAAGGTTA GAAGAAGGCT
     4861 ACTTTGGTGT CTATTTTCTC TTCCATAAAA AAAGCCTGAC TCCACTTCCC GCGTTTACTG
     4921 ATTACTAGCG AAGCTGCGGG TGCATTTTTT CAAGATAAAG GCATCCCCGA TTATATTCTA
     4981 TACCGATGTG GATTGCGCAT ACTTTGTGAA CAGAAAGTGA TAGCGTTGAT GATTCTTCAT
     5041 TGGTCAGAAA ATTATGAACG GTTTCTTCTA TTTTGTCTCT ATATACTACG TATAGGAAAT
     5101 GTTTACATTT TCGTATTGTT TTCGATTCAC TCTATGAATA GTTCTTACTA CAATTTTTTT
     5161 GTCTAAAGAG TAATACTAGA GATAAACATA AAAAATGTAG AGGTCGAGTT TAGATGCAAG
     5221 TTCAAGGAGC GAAAGGTGGA TGGGTAGGTT ATATAGGGAT ATAGCACAGA GATATATAGC
     5281 AAAGAGATAC TTTTGAGCAA TGTTTGTGGA AGCGGTATTC GCAATATTTT AGTAGCTCGT
     5341 TACAGTCCGG TGCGTTTTTG GTTTTTTGAA AGTGCGTCTT CAGAGCGCTT TTGGTTTTCA
     5401 AAAGCGCTCT GAAGTTCCTA TACTTTCTAG CTAGAGAATA GGAACTTCGG AATAGGAACT
     5461 TCAAAGCGTT TCCGAAAACG AGCGCTTCCG AAAATGCAAC GCGAGCTGCG CACATACAGC
     5521 TCACTGTTCA CGTCGCACCT ATATCTGCGT GTTGCCTGTA TATATATATA CATGAGAAGA
     5581 ACGGCATAGT GCGTGTTTAT GCTTAAATGC GTTATGGTGC ACTCTCAGTA CAATCTGCTC
     5641 TGATGCCGCA TAGTTAAGCC AGCCCCGACA CCCGCCAACA CCCGCTGACG CGCCCTGACG
     5701 GGCTTGTCTG CTCCCGGCAT CCGCTTACAG ACAAGCTGTG ACCGTCTCCG GGAGCTGCAT
     5761 GTGTCAGAGG TTTTCACCGT CATCACCGAA ACGCGCGAGA CGAAAGGGCC TCGTGATACG
     5821 CCTATTTTTA TAGGTTAATG TCATGATAAT AATGGTTTCT TAGACGTCAG GTGGCACTTT
     5881 TCGGGGAAAT GTGCGCGGAA CCCCTATTTG TTTATTTTTC TAAATACATT CAAATATGTA
     5941 TCCGCTCATG AGACAATAAC CCTGATAAAT GCTTCAATAA TATTGAAAAA GGAAGAGTAT
     6001 GAGTATTCAA CATTTCCGTG TCGCCCTTAT TCCCTTTTTT GCGGCATTTT GCCTTCCTGT
     6061 TTTTGCTCAC CCAGAAACGC TGGTGAAAGT AAAAGATGCT GAAGATCAGT TGGGTGCACG
     6121 AGTGGGTTAC ATCGAACTGG ATCTCAACAG CGGTAAGATC CTTGAGAGTT TTCGCCCCGA
     6181 AGAACGTTTT CCAATGATGA GCACTTTTAA AGTTCTGCTA TGTGGCGCGG TATTATCCCG
     6241 TATTGACGCC GGGCAAGAGC AACTCGGTCG CCGCATACAC TATTCTCAGA ATGACTTGGT
     6301 TGAGTACTCA CCAGTCACAG AAAAGCATCT TACGGATGGC ATGACAGTAA GAGAATTATG
     6361 CAGTGCTGCC ATAACCATGA GTGATAACAC TGCGGCCAAC TTACTTCTGA CAACGATCGG
     6421 AGGACCGAAG GAGCTAACCG CTTTTTTGCA CAACATGGGG GATCATGTAA CTCGCCTTGA
     6481 TCGTTGGGAA CCGGAGCTGA ATGAAGCCAT ACCAAACGAC GAGCGTGACA CCACGATGCC
     6541 TGTAGCAATG GCAACAACGT TGCGCAAACT ATTAACTGGC GAACTACTTA CTCTAGCTTC
     6601 CCGGCAACAA TTAATAGACT GGATGGAGGC GGATAAAGTT GCAGGACCAC TTCTGCGCTC
     6661 GGCCCTTCCG GCTGGCTGGT TTATTGCTGA TAAATCTGGA GCCGGTGAGC GTGGGTCTCG
     6721 CGGTATCATT GCAGCACTGG GGCCAGATGG TAAGCCCTCC CGTATCGTAG TTATCTACAC
     6781 GACGGGGAGT CAGGCAACTA TGGATGAACG AAATAGACAG ATCGCTGAGA TAGGTGCCTC
     6841 ACTGATTAAG CATTGGTAAC TGTCAGACCA AGTTTACTCA TATATACTTT AGATTGATTT
     6901 AAAACTTCAT TTTTAATTTA AAAGGATCTA GGTGAAGATC CTTTTTGATA ATCTCATGAC
     6961 CAAAATCCCT TAACGTGAGT TTTCGTTCCA CTGAGCGTCA GACCCCGTAG AAAAGATCAA
     7021 AGGATCTTCT TGAGATCCTT TTTTTCTGCG CGTAATCTGC TGCTTGCAAA CAAAAAAACC
     7081 ACCGCTACCA GCGGTGGTTT GTTTGCCGGA TCAAGAGCTA CCAACTCTTT TTCCGAAGGT
     7141 AACTGGCTTC AGCAGAGCGC AGATACCAAA TACTGTCCTT CTAGTGTAGC CGTAGTTAGG
     7201 CCACCACTTC AAGAACTCTG TAGCACCGCC TACATACCTC GCTCTGCTAA TCCTGTTACC
     7261 AGTGGCTGCT GCCAGTGGCG ATAAGTCGTG TCTTACCGGG TTGGACTCAA GACGATAGTT
     7321 ACCGGATAAG GCGCAGCGGT CGGGCTGAAC GGGGGGTTCG TGCACACAGC CCAGCTTGGA
     7381 GCGAACGACC TACACCGAAC TGAGATACCT ACAGCGTGAG CATTGAGAAA GCGCCACGCT
     7441 TCCCGAAGGG AGAAAGGCGG ACAGGTATCC GGTAAGCGGC AGGGTCGGAA CAGGAGAGCG
     7501 CACGAGGGAG CTTCCAGGGG GAAACGCCTG GTATCTTTAT AGTCCTGTCG GGTTTCGCCA
     7561 CCTCTGACTT GAGCGTCGAT TTTTGTGATG CTCGTCAGGG GGGCGGAGCC TATGGAAAAA
     7621 CGCCAGCAAC GCGGCCTTTT TACGGTTCCT GGCCTTTTGC TGGCCTTTTG CTCACATGTT
     7681 CTTTCCTGCG TTATCCCCTG ATTCTGTGGA TAACCGTATT ACCGCCTTTG AGTGAGCTGA
     7741 TACCGCTCGC CGCAGCCGAA CGACCGAGCG CAGCGAGTCA GTGAGCGAGG AAGCGGAAGA
     7801 GCGCCCAATA CGCAAACCGC CTCTCCCCGC GCGTTGGCCG ATTCATTAAT C
//
LOCUS       YEP_SAPPHIRE_HIS3      7851 BP    DNA     CIRCULAR UNA 11-JAN-
2011
```

```
DEFINITION
FEATURES             LOCATION/QUALIFIERS
     PROMOTER        6..719
                     /LABEL=TDH3PROM
     CDS             745..1461
                     /LABEL=SAPPHIRE
     TERMINATOR      1469..1691
                     /LABEL="ADH1 TERM"
     TERMINATOR      3000..3130
                     /LABEL="TDH3 TERM"
     CDS             COMPLEMENT(3339..4001)
                     /LABEL=HIS3
     REP_ORIGIN      4218..5612
                     /LABEL="2 MICRON"
     CDS             5999..6859
                     /LABEL=AMPR
     REP_ORIGIN      7014..7633
                     /LABEL=PBR322
ORIGIN
        1 GGATCCGTTA GAATCATTTT GAATAAAAAA CACGCTTTTT CAGTTCGAGT TTATCATTAT
       61 CAATACTGCC ATTTCAAAGA ATACGTAAAT AATTAATAGT AGTGATTTTC CTAACTTTAT
      121 TTAGTCAAAA AATTAGCCTT TTAATTCTGC TGTAACCCGT ACATGCCCAA AATAGGGGGC
      181 GGGTTACACA GAATATATAA CATCGTAGGT GTCTGGGTGA ACAGTTTATT CCTGGCATCC
      241 ACTAAATATA ATGGAGCCCG CTTTTTAAGC TGGCATCCAG AAAAAAAAAG AATCCCAGCA
      301 CCAAAATATT GTTTTCTTCA CCAACCATCA GTTCATAGGT CCATTCTCTT AGCGCAACTA
      361 CAGAGAACAG GGGCACAAAC AGGCAAAAAA CGGGCACAAC CTCAATGGAG TGATGCAACC
      421 TGCCTGGAGT AAATGATGAC ACAAGGCAAT TGACCCACGC ATGTATCTAT CTCATTTTCT
      481 TACACCTTCT ATTACCTTCT GCTCTCTCTG ATTTGGAAAA AGCTGAAAAA AAAGGTTGAA
      541 ACCAGTTCCC TGAAATTATT CCCCTACTTG ACTAATAAGT ATATAAAGAC GGTAGGTATT
      601 GATTGTAATT CTGTAAATCT ATTTCTTAAA CTTCTTAAAT TCTACTTTTA TAGTTAGTCT
      661 TTTTTTTAGT TTTAAAACAC CAAGAACTTA GTTTCGAATA AACACACATA AACAAACAAA
      721 GGTGACGGTG CTGGTTTAAT TAACATGTCT AAAGGTGAAG AATTATTCAC TGGTGTTGTC
      781 CCAATTTTGG TTGAATTAGA TGGTGATGTT AATGGTCACA AATTTTCTGT CTCCGGTGAA
      841 GGTGAAGGTG ATGCTACTTA CGGTAAATTG ACCTTAAAAT TTATTTGTAC TACTGGTAAA
      901 TTGCCAGTTC CATGGCCAAC CTTAGTCACT ACTTTTTCTT ATGGTGTTCA ATGTTTTGCT
      961 AGATACCCAG ATCATATGAA ACAACATGAC TTTTTCAAGT CTGCCATGCC AGAAGGTTAT
     1021 GTTCAAGAAA GAACTATTTT TTTCAAAGAT GACGGTAACT ACAAGACCAG AGCTGAAGTC
     1081 AAGTTTGAAG GTGATACCTT AGTTAATAGA ATCGAATTAA AAGGTATTGA TTTTAAAGAA
     1141 GATGGTAACA TTTTAGGTCA CAAATTGGAA TACAACTTTA ACTCTCACAA TGTTTACATC
     1201 ATGGCTGACA AACAAAAGAA TGGTATCAAA GTTAACTTCA AAATTAGACA CAACATTGAA
     1261 GATGGTTCTG TTCAATTAGC TGACCATTAT CAACAAAATA CTCCAATTGG TGATGGTCCA
     1321 GTCTTGTTAC CAGACAACCA TTACTTATCC ATTCAATCTG CCTTATCCAA AGATCCAAAC
     1381 GAAAAGAGAG ACCACATGGT CTTGTTAGAA TTTGTTACTG CTGCTGGTAT TACCCATGGT
     1441 ATGGATGAAT TGTACAAATA AGGCGCGCCA CTTCTAAATA AGCGAATTTC TTATGATTTA
     1501 TGATTTTTAT TATTAAATAA GTTATAAAAA AAATAAGTGT ATACAAATTT TAAAGTGACT
     1561 CTTAGGTTTT AAAACGAAAA TTCTTATTCT TGAGTAACTC TTTCCTGTAG GTCAGGTTGC
     1621 TTTCTCAGGT ATAGTATGAG GTCGCTCTTA TTGACCACAC CTCTACCGGC AGATCCGCTA
     1681 GGGATAACAG GGTAATATAG ATCTGTTTAG CTTGCCTCGT CCCCGCCGGG TCACCCGGCC
     1741 AGCGACATGG AGGCCCAGAA TACCCTCCTT GACAGTCTTG ACGTGCGCAG CTCAGGGGCA
     1801 TGATGTGACT GTCGCCCGTA CATTTAGCCC ATACATCCCC ATGTATAATC ATTTGCATCC
     1861 ATACATTTTG ATGGCCGCAC GGCGCGAAGC AAAAATTACG GCTCCTCGCT GCAGACCTGC
     1921 GAGCAGGGAA ACGCTCCCCT CACAGACGCG TTGAATTGTC CCCACGCCGC GCCCCTGTAG
     1981 AGAAATATAA AAGGTTAGGA TTTGCCACTG AGGTTCTTCT TTCATATACT TCCTTTTAAA
     2041 ATCTTGCTAG GATACAGTTC TCACATCACA TCCGAACATA AACAACCATG GGTAGGAGGG
     2101 CTTTTGTAGA AAGAAATACG AACGAAACGA AAATCAGCGT TGCCATCGCT TTGGACAAAG
     2161 CTCCCTTACC TGAAGAGTCG AATTTTATTG ATGAACTTAT AACTTCCAAG CATGCAAACC
     2221 AAAAGGGAGA ACAAGTAATC CAAGTAGACA CGGGAATTGG ATTCTTGGAT CACATGTATC
     2281 ATGCACTGGC TAAACATGCA GGCTGGAGCT TACGACTTTA CTCAAGAGGT GATTTAATCA
     2341 TCGATGATCA TCACACTGCA GAAGATACTG CTATTGCACT TGGTATTGCA TTCAAGCAGG
     2401 CTATGGGTAA CTTTGCCGGC GTTAAAAGAT TTGGACATGC TTATTGTCCA CTTGACGAAG
     2461 CTCTTTCTAG AAGCGTAGTT GACTTGTCGG GACGGCCCTA TGCTGTTATC GATTTGGGAT
     2521 TAAAGCGTGA AAAGGTTGGG GAATTGTCCT GTGAAATGAT CCCTCACTTA CTATATTCCT
     2581 TTTCGGTAGC AGCTGGAATT ACTTTGCATG TTACCTGCTT ATATGGTAGT AATGACCATC
     2641 ATCGTGCTGA AAGCGCTTTT AAATCTCTGG CTGTTGCCAT GCGCGCGGCT ACTAGTCTTA
     2701 CTGGAAGTTC TGAAGTCCCA AGCACGAAGG GAGTGTTGTA AAGAGTACTG ACAATAAAAA
     2761 GATTCTTGTT TTCAAGAACT TGTCATTTGT ATAGTTTTTT TATATTGTAG TTGTTCTATT
     2821 TTAATCAAAT GTTAGCGTGA TTTATATTTT TTTTCGCCTC GACATCATCT GCCCAGATGC
     2881 GAAGTTAAGT GCGCAGAAAG TAATATCATG CGTCAATCGT ATGTGAATGC TGGTCGCTAT
     2941 ACTGCTGTCG ATTCGATACT AACGCCGCCA TCCAGTTTAA ACGAGCTCGA ATTCATCGAG
     3001 TCGACTTGGT TGAACACGTT GCCAAGGCTT AAGTGAATTT ACTTTAAATC TTGCATTTAA
```

3061 ATAAATTTTC TTTTTATAGC TTTATGACTT AGTTTCAATT TATATACTAT TTTAATGACA
3121 TTTTCGATTC GGATCCCTGG CGTAATAGCG AAGAGGCCCG CACCGATCGC CCTTCCCAAC
3181 AGTTGCGCAG CCTGAATGGC GAATGGCGCC TGATGCGGTA TTTTCTCCTT ACGCATCTGT
3241 GCGGTATTTC ACACCGCATA GGGTAATAAC TGATATAATT AAATTGAAGC TCTAATTTGT
3301 GAGTTTAGTA TACATGCATT TACTTATAAT ACAGTTTTCT ACATAAGAAC ACCTTTGGTG
3361 GAGGGAACAT CGTTGGTACC ATTGGGCGAG GTGGCTTCTC TTATGGCAAC CGCAAGAGCC
3421 TTGAACGCAC TCTCACTACG GTGATGATCA TTCTTGCCTC GCAGACAATC AACGTGGAGG
3481 GTAATTCTGC TAGCCTCTGC AAAGCTTTCA AGAAAATGCG GGATCATCTC GCAAGAGAGA
3541 TCTCCTACTT TCTCCCTTTG CAAACCAAGT TCGACAACTG CGTACGGCCT GTTCGAAAGA
3601 TCTACCACCG CTCTGGAAAG TGCCTCATCC AAAGGCGCAA ATCCTGATCC AAACCTTTTT
3661 ACTCCACGCA CGGCCCCTAG GGCCTCTTTA AAAGCTTGAC CGAGAGCAAT CCCGCAGTCT
3721 TCAGTGGTGT GATGGTCGTC TATGTGTAAG TCACCAATGC ACTCAACGAT TAGCGACCAG
3781 CCGGAATGCT TGGCCAGAGC ATGTATCATA TGGTCCAGAA ACCCTATACC TGTGTGGACG
3841 TTAATCACTT GCGATTGTGT GGCCTGTTCT GCTACTGCTT CTGCCTCTTT TTCTGGGAAG
3901 ATCGAGTGCT CTATCGCTAG GGGACCACCC TTTAAAGAGA TCGCAATCTG AATCTTGGTT
3961 TCATTTGTAA TACGCTTTAC TAGGGCTTTC TGCTCTGTCA TGATTTATCT TCGTTTCGGT
4021 TTTGTTCTG TGCAGTTGGG TTAAGAATAC TGGGCAATTT CATGTTTCTT CAACACTACA
4081 TATGCGTATA TATACCAATC TAAGTCTGTG CTCCTTCCTT CGTTCTTCCT TCTGTTCGGA
4141 GATTACCGAA TCAAAAAAAT TTCAAAGAAA CCGAAATCAA AAAAAAGAAT AAAAAAAAAA
4201 TGATGAATTG AAAAGCTCTT GTTACCCATC ATTGAATTTT GAACATCCGA ACCTGGGAGT
4261 TTTCCCTGAA ACAGATAGTA TATTTGAACC TGTATAATAA TATATAGTCT AGCGCTTTAC
4321 GGAAGACAAT GTATGTATTT CGGTTCCTGG AGAAACTATT GCATCTATTG CATAGGTAAT
4381 CTTGCACGTC GCATCCCCGG TTCATTTTCT GCGTTTCCAT CTTGCACTTC AATAGCATAT
4441 CTTTGTTAAC GAAGCATCTG TGCTTCATTT TGTAGAACAA AAATGCAACG CGAGAGCGCT
4501 AATTTTTCAA ACAAAGAATC TGAGCTGCAT TTTTACAGAA CAGAAATGCA ACGCGAAAGC
4561 GCTATTTTAC CAACGAAGAA TCTGTGCTTC ATTTTTGTAA AACAAAAATG CAACGCGAGA
4621 GCGCTAATTT TTCAAACAAA GAATCTGAGC TGCATTTTTA CAGAACAGAA ATGCAACGCG
4681 AGAGCGCTAT TTTACCAACA AAGAATCTAT ACTTCTTTTT TGTTCTACAA AAATGCATCC
4741 CGAGAGCGCT ATTTTTCTAA CAAAGCATCT TAGATTACTT TTTTTCTCCT TTGTGCGCTC
4801 TATAATGCAG TCTCTTGATA ACTTTTTGCA CTGTAGGTCC GTTAAGGTTA GAAGAAGGCT
4861 ACTTTGGTGT CTATTTTCTC TTCCATAAAA AAAGCCTGAC TCCACTTCCC GCGTTTACTG
4921 ATTACTAGCG AAGCTGCGGG TGCATTTTTT CAAGATAAAG GCATCCCCGA TTATATTCTA
4981 TACCGATGTG GATTGCGCAT ACTTTGTGAA CAGAAAGTGA TAGCGTTGAT GATTCTTCAT
5041 TGGTCAGAAA ATTATGAACG GTTTCTTCTA TTTTGTCTCT ATATACTACG TATAGGAAAT
5101 GTTTACATTT TCGTATTGTT TTCGATTCAC TCTATGAATA GTTCTTACTA CAATTTTTTT
5161 GTCTAAAGAG TAATACTAGA GATAAACATA AAAAATGTAG AGGTCGAGTT TAGATGCAAG
5221 TTCAAGGAGC GAAAGGTGGA TGGGTAGGTT ATATAGGGAT ATAGCACAGA GATATATAGC
5281 AAAGAGATAC TTTTGAGCAA TGTTTGTGGA AGCGGTATTC GCAATATTTT AGTAGCTCGT
5341 TACAGTCCGG TGCGTTTTTG GTTTTTTGAA AGTGCGTCTT CAGAGCGCTT TTGGTTTTCA
5401 AAAGCGCTCT GAAGTTCCTA TACTTTCTAG CTAGAGAATA GGAACTTCGG AATAGGAACT
5461 TCAAAGCGTT TCCGAAAACG AGCGCTTCCG AAAATGCAAC GCGAGCTGCG CACATACAGC
5521 TCACTGTTCA CGTCGCACCT ATATCTGCGT GTTGCCTGTA TATATATATA CATGAGAAGA
5581 ACGGCATAGT GCGTGTTTAT GCTTAAATGC GTTATGGTGC ACTCTCAGTA CAATCTGCTC
5641 TGATGCCGCA TAGTTAAGCC AGCCCCGACA CCCGCCAACA CCCGCTGACG CGCCCTGACG
5701 GGCTTGTCTG CTCCCGGCAT CCGCTTACAG ACAAGCTGTG ACCGTCTCCG GGAGCTGCAT
5761 GTGTCAGAGG TTTTCACCGT CATCACCGAA ACGCGCGAGA CGAAAGGGCC TCGTGATACG
5821 CCTATTTTTA TAGGTTAATG TCATGATAAT AATGGTTTCT TAGACGTCAG GTGGCACTTT
5881 TCGGGGAAAT GTGCGCGGAA CCCCTATTTG TTTATTTTTC TAAATACATT CAAATATGTA
5941 TCCGCTCATG AGACAATAAC CCTGATAAAT GCTTCAATAA TATTGAAAAA GGAAGAGTAT
6001 GAGTATTCAA CATTTCCGTG TCGCCCTTAT TCCCTTTTTT GCGGCATTTT GCCTTCCTGT
6061 TTTTGCTCAC CCAGAAACGC TGGTGAAAGT AAAAGATGCT GAAGATCAGT TGGGTGCACG
6121 AGTGGGTTAC ATCGAACTGG ATCTCAACAG CGGTAAGATC CTTGAGAGTT TTCGCCCCGA
6181 AGAACGTTTT CCAATGATGA GCACTTTTAA AGTTCTGCTA TGTGGCGCGG TATTATCCCG
6241 TATTGACGCC GGGCAAGAGC AACTCGGTCG CCGCATACAC TATTCTCAGA ATGACTTGGT
6301 TGAGTACTCA CCAGTCACAG AAAAGCATCT TACGGATGGC ATGACAGTAA GAGAATTATG
6361 CAGTGCTGCC ATAACCATGA GTGATAACAC TGCGGCCAAC TTACTTCTGA CAACGATCGG
6421 AGGACCGAAG GAGCTAACCG CTTTTTTGCA CAACATGGGG GATCATGTAA CTCGCCTTGA
6481 TCGTTGGGAA CCGGAGCTGA ATGAAGCCAT ACCAAACGAC GAGCGTGACA CCACGATGCC
6541 TGTAGCAATG GCAACAACGT TGCGCAAACT ATTAACTGGC GAACTACTTA CTCTAGCTTC
6601 CCGGCAACAA TTAATAGACT GGATGGAGGC GGATAAAGTT GCAGGACCAC TTCTGCGCTC
6661 GGCCCTTCCG GCTGGCTGGT TTATTGCTGA TAAATCTGGA GCCGGTGAGC GTGGGTCTCG
6721 CGGTATCATT GCAGCACTGG GGCCAGATGG TAAGCCCTCC CGTATCGTAG TTATCTACAC
6781 GACGGGGAGT CAGGCAACTA TGGATGAACG AAATAGACAG ATCGCTGAGA TAGGTGCCTC
6841 ACTGATTAAG CATTGGTAAC TGTCAGACCA AGTTTACTCA TATATACTTT AGATTGATTT
6901 AAAACTTCAT TTTTAATTTA AAAGGATCTA GGTGAAGATC CTTTTTGATA ATCTCATGAC
6961 CAAAATCCCT TAACGTGAGT TTTCGTTCCA CTGAGCGTCA GACCCCGTAG AAAAGATCAA
7021 AGGATCTTCT TGAGATCCTT TTTTTCTGCG CGTAATCTGC TGCTTGCAAA CAAAAAAACC
7081 ACCGCTACCA GCGGTGGTTT GTTTGCCGGA TCAAGAGCTA CCAACTCTTT TTCCGAAGGT
7141 AACTGGCTTC AGCAGAGCGC AGATACCAAA TACTGTCCTT CTAGTGTAGC CGTAGTTAGG
7201 CCACCACTTC AAGAACTCTG TAGCACCGCC TACATACCTC GCTCTGCTAA TCCTGTTACC

```
     7261 AGTGGCTGCT GCCAGTGGCG ATAAGTCGTG TCTTACCGGG TTGGACTCAA GACGATAGTT
     7321 ACCGGATAAG GCGCAGCGGT CGGGCTGAAC GGGGGGTTCG TGCACACAGC CCAGCTTGGA
     7381 GCGAACGACC TACACCGAAC TGAGATACCT ACAGCGTGAG CATTGAGAAA GCGCCACGCT
     7441 TCCCGAAGGG AGAAAGGCGG ACAGGTATCC GGTAAGCGGC AGGGTCGGAA CAGGAGAGCG
     7501 CACGAGGGAG CTTCCAGGGG GAAACGCCTG GTATCTTTAT AGTCCTGTCG GGTTTCGCCA
     7561 CCTCTGACTT GAGCGTCGAT TTTTGTGATG CTCGTCAGGG GGGCGGAGCC TATGGAAAAA
     7621 CGCCAGCAAC GCGGCCTTTT TACGGTTCCT GGCCTTTTGC TGGCCTTTTG CTCACATGTT
     7681 CTTTCCTGCG TTATCCCCTG ATTCTGTGGA TAACCGTATT ACCGCCTTTG AGTGAGCTGA
     7741 TACCGCTCGC CGCAGCCGAA CGACCGAGCG CAGCGAGTCA GTGAGCGAGG AAGCGGAAGA
     7801 GCGCCCAATA CGCAAACCGC CTCTCCCCGC GCGTTGGCCG ATTCATTAAT C
//
LOCUS       YEP_CHERRY_LEU2      6751 BP    DNA     CIRCULAR UNA 10-JAN-2011
DEFINITION
FEATURES             LOCATION/QUALIFIERS
     PROMOTER        6..719
                     /LABEL="TDH3 PROM"
     CDS             727..1437
                     /LABEL=MCHERRY
     TERMINATOR      1468..1598
                     /LABEL="TDH3 TERM"
     CDS             COMPLEMENT(1807..2901)
                     /LABEL=LEU2
     REP_ORIGIN      3118..4512
                     /LABEL="2 MICRON"
     CDS             4899..5759
                     /LABEL=AMPR
     REP_ORIGIN      5914..6533
                     /LABEL=PBR332
ORIGIN
        1 GGATCCGTTA GAATCATTTT GAATAAAAAA CACGCTTTTT CAGTTCGAGT TTATCATTAT
       61 CAATACTGCC ATTTCAAAGA ATACGTAAAT AATTAATAGT AGTGATTTTC CTAACTTTAT
      121 TTAGTCAAAA AATTAGCCTT TTAATTCTGC TGTAACCCGT ACATGCCCAA AATAGGGGGC
      181 GGGTTACACA GAATATATAA CATCGTAGGT GTCTGGGTGA ACAGTTTATT CCTGGCATCC
      241 ACTAAATATA ATGGAGCCCG CTTTTTAAGC TGGCATCCAG AAAAAAAAAG AATCCCAGCA
      301 CCAAAATATT GTTTTCTTCA CCAACCATCA GTTCATAGGT CCATTCTCTT AGCGCAACTA
      361 CAGAGAACAG GGGCACAAAC AGGCAAAAAA CGGGCACAAC CTCAATGGAG TGATGCAACC
      421 TGCCTGGAGT AAATGATGAC ACAAGGCAAT TGACCCACGC ATGTATCTAT CTCATTTTCT
      481 TACACCTTCT ATTACCTTCT GCTCTCTCTG ATTTGGAAAA AGCTGAAAAA AAAGGTTGAA
      541 ACCAGTTCCC TGAAATTATT CCCCTACTTG ACTAATAAGT ATATAAAGAC GGTAGGTATT
      601 GATTGTAATT CTGTAAATCT ATTTCTTAAA CTTCTTAAAT TCTACTTTTA TAGTTAGTCT
      661 TTTTTTTAGT TTTAAAACAC CAAGAACTTA GTTTCGAATA AACACACATA AACAAACAAA
      721 GAATTCATGG TTTCAAAAGG TGAAGAAGAT AATATGGCTA TTATTAAAGA ATTTATGAGA
      781 TTTAAAGTTC ATATGGAAGG TTCAGTTAAT GGTCATGAAT TTGAAATTGA AGGTGAAGGT
      841 GAAGGTAGAC CATATGAAGG TACTCAAACT GCTAAATTGA AAGTTACTAA AGGTGGTCCA
      901 TTACCATTTG CTTGGGATAT TTTGTCACCA CAATTTATGT ATGGTTCAAA AGCTTATGTT
      961 AAACATCCAG CTGATATTCC AGATTATTTA AAATTGTCAT TTCCAGAAGG TTTTAAATGG
     1021 GAAAGAGTTA TGAATTTTGA AGATGGTGGT GTTGTTACTG TTACTCAAGA TTCATCATTA
     1081 CAAGATGGTG AATTTATTTA TAAAGTTAAA TTGAGAGGTA CTAATTTTCC ATCAGATGGT
     1141 CCAGTTATGC AAAAAAAAAC TATGGGTTGG GAAGCTTCAT CAGAAAGAAT GTATCCAGAA
     1201 GATGGTGCTT TAAAAGGTGA AATTAAACAA AGATTGAAAT TAAAAGATGG TGGTCATTAT
     1261 GATGCTGAAG TTAAAACTAC TTATAAAGCT AAAAAACCAG TTCAATTACC AGGTGCTTAT
     1321 AATGTTAATA TTAAATTGGA TATTACTTCA CATAATGAAG ATTATACTAT TGTTGAACAA
     1381 TATGAAAGAG CTGAAGGTAG ACATTCAACT GGTGGTATGG ATGAATTATA TAAATAAGGT
     1441 ACCGCTCGAG CAGCTGTGAT TGATTGAGTC GACTTGGTTG AACACGTTGC CAAGGCTTAA
     1501 GTGAATTTAC TTTAAATCTT GCATTTAAAT AAATTTTCTT TTTATAGCTT TATGACTTAG
     1561 TTTCAATTTA TATACTATTT TAATGACATT TTCGATTCGG ATCCCTGGCG TAATAGCGAA
     1621 GAGGCCCGCA CCGATCGCCC TTCCCAACAG TTGCGCAGCC TGAATGGCGA ATGGCGCCTG
     1681 ATGCGGTATT TTCTCCTTAC GCATCTGTGC GGTATTTCAC ACCGCATAGG GTAATAACTG
     1741 ATATAATTAA ATTGAAGCTC TAATTTGTGA GTTTAGTATA CATGCATTTA CTTATAATAC
     1801 AGTTTTTTAA GCAAGGATTT TCTTAACTTC TTCGGCGACA GCATCACCGA CTTCGGTGGT
     1861 ACTGTTGGAA CCACCTAAAT CACCAGTTCT GATACCTGCA TCCAAAACCT TTTTAACTGC
     1921 ATCTTCAATG GCCTTACCTT CTTCAGGCAA GTTCAATGAC AATTTCAACA TCATTGCAGC
     1981 AGACAAGATA GTGGCGATAG GGTTGACCTT ATTCTTTGGC AAATCTGGAG CAGAACCGTG
     2041 GCATGGTTCG TACAAACCAA ATGCGGTGTT CTTGTCTGGC AAAGAGGCCA AGGACGCAGA
     2101 TGGCAACAAA CCCAAGGAAC CTGGGATAAC GGAGGCTTCA TCGGAGATGA TATCACCAAA
     2161 CATGTTGCTG GTGATTATAA TACCATTTAG GTGGGTTGGG TTCTTAACTA GGATCATGGC
     2221 GGCAGAATCA ATCAATTGAT GTTGAACCTT CAATGTAGGG AATTCGTTCT TGATGGTTTC
     2281 CTCCACAGTT TTTCTCCATA ATCTTGAAGA GGCCAAAACA TTAGCTTTAT CCAAGGACCA
     2341 AATAGGCAAT GGTGGCTCAT GTTGTAGGGC CATGAAAGCG GCCATTCTTG TGATTCTTTG
     2401 CACTTCTGGA ACGGTGTATT GTTCACTATC CCAAGCGACA CCATCACCAT CGTCTTCCTT
```

2461 TCTCTTACCA AAGTAAATAC CTCCCACTAA TTCTCTGACA ACAACGAAGT CAGTACCTTT
2521 AGCAAATTGT GGCTTGATTG GAGATAAGTC TAAAAGAGAG TCGGATGCAA AGTTACATGG
2581 TCTTAAGTTG GCGTACAATT GAAGTTCTTT ACGGATTTTT AGTAAACCTT GTTCAGGTCT
2641 AACACTACCG GTACCCCATT TAGGACCACC CACAGCACCT AACAAAACGG CATCAGCCTT
2701 CTTGGAGGCT TCCAGCGCCT CATCTGGAAG TGGAACACCT GTAGCATCGA TAGCAGCACC
2761 ACCAATTAAA TGATTTTCGA AATCGAACTT GACATTGGAA CGAACATCAG AAATAGCTTT
2821 AAGAACCTTA ATGGCTTCGG CTGTGATTTC TTGACCAACG TGGTCACCTG GCAAAACGAC
2881 GATCTTCTTA GGGGCAGACA TGATTTATCT TCGTTTCGGT TTTTGTTCTG TGCAGTTGGG
2941 TTAAGAATAC TGGGCAATTT CATGTTTCTT CAACACTACA TATGCGTATA TATACCAATC
3001 TAAGTCTGTG CTCCTTCCTT CGTTCTTCCT TCTGTTCGGA GATTACCGAA TCAAAAAAAT
3061 TTCAAAGAAA CCGAAATCAA AAAAAAGAAT AAAAAAAAAA TGATGAATTG AAAAGCTCTT
3121 GTTACCCATC ATTGAATTTT GAACATCCGA ACCTGGGAGT TTTCCCTGAA ACAGATAGTA
3181 TATTTGAACC TGTATAATAA TATATAGTCT AGCGCTTTAC GGAAGACAAT GTATGTATTT
3241 CGGTTCCTGG AGAAACTATT GCATCTATTG CATAGGTAAT CTTGCACGTC GCATCCCCGG
3301 TTCATTTTCT GCGTTTCCAT CTTGCACTTC AATAGCATAT CTTTGTTAAC GAAGCATCTG
3361 TGCTTCATTT TGTAGAACAA AAATGCAACG CGAGAGCGCT AATTTTTCAA ACAAAGAATC
3421 TGAGCTGCAT TTTTACAGAA CAGAAATGCA ACGCGAAAGC GCTATTTTAC CAACGAAGAA
3481 TCTGTGCTTC ATTTTTGTAA AACAAAAATG CAACGCGAGA GCGCTAATTT TTCAAACAAA
3541 GAATCTGAGC TGCATTTTTA CAGAACAGAA ATGCAACGCG AGAGCGCTAT TTTACCAACA
3601 AAGAATCTAT ACTTCTTTTT TGTTCTACAA AAATGCATCC CGAGAGCGCT ATTTTTCTAA
3661 CAAAGCATCT TAGATTACTT TTTTTCTCCT TTGTGCGCTC TATAATGCAG TCTCTTGATA
3721 ACTTTTTGCA CTGTAGGTCC GTTAAGGTTA GAAGAAGGCT ACTTTGGTGT CTATTTTCTC
3781 TTCCATAAAA AAAGCCTGAC TCCACTTCCC GCGTTTACTG ATTACTAGCG AAGCTGCGGG
3841 TGCATTTTTT CAAGATAAAG GCATCCCCGA TTATATTCTA TACCGATGTG GATTGCGCAT
3901 ACTTTGTGAA CAGAAAGTGA TAGCGTTGAT GATTCTTCAT TGGTCAGAAA ATTATGAACG
3961 GTTTCTTCTA TTTTGTCTCT ATATACTACG TATAGGAAAT GTTTACATTT TCGTATTGTT
4021 TTCGATTCAC TCTATGAATA GTTCTTACTA CAATTTTTTT GTCTAAAGAG TAATACTAGA
4081 GATAAACATA AAAAATGTAG AGGTCGAGTT TAGATGCAAG TTCAAGGAGC GAAAGGTGGA
4141 TGGGTAGGTT ATATAGGGAT ATAGCACAGA GATATATAGC AAAGAGATAC TTTTGAGCAA
4201 TGTTTGTGGA AGCGGTATTC GCAATATTTT AGTAGCTCGT TACAGTCCGG TGCGTTTTTG
4261 GTTTTTTGAA AGTGCGTCTT CAGAGCGCTT TTGGTTTTCA AAAGCGCTCT GAAGTTCCTA
4321 TACTTTCTAG CTAGAGAATA GGAACTTCGG AATAGGAACT TCAAAGCGTT TCCGAAAACG
4381 AGCGCTTCCG AAAATGCAAC GCGAGCTGCG CACATACAGC TCACTGTTCA CGTCGCACCT
4441 ATATCTGCGT GTTGCCTGTA TATATATATA CATGAGAAGA ACGGCATAGT GCGTGTTTAT
4501 GCTTAAATGC GTTATGGTGC ACTCTCAGTA CAATCTGCTC TGATGCCGCA TAGTTAAGCC
4561 AGCCCCGACA CCCGCCAACA CCCGCTGACG CGCCCTGACG GGCTTGTCTG CTCCCGGCAT
4621 CCGCTTACAG ACAAGCTGTG ACCGTCTCCG GGAGCTGCAT GTGTCAGAGG TTTTCACCGT
4681 CATCACCGAA ACGCGCGAGA CGAAAGGGCC TCGTGATACG CCTATTTTTA TAGGTTAATG
4741 TCATGATAAT AATGGTTTCT TAGACGTCAG GTGGCACTTT TCGGGGAAAT GTGCGCGGAA
4801 CCCCTATTTG TTTATTTTTC TAAATACATT CAAATATGTA TCCGCTCATG AGACAATAAC
4861 CCTGATAAAT GCTTCAATAA TATTGAAAAA GGAAGAGTAT GAGTATTCAA CATTTCCGTG
4921 TCGCCCTTAT TCCCTTTTTT GCGGCATTTT GCCTTCCTGT TTTTGCTCAC CCAGAAACGC
4981 TGGTGAAAGT AAAAGATGCT GAAGATCAGT TGGGTGCACG AGTGGGTTAC ATCGAACTGG
5041 ATCTCAACAG CGGTAAGATC CTTGAGAGTT TTCGCCCCGA AGAACGTTTT CCAATGATGA
5101 GCACTTTTAA AGTTCTGCTA TGTGGCGCGG TATTATCCCG TATTGACGCC GGGCAAGAGC
5161 AACTCGGTCG CCGCATACAC TATTCTCAGA ATGACTTGGT TGAGTACTCA CCAGTCACAG
5221 AAAAGCATCT TACGGATGGC ATGACAGTAA GAGAATTATG CAGTGCTGCC ATAACCATGA
5281 GTGATAACAC TGCGGCCAAC TTACTTCTGA CAACGATCGG AGGACCGAAG GAGCTAACCG
5341 CTTTTTTGCA CAACATGGGG GATCATGTAA CTCGCCTTGA TCGTTGGGAA CCGGAGCTGA
5401 ATGAAGCCAT ACCAAACGAC GAGCGTGACA CCACGATGCC TGTAGCAATG GCAACAACGT
5461 TGCGCAAACT ATTAACTGGC GAACTACTTA CTCTAGCTTC CCGGCAACAA TTAATAGACT
5521 GGATGGAGGC GGATAAAGTT GCAGGACCAC TTCTGCGCTC GGCCCTTCCG GCTGGCTGGT
5581 TTATTGCTGA TAAATCTGGA GCCGGTGAGC GTGGGTCTCG CGGTATCATT GCAGCACTGG
5641 GGCCAGATGG TAAGCCCTCC CGTATCGTAG TTATCTACAC GACGGGGAGT CAGGCAACTA
5701 TGGATGAACG AAATAGACAG ATCGCTGAGA TAGGTGCCTC ACTGATTAAG CATTGGTAAC
5761 TGTCAGACCA AGTTTACTCA TATATACTTT AGATTGATTT AAAACTTCAT TTTTAATTTA
5821 AAAGGATCTA GGTGAAGATC CTTTTTGATA ATCTCATGAC CAAAATCCCT TAACGTGAGT
5881 TTTCGTTCCA CTGAGCGTCA GACCCCGTAG AAAAGATCAA AGGATCTTCT TGAGATCCTT
5941 TTTTTCTGCG CGTAATCTGC TGCTTGCAAA CAAAAAAACC ACCGCTACCA GCGGTGGTTT
6001 GTTTGCCGGA TCAAGAGCTA CCAACTCTTT TTCCGAAGGT AACTGGCTTC AGCAGAGCGC
6061 AGATACCAAA TACTGTCCTT CTAGTGTAGC CGTAGTTAGG CCACCACTTC AAGAACTCTG
6121 TAGCACCGCC TACATACCTC GCTCTGCTAA TCCTGTTACC AGTGGCTGCT GCCAGTGGCG
6181 ATAAGTCGTG TCTTACCGGG TTGGACTCAA GACGATAGTT ACCGGATAAG GCGCAGCGGT
6241 CGGGCTGAAC GGGGGGTTCG TGCACACAGC CCAGCTTGGA GCGAACGACC TACACCGAAC
6301 TGAGATACCT ACAGCGTGAG CATTGAGAAA GCGCCACGCT TCCCGAAGGG AGAAAGGCGG
6361 ACAGGTATCC GGTAAGCGGC AGGGTCGGAA CAGGAGAGCG CACGAGGGAG CTTCCAGGGG
6421 GAAACGCCTG GTATCTTTAT AGTCCTGTCG GGTTTCGCCA CCTCTGACTT GAGCGTCGAT
6481 TTTTGTGATG CTCGTCAGGG GGGCGGAGCC TATGGAAAAA CGCCAGCAAC GCGGCCTTTT
6541 TACGGTTCCT GGCCTTTTGC TGGCCTTTTG CTCACATGTT CTTTCCTGCG TTATCCCCTG
6601 ATTCTGTGGA TAACCGTATT ACCGCCTTTG AGTGAGCTGA TACCGCTCGC CGCAGCCGAA

```
     6661 CGACCGAGCG CAGCGAGTCA GTGAGCGAGG AAGCGGAAGA GCGCCCAATA CGCAAACCGC
     6721 CTCTCCCCGC GCGTTGGCCG ATTCATTAAT C
//
LOCUS       YEP_VENUS_LEU2      8283 BP    DNA     CIRCULAR UNA 11-JAN-2011
DEFINITION
FEATURES             LOCATION/QUALIFIERS
     PROMOTER        6..719
                     /LABEL="TDH3 PROM"
     CDS             745..1461
                     /LABEL=VENUS
     TERMINATOR      1469..1691
                     /LABEL="ADH1 TERM"
     TERMINATOR      3000..3130
                     /LABEL="TDH3 TERM"
     CDS             COMPLEMENT(3339..4433)
                     /LABEL=LEU2
     REP_ORIGIN      4650..6044
                     /LABEL="2 MICRON"
     CDS             6431..7291
                     /LABEL=AMPR
     REP_ORIGIN      7446..8065
                     /LABEL=PBR322
ORIGIN
        1 GGATCCGTTA GAATCATTTT GAATAAAAAA CACGCTTTTT CAGTTCGAGT TTATCATTAT
       61 CAATACTGCC ATTTCAAAGA ATACGTAAAT AATTAATAGT AGTGATTTTC CTAACTTTAT
      121 TTAGTCAAAA AATTAGCCTT TTAATTCTGC TGTAACCCGT ACATGCCCAA AATAGGGGGC
      181 GGGTTACACA GAATATATAA CATCGTAGGT GTCTGGGTGA ACAGTTTATT CCTGGCATCC
      241 ACTAAATATA ATGGAGCCCG CTTTTTAAGC TGGCATCCAG AAAAAAAAAG AATCCCAGCA
      301 CCAAAATATT GTTTTCTTCA CCAACCATCA GTTCATAGGT CCATTCTCTT AGCGCAACTA
      361 CAGAGAACAG GGGCACAAAC AGGCAAAAAA CGGGCACAAC CTCAATGGAG TGATGCAACC
      421 TGCCTGGAGT AAATGATGAC ACAAGGCAAT TGACCCACGC ATGTATCTAT CTCATTTTCT
      481 TACACCTTCT ATTACCTTCT GCTCTCTCTG ATTTGGAAAA AGCTGAAAAA AAAGGTTGAA
      541 ACCAGTTCCC TGAAATTATT CCCCTACTTG ACTAATAAGT ATATAAAGAC GGTAGGTATT
      601 GATTGTAATT CTGTAAATCT ATTTCTTAAA CTTCTTAAAT TCTACTTTTA TAGTTAGTCT
      661 TTTTTTTAGT TTTAAAACAC CAAGAACTTA GTTTCGAATA AACACACATA AACAAACAAA
      721 GGTGACGGTG CTGGTTTAAT TAACATGTCT AAAGGTGAAG AATTATTCAC TGGTGTTGTC
      781 CCAATTTTGG TTGAATTAGA TGGTGATGTT AATGGTCACA AATTTTCTGT CTCCGGTGAA
      841 GGTGAAGGTG ATGCTACTTA CGGTAAATTG ACCTTAAAAT TGATTTGTAC TACTGGTAAA
      901 TTGCCAGTTC CATGGCCAAC CTTAGTCACT ACTTTAGGTT ATGGTTTGCA ATGTTTTGCT
      961 AGATACCCAG ATCATATGAA ACAACATGAC TTTTTCAAGT CTGCCATGCC AGAAGGTTAT
     1021 GTTCAAGAAA GAACTATTTT TTTCAAAGAT GACGGTAACT ACAAGACCAG AGCTGAAGTC
     1081 AAGTTTGAAG GTGATACCTT AGTTAATAGA ATCGAATTAA AAGGTATTGA TTTTAAAGAA
     1141 GATGGTAACA TTTTAGGTCA CAAATTGGAA TACAACTATA ACTCTCACAA TGTTTACATC
     1201 ACTGCTGACA AACAAAAGAA TGGTATCAAA GCTAACTTCA AAATTAGACA CAACATTGAA
     1261 GATGGTGGTG TTCAATTAGC TGACCATTAT CAACAAAATA CTCCAATTGG TGATGGTCCA
     1321 GTCTTGTTAC CAGACAACCA TTACTTATCC TATCAATCTG CCTTATCCAA AGATCCAAAC
     1381 GAAAAGAGAG ACCACATGGT CTTGTTAGAA TTTGTTACTG CTGCTGGTAT TACCCATGGT
     1441 ATGGATGAAT TGTACAAATA AGGCGCGCCA CTTCTAAATA AGCGAATTTC TTATGATTTA
     1501 TGATTTTTAT TATTAAATAA GTTATAAAAA AAATAAGTGT ATACAAATTT TAAAGTGACT
     1561 CTTAGGTTTT AAAACGAAAA TTCTTATTCT TGAGTAACTC TTTCCTGTAG GTCAGGTTGC
     1621 TTTCTCAGGT ATAGTATGAG GTCGCTCTTA TTGACCACAC CTCTACCGGC AGATCCGCTA
     1681 GGGATAACAG GGTAATATAG ATCTGTTTAG CTTGCCTCGT CCCCGCCGGG TCACCCGGCC
     1741 AGCGACATGG AGGCCCAGAA TACCCTCCTT GACAGTCTTG ACGTGCGCAG CTCAGGGGCA
     1801 TGATGTGACT GTCGCCCGTA CATTTAGCCC ATACATCCCC ATGTATAATC ATTTGCATCC
     1861 ATACATTTTG ATGGCCGCAC GGCGCGAAGC AAAAATTACG GCTCCTCGCT GCAGACCTGC
     1921 GAGCAGGGAA ACGCTCCCCT CACAGACGCG TTGAATTGTC CCCACGCCGC GCCCCTGTAG
     1981 AGAAATATAA AAGGTTAGGA TTTGCCACTG AGGTTCTTCT TTCATATACT TCCTTTTAAA
     2041 ATCTTGCTAG GATACAGTTC TCACATCACA TCCGAACATA AACAACCATG GGTAGGAGGG
     2101 CTTTTGTAGA AAGAAATACG AACGAAACGA AAATCAGCGT TGCCATCGCT TTGGACAAAG
     2161 CTCCCTTACC TGAAGAGTCG AATTTTATTG ATGAACTTAT AACTTCCAAG CATGCAAACC
     2221 AAAAGGGAGA ACAAGTAATC CAAGTAGACA CGGGAATTGG ATTCTTGGAT CACATGTATC
     2281 ATGCACTGGC TAAACATGCA GGCTGGAGCT TACGACTTTA CTCAAGAGGT GATTTAATCA
     2341 TCGATGATCA TCACACTGCA GAAGATACTG CTATTGCACT TGGTATTGCA TTCAAGCAGG
     2401 CTATGGGTAA CTTTGCCGGC GTTAAAAGAT TTGGACATGC TTATTGTCCA CTTGACGAAG
     2461 CTCTTTCTAG AAGCGTAGTT GACTTGTCGG GACGGCCCTA TGCTGTTATC GATTTGGGAT
     2521 TAAAGCGTGA AAAGGTTGGG GAATTGTCCT GTGAAATGAT CCCTCACTTA CTATATTCCT
     2581 TTTCGGTAGC AGCTGGAATT ACTTTGCATG TTACCTGCTT ATATGGTAGT AATGACCATC
     2641 ATCGTGCTGA AAGCGCTTTT AAATCTCTGG CTGTTGCCAT GCGCGCGGCT ACTAGTCTTA
     2701 CTGGAAGTTC TGAAGTCCCA AGCACGAAGG GAGTGTTGTA AAGAGTACTG ACAATAAAAA
     2761 GATTCTTGTT TTCAAGAACT TGTCATTTGT ATAGTTTTTT TATATTGTAG TTGTTCTATT
```

2821 TTAATCAAAT GTTAGCGTGA TTTATATTTT TTTTCGCCTC GACATCATCT GCCCAGATGC
2881 GAAGTTAAGT GCGCAGAAAG TAATATCATG CGTCAATCGT ATGTGAATGC TGGTCGCTAT
2941 ACTGCTGTCG ATTCGATACT AACGCCGCCA TCCAGTTTAA ACGAGCTCGA ATTCATCGAG
3001 TCGACTTGGT TGAACACGTT GCCAAGGCTT AAGTGAATTT ACTTTAAATC TTGCATTTAA
3061 ATAAATTTTC TTTTTATAGC TTTATGACTT AGTTTCAATT TATATACTAT TTTAATGACA
3121 TTTTCGATTC GGATCCCTGG CGTAATAGCG AAGAGGCCCG CACCGATCGC CCTTCCCAAC
3181 AGTTGCGCAG CCTGAATGGC GAATGGCGCC TGATGCGGTA TTTTCTCCTT ACGCATCTGT
3241 GCGGTATTTC ACACCGCATA GGGTAATAAC TGATATAATT AAATTGAAGC TCTAATTTGT
3301 GAGTTTAGTA TACATGCATT TACTTATAAT ACAGTTTTTT AAGCAAGGAT TTTCTTAACT
3361 TCTTCGGCGA CAGCATCACC GACTTCGGTG GTACTGTTGG AACCACCTAA ATCACCAGTT
3421 CTGATACCTG CATCCAAAAC CTTTTTAACT GCATCTTCAA TGGCCTTACC TTCTTCAGGC
3481 AAGTTCAATG ACAATTTCAA CATCATTGCA GCAGACAAGA TAGTGGCGAT AGGGTTGACC
3541 TTATTCTTTG GCAAATCTGG AGCAGAACCG TGGCATGGTT CGTACAAACC AAATGCGGTG
3601 TTCTTGTCTG GCAAAGAGGC CAAGGACGCA GATGGCAACA AACCCAAGGA ACCTGGGATA
3661 ACGGAGGCTT CATCGGAGAT GATATCACCA AACATGTTGC TGGTGATTAT AATACCATTT
3721 AGGTGGGTTG GGTTCTTAAC TAGGATCATG GCGGCAGAAT CAATCAATTG ATGTTGAACC
3781 TTCAATGTAG GGAATTCGTT CTTGATGGTT TCCTCCACAG TTTTTCTCCA TAATCTTGAA
3841 GAGGCCAAAA CATTAGCTTT ATCCAAGGAC CAAATAGGCA ATGGTGGCTC ATGTTGTAGG
3901 GCCATGAAAG CGGCCATTCT TGTGATTCTT TGCACTTCTG GAACGGTGTA TTGTTCACTA
3961 TCCCAAGCGA CACCATCACC ATCGTCTTCC TTTCTCTTAC CAAAGTAAAT ACCTCCCACT
4021 AATTCTCTGA CAACAACGAA GTCAGTACCT TTAGCAAATT GTGGCTTGAT TGGAGATAAG
4081 TCTAAAAGAG AGTCGGATGC AAAGTTACAT GGTCTTAAGT TGGCGTACAA TTGAAGTTCT
4141 TTACGGATTT TTAGTAAACC TTGTTCAGGT CTAACACTAC CGGTACCCCA TTTAGGACCA
4201 CCCACAGCAC CTAACAAAAC GGCATCAGCC TTCTTGGAGG CTTCCAGCGC CTCATCTGGA
4261 AGTGGAACAC CTGTAGCATC GATAGCAGCA CCACCAATTA AATGATTTTC GAAATCGAAC
4321 TTGACATTGG AACGAACATC AGAAATAGCT TTAAGAACCT TAATGGCTTC GGCTGTGATT
4381 TCTTGACCAA CGTGGTCACC TGGCAAAACG ACGATCTTCT TAGGGGCAGA CATGATTTAT
4441 CTTCGTTTCG GTTTTTGTTC TGTGCAGTTG GGTTAAGAAT ACTGGGCAAT TTCATGTTTC
4501 TTCAACACTA CATATGCGTA TATATACCAA TCTAAGTCTG TGCTCCTTCC TTCGTTCTTC
4561 CTTCTGTTCG GAGATTACCG AATCAAAAAA ATTTCAAAGA AACCGAAATC AAAAAAAAGA
4621 ATAAAAAAAA AATGATGAAT TGAAAAGCTC TTGTTACCCA TCATTGAATT TTGAACATCC
4681 GAACCTGGGA GTTTTCCCTG AAACAGATAG TATATTTGAA CCTGTATAAT AATATATAGT
4741 CTAGCGCTTT ACGGAAGACA ATGTATGTAT TTCGGTTCCT GGAGAAACTA TTGCATCTAT
4801 TGCATAGGTA ATCTTGCACG TCGCATCCCC GGTTCATTTT CTGCGTTTCC ATCTTGCACT
4861 TCAATAGCAT ATCTTTGTTA ACGAAGCATC TGTGCTTCAT TTTGTAGAAC AAAAATGCAA
4921 CGCGAGAGCG CTAATTTTTC AAACAAAGAA TCTGAGCTGC ATTTTTACAG AACAGAAATG
4981 CAACGCGAAA GCGCTATTTT ACCAACGAAG AATCTGTGCT TCATTTTTGT AAAACAAAAA
5041 TGCAACGCGA GAGCGCTAAT TTTTCAAACA AAGAATCTGA GCTGCATTTT TACAGAACAG
5101 AAATGCAACG CGAGAGCGCT ATTTTACCAA CAAAGAATCT ATACTTCTTT TTTGTTCTAC
5161 AAAAATGCAT CCCGAGAGCG CTATTTTTCT AACAAAGCAT CTTAGATTAC TTTTTTTCTC
5221 CTTTGTGCGC TCTATAATGC AGTCTCTTGA TAACTTTTTG CACTGTAGGT CCGTTAAGGT
5281 TAGAAGAAGG CTACTTTGGT GTCTATTTTC TCTTCCATAA AAAAAGCCTG ACTCCACTTC
5341 CCGCGTTTAC TGATTACTAG CGAAGCTGCG GGTGCATTTT TTCAAGATAA AGGCATCCCC
5401 GATTATATTC TATACCGATG TGGATTGCGC ATACTTTGTG AACAGAAAGT GATAGCGTTG
5461 ATGATTCTTC ATTGGTCAGA AAATTATGAA CGGTTTCTTC TATTTTGTCT CTATATACTA
5521 CGTATAGGAA ATGTTTACAT TTTCGTATTG TTTTCGATTC ACTCTATGAA TAGTTCTTAC
5581 TACAATTTTT TTGTCTAAAG AGTAATACTA GAGATAAACA TAAAAAATGT AGAGGTCGAG
5641 TTTAGATGCA AGTTCAAGGA GCGAAAGGTG GATGGGTAGG TTATATAGGG ATATAGCACA
5701 GAGATATATA GCAAAGAGAT ACTTTTGAGC AATGTTTGTG GAAGCGGTAT TCGCAATATT
5761 TTAGTAGCTC GTTACAGTCC GGTGCGTTTT TGGTTTTTTG AAAGTGCGTC TTCAGAGCGC
5821 TTTTGGTTTT CAAAAGCGCT CTGAAGTTCC TATACTTTCT AGCTAGAGAA TAGGAACTTC
5881 GGAATAGGAA CTTCAAAGCG TTTCCGAAAA CGAGCGCTTC CGAAAATGCA ACGCGAGCTG
5941 CGCACATACA GCTCACTGTT CACGTCGCAC CTATATCTGC GTGTTGCCTG TATATATATA
6001 TACATGAGAA GAACGGCATA GTGCGTGTTT ATGCTTAAAT GCGTTATGGT GCACTCTCAG
6061 TACAATCTGC TCTGATGCCG CATAGTTAAG CCAGCCCCGA CACCCGCCAA CACCCGCTGA
6121 CGCGCCCTGA CGGGCTTGTC TGCTCCCGGC ATCCGCTTAC AGACAAGCTG TGACCGTCTC
6181 CGGGAGCTGC ATGTGTCAGA GGTTTTCACC GTCATCACCG AAACGCGCGA GACGAAAGGG
6241 CCTCGTGATA CGCCTATTTT TATAGGTTAA TGTCATGATA ATAATGGTTT CTTAGACGTC
6301 AGGTGGCACT TTTCGGGGAA ATGTGCGCGG AACCCCTATT TGTTTATTTT TCTAAATACA
6361 TTCAAATATG TATCCGCTCA TGAGACAATA ACCCTGATAA ATGCTTCAAT AATATTGAAA
6421 AAGGAAGAGT ATGAGTATTC AACATTTCCG TGTCGCCCTT ATTCCCTTTT TTGCGGCATT
6481 TTGCCTTCCT GTTTTTGCTC ACCCAGAAAC GCTGGTGAAA GTAAAAGATG CTGAAGATCA
6541 GTTGGGTGCA CGAGTGGGTT ACATCGAACT GGATCTCAAC AGCGGTAAGA TCCTTGAGAG
6601 TTTTCGCCCC GAAGAACGTT TTCCAATGAT GAGCACTTTT AAAGTTCTGC TATGTGGCGC
6661 GGTATTATCC CGTATTGACG CCGGGCAAGA GCAACTCGGT CGCCGCATAC ACTATTCTCA
6721 GAATGACTTG GTTGAGTACT CACCAGTCAC AGAAAAGCAT CTTACGGATG GCATGACAGT
6781 AAGAGAATTA TGCAGTGCTG CCATAACCAT GAGTGATAAC ACTGCGGCCA ACTTACTTCT
6841 GACAACGATC GGAGGACCGA AGGAGCTAAC CGCTTTTTTG CACAACATGG GGGATCATGT
6901 AACTCGCCTT GATCGTTGGG AACCGGAGCT GAATGAAGCC ATACCAAACG ACGAGCGTGA
6961 CACCACGATG CCTGTAGCAA TGGCAACAAC GTTGCGCAAA CTATTAACTG GCGAACTACT

```
     7021 TACTCTAGCT TCCCGGCAAC AATTAATAGA CTGGATGGAG GCGGATAAAG TTGCAGGACC
     7081 ACTTCTGCGC TCGGCCCTTC CGGCTGGCTG GTTTATTGCT GATAAATCTG GAGCCGGTGA
     7141 GCGTGGGTCT CGCGGTATCA TTGCAGCACT GGGGCCAGAT GGTAAGCCCT CCCGTATCGT
     7201 AGTTATCTAC ACGACGGGGA GTCAGGCAAC TATGGATGAA CGAAATAGAC AGATCGCTGA
     7261 GATAGGTGCC TCACTGATTA AGCATTGGTA ACTGTCAGAC CAAGTTTACT CATATATACT
     7321 TTAGATTGAT TTAAAACTTC ATTTTTAATT TAAAAGGATC TAGGTGAAGA TCCTTTTTGA
     7381 TAATCTCATG ACCAAAATCC CTTAACGTGA GTTTTCGTTC CACTGAGCGT CAGACCCCGT
     7441 AGAAAAGATC AAAGGATCTT CTTGAGATCC TTTTTTTCTG CGCGTAATCT GCTGCTTGCA
     7501 AACAAAAAAA CCACCGCTAC CAGCGGTGGT TTGTTTGCCG GATCAAGAGC TACCAACTCT
     7561 TTTTCCGAAG GTAACTGGCT TCAGCAGAGC GCAGATACCA AATACTGTCC TTCTAGTGTA
     7621 GCCGTAGTTA GGCCACCACT TCAAGAACTC TGTAGCACCG CCTACATACC TCGCTCTGCT
     7681 AATCCTGTTA CCAGTGGCTG CTGCCAGTGG CGATAAGTCG TGTCTTACCG GGTTGGACTC
     7741 AAGACGATAG TTACCGGATA AGGCGCAGCG GTCGGGCTGA ACGGGGGGTT CGTGCACACA
     7801 GCCCAGCTTG GAGCGAACGA CCTACACCGA ACTGAGATAC CTACAGCGTG AGCATTGAGA
     7861 AAGCGCCACG CTTCCCGAAG GGAGAAAGGC GGACAGGTAT CCGGTAAGCG GCAGGGTCGG
     7921 AACAGGAGAG CGCACGAGGG AGCTTCCAGG GGGAAACGCC TGGTATCTTT ATAGTCCTGT
     7981 CGGGTTTCGC CACCTCTGAC TTGAGCGTCG ATTTTTGTGA TGCTCGTCAG GGGGGCGGAG
     8041 CCTATGGAAA AACGCCAGCA ACGCGGCCTT TTTACGGTTC CTGGCCTTTT GCTGGCCTTT
     8101 TGCTCACATG TTCTTTCCTG CGTTATCCCC TGATTCTGTG GATAACCGTA TTACCGCCTT
     8161 TGAGTGAGCT GATACCGCTC GCCGCAGCCG AACGACCGAG CGCAGCGAGT CAGTGAGCGA
     8221 GGAAGCGGAA GAGCGCCCAA TACGCAAACC GCCTCTCCCC GCGCGTTGGC CGATTCATTA
     8281 ATC
//
LOCUS       YEP_CFP_LEU2      8283 BP    DNA     CIRCULAR UNA 11-JAN-2011
DEFINITION
FEATURES             LOCATION/QUALIFIERS
     PROMOTER        6..719
                     /LABEL="TDH3 PROM"
     CDS             745..1461
                     /LABEL=CFP
     TERMINATOR      1469..1691
                     /LABEL="ADH1 TERM"
     TERMINATOR      3000..3130
                     /LABEL="TDH3 TERM"
     CDS             COMPLEMENT(3339..4433)
                     /LABEL=LEU2
     REP_ORIGIN      4650..6044
                     /LABEL="2 MICRON"
     CDS             6431..7291
                     /LABEL=AMPR
     REP_ORIGIN      7446..8065
                     /LABEL=PBR322
ORIGIN
        1 GGATCCGTTA GAATCATTTT GAATAAAAAA CACGCTTTTT CAGTTCGAGT TTATCATTAT
       61 CAATACTGCC ATTTCAAAGA ATACGTAAAT AATTAATAGT AGTGATTTTC CTAACTTTAT
      121 TTAGTCAAAA AATTAGCCTT TTAATTCTGC TGTAACCCGT ACATGCCCAA AATAGGGGGC
      181 GGGTTACACA GAATATATAA CATCGTAGGT GTCTGGGTGA ACAGTTTATT CCTGGCATCC
      241 ACTAAATATA ATGGAGCCCG CTTTTTAAGC TGGCATCCAG AAAAAAAAAG AATCCCAGCA
      301 CCAAAATATT GTTTTCTTCA CCAACCATCA GTTCATAGGT CCATTCTCTT AGCGCAACTA
      361 CAGAGAACAG GGGCACAAAC AGGCAAAAAA CGGGCACAAC CTCAATGGAG TGATGCAACC
      421 TGCCTGGAGT AAATGATGAC ACAAGGCAAT TGACCCACGC ATGTATCTAT CTCATTTTCT
      481 TACACCTTCT ATTACCTTCT GCTCTCTCTG ATTTGGAAAA AGCTGAAAAA AAAGGTTGAA
      541 ACCAGTTCCC TGAAATTATT CCCCTACTTG ACTAATAAGT ATATAAAGAC GGTAGGTATT
      601 GATTGTAATT CTGTAAATCT ATTTCTTAAA CTTCTTAAAT TCTACTTTTA TAGTTAGTCT
      661 TTTTTTTAGT TTTAAAACAC CAAGAACTTA GTTTCGAATA AACACACATA AACAAACAAA
      721 GGTGACGGTG CTGGTTTAAT TAACATGTCT AAAGGTGAAG AATTATTCAC TGGTGTTGTC
      781 CCAATTTTGG TTGAATTAGA TGGTGATGTT AATGGTCACA AATTTTCTGT CTCCGGTGAA
      841 GGTGAAGGTG ATGCTACTTA CGGTAAATTG ACCTTAAAAT TTATTTGTAC TACTGGTAAA
      901 TTGCCAGTTC CATGGCCAAC CTTAGTCACT ACTTTAACTT GGGGTGTTCA ATGTTTTTCT
      961 AGATACCCAG ATCATATGAA ACAACATGAC TTTTTCAAGT CTGCCATGCC AGAAGGTTAT
     1021 GTTCAAGAAA GAACTATTTT TTTCAAAGAT GACGGTAACT ACAAGACCAG AGCTGAAGTC
     1081 AAGTTTGAAG GTGATACCTT AGTTAATAGA ATCGAATTAA AAGGTATTGA TTTTAAAGAA
     1141 GATGGTAACA TTTTAGGTCA CAAATTGGAA TACATTTATA ACTCTCACAA TGTTTACATC
     1201 ACTGCTGACA AACAAAAGAA TGGTATCAAA GCTAACTTCA AAATTAGACA CAACATTGAA
     1261 GATGGTTCTG TTCAATTAGC TGACCATTAT CAACAAAATA CTCCAATTGG TGATGGTCCA
     1321 GTCTTGTTAC CAGACAACCA TTACTTATCC ACTCAATCTG CCTTATCCAA AGATCCAAAC
     1381 GAAAAGAGAG ACCACATGGT CTTGTTAGAA TTTGTTACTG CTGCTGGTAT TACCCATGGT
     1441 ATGGATGAAT TGTACAAATA AGGCGCGCCA CTTCTAAATA AGCGAATTTC TTATGATTTA
     1501 TGATTTTTAT TATTAAATAA GTTATAAAAA AAATAAGTGT ATACAAATTT TAAAGTGACT
     1561 CTTAGGTTTT AAAACGAAAA TTCTTATTCT TGAGTAACTC TTTCCTGTAG GTCAGGTTGC
```

1621 TTTCTCAGGT ATAGTATGAG GTCGCTCTTA TTGACCACAC CTCTACCGGC AGATCCGCTA
1681 GGGATAACAG GGTAATATAG ATCTGTTTAG CTTGCCTCGT CCCCGCCGGG TCACCCGGCC
1741 AGCGACATGG AGGCCCAGAA TACCCTCCTT GACAGTCTTG ACGTGCGCAG CTCAGGGGCA
1801 TGATGTGACT GTCGCCCGTA CATTTAGCCC ATACATCCCC ATGTATAATC ATTTGCATCC
1861 ATACATTTTG ATGGCCGCAC GGCGCGAAGC AAAAATTACG GCTCCTCGCT GCAGACCTGC
1921 GAGCAGGGAA ACGCTCCCCT CACAGACGCG TTGAATTGTC CCCACGCCGC GCCCCTGTAG
1981 AGAAATATAA AAGGTTAGGA TTTGCCACTG AGGTTCTTCT TTCATATACT TCCTTTTAAA
2041 ATCTTGCTAG GATACAGTTC TCACATCACA TCCGAACATA AACAACCATG GGTAGGAGGG
2101 CTTTTGTAGA AAGAAATACG AACGAAACGA AAATCAGCGT TGCCATCGCT TTGGACAAAG
2161 CTCCCTTACC TGAAGAGTCG AATTTTATTG ATGAACTTAT AACTTCCAAG CATGCAAACC
2221 AAAAGGGAGA ACAAGTAATC CAAGTAGACA CGGGAATTGG ATTCTTGGAT CACATGTATC
2281 ATGCACTGGC TAAACATGCA GGCTGGAGCT TACGACTTTA CTCAAGAGGT GATTTAATCA
2341 TCGATGATCA TCACACTGCA GAAGATACTG CTATTGCACT TGGTATTGCA TTCAAGCAGG
2401 CTATGGGTAA CTTTGCCGGC GTTAAAAGAT TTGGACATGC TTATTGTCCA CTTGACGAAG
2461 CTCTTTCTAG AAGCGTAGTT GACTTGTCGG GACGGCCCTA TGCTGTTATC GATTTGGGAT
2521 TAAAGCGTGA AAAGGTTGGG GAATTGTCCT GTGAAATGAT CCCTCACTTA CTATATTCCT
2581 TTTCGGTAGC AGCTGGAATT ACTTTGCATG TTACCTGCTT ATATGGTAGT AATGACCATC
2641 ATCGTGCTGA AAGCGCTTTT AAATCTCTGG CTGTTGCCAT GCGCGCGGCT ACTAGTCTTA
2701 CTGGAAGTTC TGAAGTCCCA AGCACGAAGG GAGTGTTGTA AAGAGTACTG ACAATAAAAA
2761 GATTCTTGTT TTCAAGAACT TGTCATTTGT ATAGTTTTTT TATATTGTAG TTGTTCTATT
2821 TTAATCAAAT GTTAGCGTGA TTTATATTTT TTTTCGCCTC GACATCATCT GCCCAGATGC
2881 GAAGTTAAGT GCGCAGAAAG TAATATCATG CGTCAATCGT ATGTGAATGC TGGTCGCTAT
2941 ACTGCTGTCG ATTCGATACT AACGCCGCCA TCCAGTTTAA ACGAGCTCGA ATTCATCGAG
3001 TCGACTTGGT TGAACACGTT GCCAAGGCTT AAGTGAATTT ACTTTAAATC TTGCATTTAA
3061 ATAAATTTTC TTTTTATAGC TTTATGACTT AGTTTCAATT TATATACTAT TTTAATGACA
3121 TTTTCGATTC GGATCCCTGG CGTAATAGCG AAGAGGCCCG CACCGATCGC CCTTCCCAAC
3181 AGTTGCGCAG CCTGAATGGC GAATGGCGCC TGATGCGGTA TTTTCTCCTT ACGCATCTGT
3241 GCGGTATTTC ACACCGCATA GGGTAATAAC TGATATAATT AAATTGAAGC TCTAATTTGT
3301 GAGTTTAGTA TACATGCATT TACTTATAAT ACAGTTTTTT AAGCAAGGAT TTTCTTAACT
3361 TCTTCGGCGA CAGCATCACC GACTTCGGTG GTACTGTTGG AACCACCTAA ATCACCAGTT
3421 CTGATACCTG CATCCAAAAC CTTTTTAACT GCATCTTCAA TGGCCTTACC TTCTTCAGGC
3481 AAGTTCAATG ACAATTTCAA CATCATTGCA GCAGACAAGA TAGTGGCGAT AGGGTTGACC
3541 TTATTCTTTG GCAAATCTGG AGCAGAACCG TGGCATGGTT CGTACAAACC AAATGCGGTG
3601 TTCTTGTCTG GCAAAGAGGC CAAGGACGCA GATGGCAACA AACCCAAGGA ACCTGGGATA
3661 ACGGAGGCTT CATCGGAGAT GATATCACCA AACATGTTGC TGGTGATTAT AATACCATTT
3721 AGGTGGGTTG GGTTCTTAAC TAGGATCATG GCGGCAGAAT CAATCAATTG ATGTTGAACC
3781 TTCAATGTAG GGAATTCGTT CTTGATGGTT TCCTCCACAG TTTTTCTCCA TAATCTTGAA
3841 GAGGCCAAAA CATTAGCTTT ATCCAAGGAC CAAATAGGCA ATGGTGGCTC ATGTTGTAGG
3901 GCCATGAAAG CGGCCATTCT TGTGATTCTT TGCACTTCTG GAACGGTGTA TTGTTCACTA
3961 TCCCAAGCGA CACCATCACC ATCGTCTTCC TTTCTCTTAC CAAAGTAAAT ACCTCCCACT
4021 AATTCTCTGA CAACAACGAA GTCAGTACCT TTAGCAAATT GTGGCTTGAT TGGAGATAAG
4081 TCTAAAAGAG AGTCGGATGC AAAGTTACAT GGTCTTAAGT TGGCGTACAA TTGAAGTTCT
4141 TTACGGATTT TTAGTAAACC TTGTTCAGGT CTAACACTAC CGGTACCCCA TTTAGGACCA
4201 CCCACAGCAC CTAACAAAAC GGCATCAGCC TTCTTGGAGG CTTCCAGCGC CTCATCTGGA
4261 AGTGGAACAC CTGTAGCATC GATAGCAGCA CCACCAATTA AATGATTTTC GAAATCGAAC
4321 TTGACATTGG AACGAACATC AGAAATAGCT TTAAGAACCT TAATGGCTTC GGCTGTGATT
4381 TCTTGACCAA CGTGGTCACC TGGCAAAACG ACGATCTTCT TAGGGGCAGA CATGATTTAT
4441 CTTCGTTTCG GTTTTTGTTC TGTGCAGTTG GGTTAAGAAT ACTGGGCAAT TTCATGTTTC
4501 TTCAACACTA CATATGCGTA TATATACCAA TCTAAGTCTG TGCTCCTTCC TTCGTTCTTC
4561 CTTCTGTTCG GAGATTACCG AATCAAAAAA ATTTCAAAGA AACCGAAATC AAAAAAAAGA
4621 ATAAAAAAAA AATGATGAAT TGAAAAGCTC TTGTTACCCA TCATTGAATT TTGAACATCC
4681 GAACCTGGGA GTTTTCCCTG AAACAGATAG TATATTTGAA CCTGTATAAT AATATATAGT
4741 CTAGCGCTTT ACGGAAGACA ATGTATGTAT TTCGGTTCCT GGAGAAACTA TTGCATCTAT
4801 TGCATAGGTA ATCTTGCACG TCGCATCCCC GGTTCATTTT CTGCGTTTCC ATCTTGCACT
4861 TCAATAGCAT ATCTTTGTTA ACGAAGCATC TGTGCTTCAT TTTGTAGAAC AAAAATGCAA
4921 CGCGAGAGCG CTAATTTTTC AAACAAAGAA TCTGAGCTGC ATTTTTACAG AACAGAAATG
4981 CAACGCGAAA GCGCTATTTT ACCAACGAAG AATCTGTGCT TCATTTTTGT AAAACAAAAA
5041 TGCAACGCGA GAGCGCTAAT TTTTCAAACA AAGAATCTGA GCTGCATTTT TACAGAACAG
5101 AAATGCAACG CGAGAGCGCT ATTTTACCAA CAAAGAATCT ATACTTCTTT TTTGTTCTAC
5161 AAAAATGCAT CCCGAGAGCG CTATTTTTCT AACAAAGCAT CTTAGATTAC TTTTTTTCTC
5221 CTTTGTGCGC TCTATAATGC AGTCTCTTGA TAACTTTTTG CACTGTAGGT CCGTTAAGGT
5281 TAGAAGAAGG CTACTTTGGT GTCTATTTTC TCTTCCATAA AAAAAGCCTG ACTCCACTTC
5341 CCGCGTTTAC TGATTACTAG CGAAGCTGCG GGTGCATTTT TTCAAGATAA AGGCATCCCC
5401 GATTATATTC TATACCGATG TGGATTGCGC ATACTTTGTG AACAGAAAGT GATAGCGTTG
5461 ATGATTCTTC ATTGGTCAGA AAATTATGAA CGGTTTCTTC TATTTTGTCT CTATATACTA
5521 CGTATAGGAA ATGTTTACAT TTTCGTATTG TTTTCGATTC ACTCTATGAA TAGTTCTTAC
5581 TACAATTTTT TTGTCTAAAG AGTAATACTA GAGATAAACA TAAAAAATGT AGAGGTCGAG
5641 TTTAGATGCA AGTTCAAGGA GCGAAAGGTG GATGGGTAGG TTATATAGGG ATATAGCACA
5701 GAGATATATA GCAAAGAGAT ACTTTTGAGC AATGTTTGTG GAAGCGGTAT TCGCAATATT
5761 TTAGTAGCTC GTTACAGTCC GGTGCGTTTT TGGTTTTTTG AAAGTGCGTC TTCAGAGCGC

```
     5821 TTTTGGTTTT CAAAAGCGCT CTGAAGTTCC TATACTTTCT AGCTAGAGAA TAGGAACTTC
     5881 GGAATAGGAA CTTCAAAGCG TTTCCGAAAA CGAGCGCTTC CGAAAATGCA ACGCGAGCTG
     5941 CGCACATACA GCTCACTGTT CACGTCGCAC CTATATCTGC GTGTTGCCTG TATATATATA
     6001 TACATGAGAA GAACGGCATA GTGCGTGTTT ATGCTTAAAT GCGTTATGGT GCACTCTCAG
     6061 TACAATCTGC TCTGATGCCG CATAGTTAAG CCAGCCCCGA CACCCGCCAA CACCCGCTGA
     6121 CGCGCCCTGA CGGGCTTGTC TGCTCCCGGC ATCCGCTTAC AGACAAGCTG TGACCGTCTC
     6181 CGGGAGCTGC ATGTGTCAGA GGTTTTCACC GTCATCACCG AAACGCGCGA GACGAAAGGG
     6241 CCTCGTGATA CGCCTATTTT TATAGGTTAA TGTCATGATA ATAATGGTTT CTTAGACGTC
     6301 AGGTGGCACT TTTCGGGGAA ATGTGCGCGG AACCCCTATT TGTTTATTTT TCTAAATACA
     6361 TTCAAATATG TATCCGCTCA TGAGACAATA ACCCTGATAA ATGCTTCAAT AATATTGAAA
     6421 AAGGAAGAGT ATGAGTATTC AACATTTCCG TGTCGCCCTT ATTCCCTTTT TTGCGGCATT
     6481 TTGCCTTCCT GTTTTTGCTC ACCCAGAAAC GCTGGTGAAA GTAAAAGATG CTGAAGATCA
     6541 GTTGGGTGCA CGAGTGGGTT ACATCGAACT GGATCTCAAC AGCGGTAAGA TCCTTGAGAG
     6601 TTTTCGCCCC GAAGAACGTT TTCCAATGAT GAGCACTTTT AAAGTTCTGC TATGTGGCGC
     6661 GGTATTATCC CGTATTGACG CCGGGCAAGA GCAACTCGGT CGCCGCATAC ACTATTCTCA
     6721 GAATGACTTG GTTGAGTACT CACCAGTCAC AGAAAAGCAT CTTACGGATG GCATGACAGT
     6781 AAGAGAATTA TGCAGTGCTG CCATAACCAT GAGTGATAAC ACTGCGGCCA ACTTACTTCT
     6841 GACAACGATC GGAGGACCGA AGGAGCTAAC CGCTTTTTTG CACAACATGG GGGATCATGT
     6901 AACTCGCCTT GATCGTTGGG AACCGGAGCT GAATGAAGCC ATACCAAACG ACGAGCGTGA
     6961 CACCACGATG CCTGTAGCAA TGGCAACAAC GTTGCGCAAA CTATTAACTG GCGAACTACT
     7021 TACTCTAGCT TCCCGGCAAC AATTAATAGA CTGGATGGAG GCGGATAAAG TTGCAGGACC
     7081 ACTTCTGCGC TCGGCCCTTC CGGCTGGCTG GTTTATTGCT GATAAATCTG GAGCCGGTGA
     7141 GCGTGGGTCT CGCGGTATCA TTGCAGCACT GGGGCCAGAT GGTAAGCCCT CCCGTATCGT
     7201 AGTTATCTAC ACGACGGGGA GTCAGGCAAC TATGGATGAA CGAAATAGAC AGATCGCTGA
     7261 GATAGGTGCC TCACTGATTA AGCATTGGTA ACTGTCAGAC CAAGTTTACT CATATATACT
     7321 TTAGATTGAT TTAAAACTTC ATTTTTAATT TAAAAGGATC TAGGTGAAGA TCCTTTTTGA
     7381 TAATCTCATG ACCAAAATCC CTTAACGTGA GTTTTCGTTC CACTGAGCGT CAGACCCCGT
     7441 AGAAAAGATC AAAGGATCTT CTTGAGATCC TTTTTTTCTG CGCGTAATCT GCTGCTTGCA
     7501 AACAAAAAAA CCACCGCTAC CAGCGGTGGT TTGTTTGCCG GATCAAGAGC TACCAACTCT
     7561 TTTTCCGAAG GTAACTGGCT TCAGCAGAGC GCAGATACCA AATACTGTCC TTCTAGTGTA
     7621 GCCGTAGTTA GGCCACCACT TCAAGAACTC TGTAGCACCG CCTACATACC TCGCTCTGCT
     7681 AATCCTGTTA CCAGTGGCTG CTGCCAGTGG CGATAAGTCG TGTCTTACCG GGTTGGACTC
     7741 AAGACGATAG TTACCGGATA AGGCGCAGCG GTCGGGCTGA ACGGGGGGTT CGTGCACACA
     7801 GCCCAGCTTG GAGCGAACGA CCTACACCGA ACTGAGATAC CTACAGCGTG AGCATTGAGA
     7861 AAGCGCCACG CTTCCCGAAG GGAGAAAGGC GGACAGGTAT CCGGTAAGCG GCAGGGTCGG
     7921 AACAGGAGAG CGCACGAGGG AGCTTCCAGG GGGAAACGCC TGGTATCTTT ATAGTCCTGT
     7981 CGGGTTTCGC CACCTCTGAC TTGAGCGTCG ATTTTTGTGA TGCTCGTCAG GGGGGCGGAG
     8041 CCTATGGAAA AACGCCAGCA ACGCGGCCTT TTTACGGTTC CTGGCCTTTT GCTGGCCTTT
     8101 TGCTCACATG TTCTTTCCTG CGTTATCCCC TGATTCTGTG GATAACCGTA TTACCGCCTT
     8161 TGAGTGAGCT GATACCGCTC GCCGCAGCCG AACGACCGAG CGCAGCGAGT CAGTGAGCGA
     8221 GGAAGCGGAA GAGCGCCCAA TACGCAAACC GCCTCTCCCC GCGCGTTGGC CGATTCATTA
     8281 ATC
//
LOCUS       YEP_SAPPHIRE_LEU2      8283 BP    DNA     CIRCULAR UNA 11-JAN-
2011
DEFINITION
FEATURES             LOCATION/QUALIFIERS
     PROMOTER        6..719
                     /LABEL="TDH3 PROM"
     CDS             745..1461
                     /LABEL=SAPPHIRE
     TERMINATOR      1469..1691
                     /LABEL="ADH1 TERM"
     TERMINATOR      3000..3130
                     /LABEL="TDH3 TERM"
     CDS             COMPLEMENT(3339..4433)
                     /LABEL=LEU2
     REP_ORIGIN      4650..6044
                     /LABEL="2 MICRON"
     CDS             6431..7291
                     /LABEL=AMPR
     REP_ORIGIN      7446..8065
                     /LABEL="PBR322 ORI"
                     /LABEL=PBR322
ORIGIN
        1 GGATCCGTTA GAATCATTTT GAATAAAAAA CACGCTTTTT CAGTTCGAGT TTATCATTAT
       61 CAATACTGCC ATTTCAAAGA ATACGTAAAT AATTAATAGT AGTGATTTTC CTAACTTTAT
      121 TTAGTCAAAA AATTAGCCTT TTAATTCTGC TGTAACCCGT ACATGCCCAA AATAGGGGGC
      181 GGGTTACACA GAATATATAA CATCGTAGGT GTCTGGGTGA ACAGTTTATT CCTGGCATCC
      241 ACTAAATATA ATGGAGCCCG CTTTTTAAGC TGGCATCCAG AAAAAAAAAG AATCCCAGCA
```

301 CCAAAATATT GTTTTCTTCA CCAACCATCA GTTCATAGGT CCATTCTCTT AGCGCAACTA
361 CAGAGAACAG GGGCACAAAC AGGCAAAAAA CGGGCACAAC CTCAATGGAG TGATGCAACC
421 TGCCTGGAGT AAATGATGAC ACAAGGCAAT TGACCCACGC ATGTATCTAT CTCATTTTCT
481 TACACCTTCT ATTACCTTCT GCTCTCTCTG ATTTGGAAAA AGCTGAAAAA AAAGGTTGAA
541 ACCAGTTCCC TGAAATTATT CCCCTACTTG ACTAATAAGT ATATAAAGAC GGTAGGTATT
601 GATTGTAATT CTGTAAATCT ATTTCTTAAA CTTCTTAAAT TCTACTTTTA TAGTTAGTCT
661 TTTTTTTAGT TTTAAAACAC CAAGAACTTA GTTTCGAATA AACACACATA AACAAACAAA
721 GGTGACGGTG CTGGTTTAAT TAACATGTCT AAAGGTGAAG AATTATTCAC TGGTGTTGTC
781 CCAATTTTGG TTGAATTAGA TGGTGATGTT AATGGTCACA AATTTTCTGT CTCCGGTGAA
841 GGTGAAGGTG ATGCTACTTA CGGTAAATTG ACCTTAAAAT TTATTTGTAC TACTGGTAAA
901 TTGCCAGTTC CATGGCCAAC CTTAGTCACT ACTTTTTCTT ATGGTGTTCA ATGTTTTGCT
961 AGATACCCAG ATCATATGAA ACAACATGAC TTTTTCAAGT CTGCCATGCC AGAAGGTTAT
1021 GTTCAAGAAA GAACTATTTT TTTCAAAGAT GACGGTAACT ACAAGACCAG AGCTGAAGTC
1081 AAGTTTGAAG GTGATACCTT AGTTAATAGA ATCGAATTAA AAGGTATTGA TTTTAAAGAA
1141 GATGGTAACA TTTTAGGTCA CAAATTGGAA TACAACTTTA ACTCTCACAA TGTTTACATC
1201 ATGGCTGACA AACAAAAGAA TGGTATCAAA GTTAACTTCA AAATTAGACA CAACATTGAA
1261 GATGGTTCTG TTCAATTAGC TGACCATTAT CAACAAAATA CTCCAATTGG TGATGGTCCA
1321 GTCTTGTTAC CAGACAACCA TTACTTATCC ATTCAATCTG CCTTATCCAA AGATCCAAAC
1381 GAAAAGAGAG ACCACATGGT CTTGTTAGAA TTTGTTACTG CTGCTGGTAT TACCCATGGT
1441 ATGGATGAAT TGTACAAATA AGGCGCGCCA CTTCTAAATA AGCGAATTTC TTATGATTTA
1501 TGATTTTTAT TATTAAATAA GTTATAAAAA AAATAAGTGT ATACAAATTT TAAAGTGACT
1561 CTTAGGTTTT AAAACGAAAA TTCTTATTCT TGAGTAACTC TTTCCTGTAG GTCAGGTTGC
1621 TTTCTCAGGT ATAGTATGAG GTCGCTCTTA TTGACCACAC CTCTACCGGC AGATCCGCTA
1681 GGGATAACAG GGTAATATAG ATCTGTTTAG CTTGCCTCGT CCCCGCCGGG TCACCCGGCC
1741 AGCGACATGG AGGCCCAGAA TACCCTCCTT GACAGTCTTG ACGTGCGCAG CTCAGGGGCA
1801 TGATGTGACT GTCGCCCGTA CATTTAGCCC ATACATCCCC ATGTATAATC ATTTGCATCC
1861 ATACATTTTG ATGGCCGCAC GGCGCGAAGC AAAAATTACG GCTCCTCGCT GCAGACCTGC
1921 GAGCAGGGAA ACGCTCCCCT CACAGACGCG TTGAATTGTC CCCACGCCGC GCCCCTGTAG
1981 AGAAATATAA AAGGTTAGGA TTTGCCACTG AGGTTCTTCT TTCATATACT TCCTTTTAAA
2041 ATCTTGCTAG GATACAGTTC TCACATCACA TCCGAACATA AACAACCATG GGTAGGAGGG
2101 CTTTTGTAGA AAGAAATACG AACGAAACGA AAATCAGCGT TGCCATCGCT TTGGACAAAG
2161 CTCCCTTACC TGAAGAGTCG AATTTTATTG ATGAACTTAT AACTTCCAAG CATGCAAACC
2221 AAAAGGGAGA ACAAGTAATC CAAGTAGACA CGGGAATTGG ATTCTTGGAT CACATGTATC
2281 ATGCACTGGC TAAACATGCA GGCTGGAGCT TACGACTTTA CTCAAGAGGT GATTTAATCA
2341 TCGATGATCA TCACACTGCA GAAGATACTG CTATTGCACT TGGTATTGCA TTCAAGCAGG
2401 CTATGGGTAA CTTTGCCGGC GTTAAAAGAT TTGGACATGC TTATTGTCCA CTTGACGAAG
2461 CTCTTTCTAG AAGCGTAGTT GACTTGTCGG GACGGCCCTA TGCTGTTATC GATTTGGGAT
2521 TAAAGCGTGA AAAGGTTGGG GAATTGTCCT GTGAAATGAT CCCTCACTTA CTATATTCCT
2581 TTTCGGTAGC AGCTGGAATT ACTTTGCATG TTACCTGCTT ATATGGTAGT AATGACCATC
2641 ATCGTGCTGA AAGCGCTTTT AAATCTCTGG CTGTTGCCAT GCGCGCGGCT ACTAGTCTTA
2701 CTGGAAGTTC TGAAGTCCCA AGCACGAAGG GAGTGTTGTA AAGAGTACTG ACAATAAAAA
2761 GATTCTTGTT TTCAAGAACT TGTCATTTGT ATAGTTTTTT TATATTGTAG TTGTTCTATT
2821 TTAATCAAAT GTTAGCGTGA TTTATATTTT TTTTCGCCTC GACATCATCT GCCCAGATGC
2881 GAAGTTAAGT GCGCAGAAAG TAATATCATG CGTCAATCGT ATGTGAATGC TGGTCGCTAT
2941 ACTGCTGTCG ATTCGATACT AACGCCGCCA TCCAGTTTAA ACGAGCTCGA ATTCATCGAG
3001 TCGACTTGGT TGAACACGTT GCCAAGGCTT AAGTGAATTT ACTTTAAATC TTGCATTTAA
3061 ATAAATTTTC TTTTTATAGC TTTATGACTT AGTTTCAATT TATATACTAT TTTAATGACA
3121 TTTTCGATTC GGATCCCTGG CGTAATAGCG AAGAGGCCCG CACCGATCGC CCTTCCCAAC
3181 AGTTGCGCAG CCTGAATGGC GAATGGCGCC TGATGCGGTA TTTTCTCCTT ACGCATCTGT
3241 GCGGTATTTC ACACCGCATA GGGTAATAAC TGATATAATT AAATTGAAGC TCTAATTTGT
3301 GAGTTTAGTA TACATGCATT TACTTATAAT ACAGTTTTTT AAGCAAGGAT TTTCTTAACT
3361 TCTTCGGCGA CAGCATCACC GACTTCGGTG GTACTGTTGG AACCACCTAA ATCACCAGTT
3421 CTGATACCTG CATCCAAAAC CTTTTTAACT GCATCTTCAA TGGCCTTACC TTCTTCAGGC
3481 AAGTTCAATG ACAATTTCAA CATCATTGCA GCAGACAAGA TAGTGGCGAT AGGGTTGACC
3541 TTATTCTTTG GCAAATCTGG AGCAGAACCG TGGCATGGTT CGTACAAACC AAATGCGGTG
3601 TTCTTGTCTG GCAAAGAGGC CAAGGACGCA GATGGCAACA AACCCAAGGA ACCTGGGATA
3661 ACGGAGGCTT CATCGGAGAT GATATCACCA AACATGTTGC TGGTGATTAT AATACCATTT
3721 AGGTGGGTTG GGTTCTTAAC TAGGATCATG GCGGCAGAAT CAATCAATTG ATGTTGAACC
3781 TTCAATGTAG GGAATTCGTT CTTGATGGTT TCCTCCACAG TTTTTCTCCA TAATCTTGAA
3841 GAGGCCAAAA CATTAGCTTT ATCCAAGGAC CAAATAGGCA ATGGTGGCTC ATGTTGTAGG
3901 GCCATGAAAG CGGCCATTCT TGTGATTCTT TGCACTTCTG GAACGGTGTA TTGTTCACTA
3961 TCCCAAGCGA CACCATCACC ATCGTCTTCC TTTCTCTTAC CAAAGTAAAT ACCTCCCACT
4021 AATTCTCTGA CAACAACGAA GTCAGTACCT TTAGCAAATT GTGGCTTGAT TGGAGATAAG
4081 TCTAAAAGAG AGTCGGATGC AAAGTTACAT GGTCTTAAGT TGGCGTACAA TTGAAGTTCT
4141 TTACGGATTT TTAGTAAACC TTGTTCAGGT CTAACACTAC CGGTACCCCA TTTAGGACCA
4201 CCCACAGCAC CTAACAAAAC GGCATCAGCC TTCTTGGAGG CTTCCAGCGC CTCATCTGGA
4261 AGTGGAACAC CTGTAGCATC GATAGCAGCA CCACCAATTA AATGATTTTC GAAATCGAAC
4321 TTGACATTGG AACGAACATC AGAAATAGCT TTAAGAACCT TAATGGCTTC GGCTGTGATT
4381 TCTTGACCAA CGTGGTCACC TGGCAAAACG ACGATCTTCT TAGGGGCAGA CATGATTTAT
4441 CTTCGTTTCG GTTTTTGTTC TGTGCAGTTG GGTTAAGAAT ACTGGGCAAT TTCATGTTTC

4501 TTCAACACTA CATATGCGTA TATATACCAA TCTAAGTCTG TGCTCCTTCC TTCGTTCTTC
4561 CTTCTGTTCG GAGATTACCG AATCAAAAAA ATTTCAAAGA AACCGAAATC AAAAAAAAGA
4621 ATAAAAAAAA AATGATGAAT TGAAAAGCTC TTGTTACCCA TCATTGAATT TTGAACATCC
4681 GAACCTGGGA GTTTTCCCTG AAACAGATAG TATATTTGAA CCTGTATAAT AATATATAGT
4741 CTAGCGCTTT ACGGAAGACA ATGTATGTAT TTCGGTTCCT GGAGAAACTA TTGCATCTAT
4801 TGCATAGGTA ATCTTGCACG TCGCATCCCC GGTTCATTTT CTGCGTTTCC ATCTTGCACT
4861 TCAATAGCAT ATCTTTGTTA ACGAAGCATC TGTGCTTCAT TTTGTAGAAC AAAAATGCAA
4921 CGCGAGAGCG CTAATTTTTC AAACAAAGAA TCTGAGCTGC ATTTTTACAG AACAGAAATG
4981 CAACGCGAAA GCGCTATTTT ACCAACGAAG AATCTGTGCT TCATTTTTGT AAAACAAAAA
5041 TGCAACGCGA GAGCGCTAAT TTTTCAAACA AAGAATCTGA GCTGCATTTT TACAGAACAG
5101 AAATGCAACG CGAGAGCGCT ATTTTACCAA CAAAGAATCT ATACTTCTTT TTTGTTCTAC
5161 AAAAATGCAT CCCGAGAGCG CTATTTTTCT AACAAAGCAT CTTAGATTAC TTTTTTTCTC
5221 CTTTGTGCGC TCTATAATGC AGTCTCTTGA TAACTTTTTG CACTGTAGGT CCGTTAAGGT
5281 TAGAAGAAGG CTACTTTGGT GTCTATTTTC TCTTCCATAA AAAAAGCCTG ACTCCACTTC
5341 CCGCGTTTAC TGATTACTAG CGAAGCTGCG GGTGCATTTT TTCAAGATAA AGGCATCCCC
5401 GATTATATTC TATACCGATG TGGATTGCGC ATACTTTGTG AACAGAAAGT GATAGCGTTG
5461 ATGATTCTTC ATTGGTCAGA AAATTATGAA CGGTTTCTTC TATTTTGTCT CTATATACTA
5521 CGTATAGGAA ATGTTTACAT TTTCGTATTG TTTTCGATTC ACTCTATGAA TAGTTCTTAC
5581 TACAATTTTT TTGTCTAAAG AGTAATACTA GAGATAAACA TAAAAAATGT AGAGGTCGAG
5641 TTTAGATGCA AGTTCAAGGA GCGAAAGGTG GATGGGTAGG TTATATAGGG ATATAGCACA
5701 GAGATATATA GCAAAGAGAT ACTTTTGAGC AATGTTTGTG GAAGCGGTAT TCGCAATATT
5761 TTAGTAGCTC GTTACAGTCC GGTGCGTTTT TGGTTTTTTG AAAGTGCGTC TTCAGAGCGC
5821 TTTTGGTTTT CAAAAGCGCT CTGAAGTTCC TATACTTTCT AGCTAGAGAA TAGGAACTTC
5881 GGAATAGGAA CTTCAAAGCG TTTCCGAAAA CGAGCGCTTC CGAAAATGCA ACGCGAGCTG
5941 CGCACATACA GCTCACTGTT CACGTCGCAC CTATATCTGC GTGTTGCCTG TATATATATA
6001 TACATGAGAA GAACGGCATA GTGCGTGTTT ATGCTTAAAT GCGTTATGGT GCACTCTCAG
6061 TACAATCTGC TCTGATGCCG CATAGTTAAG CCAGCCCCGA CACCCGCCAA CACCCGCTGA
6121 CGCGCCCTGA CGGGCTTGTC TGCTCCCGGC ATCCGCTTAC AGACAAGCTG TGACCGTCTC
6181 CGGGAGCTGC ATGTGTCAGA GGTTTTCACC GTCATCACCG AAACGCGCGA GACGAAAGGG
6241 CCTCGTGATA CGCCTATTTT TATAGGTTAA TGTCATGATA ATAATGGTTT CTTAGACGTC
6301 AGGTGGCACT TTTCGGGGAA ATGTGCGCGG AACCCCTATT TGTTTATTTT TCTAAATACA
6361 TTCAAATATG TATCCGCTCA TGAGACAATA ACCCTGATAA ATGCTTCAAT AATATTGAAA
6421 AAGGAAGAGT ATGAGTATTC AACATTTCCG TGTCGCCCTT ATTCCCTTTT TTGCGGCATT
6481 TTGCCTTCCT GTTTTTGCTC ACCCAGAAAC GCTGGTGAAA GTAAAAGATG CTGAAGATCA
6541 GTTGGGTGCA CGAGTGGGTT ACATCGAACT GGATCTCAAC AGCGGTAAGA TCCTTGAGAG
6601 TTTTCGCCCC GAAGAACGTT TTCCAATGAT GAGCACTTTT AAAGTTCTGC TATGTGGCGC
6661 GGTATTATCC CGTATTGACG CCGGGCAAGA GCAACTCGGT CGCCGCATAC ACTATTCTCA
6721 GAATGACTTG GTTGAGTACT CACCAGTCAC AGAAAAGCAT CTTACGGATG GCATGACAGT
6781 AAGAGAATTA TGCAGTGCTG CCATAACCAT GAGTGATAAC ACTGCGGCCA ACTTACTTCT
6841 GACAACGATC GGAGGACCGA AGGAGCTAAC CGCTTTTTTG CACAACATGG GGGATCATGT
6901 AACTCGCCTT GATCGTTGGG AACCGGAGCT GAATGAAGCC ATACCAAACG ACGAGCGTGA
6961 CACCACGATG CCTGTAGCAA TGGCAACAAC GTTGCGCAAA CTATTAACTG GCGAACTACT
7021 TACTCTAGCT TCCCGGCAAC AATTAATAGA CTGGATGGAG GCGGATAAAG TTGCAGGACC
7081 ACTTCTGCGC TCGGCCCTTC CGGCTGGCTG GTTTATTGCT GATAAATCTG GAGCCGGTGA
7141 GCGTGGGTCT CGCGGTATCA TTGCAGCACT GGGGCCAGAT GGTAAGCCCT CCCGTATCGT
7201 AGTTATCTAC ACGACGGGGA GTCAGGCAAC TATGGATGAA CGAAATAGAC AGATCGCTGA
7261 GATAGGTGCC TCACTGATTA AGCATTGGTA ACTGTCAGAC CAAGTTTACT CATATATACT
7321 TTAGATTGAT TTAAAACTTC ATTTTTAATT TAAAAGGATC TAGGTGAAGA TCCTTTTTGA
7381 TAATCTCATG ACCAAAATCC CTTAACGTGA GTTTTCGTTC CACTGAGCGT CAGACCCCGT
7441 AGAAAAGATC AAAGGATCTT CTTGAGATCC TTTTTTTCTG CGCGTAATCT GCTGCTTGCA
7501 AACAAAAAAA CCACCGCTAC CAGCGGTGGT TTGTTTGCCG GATCAAGAGC TACCAACTCT
7561 TTTTCCGAAG GTAACTGGCT TCAGCAGAGC GCAGATACCA AATACTGTCC TTCTAGTGTA
7621 GCCGTAGTTA GGCCACCACT TCAAGAACTC TGTAGCACCG CCTACATACC TCGCTCTGCT
7681 AATCCTGTTA CCAGTGGCTG CTGCCAGTGG CGATAAGTCG TGTCTTACCG GGTTGGACTC
7741 AAGACGATAG TTACCGGATA AGGCGCAGCG GTCGGGCTGA ACGGGGGGTT CGTGCACACA
7801 GCCCAGCTTG GAGCGAACGA CCTACACCGA ACTGAGATAC CTACAGCGTG AGCATTGAGA
7861 AAGCGCCACG CTTCCCGAAG GGAGAAAGGC GGACAGGTAT CCGGTAAGCG GCAGGGTCGG
7921 AACAGGAGAG CGCACGAGGG AGCTTCCAGG GGGAAACGCC TGGTATCTTT ATAGTCCTGT
7981 CGGGTTTCGC CACCTCTGAC TTGAGCGTCG ATTTTTGTGA TGCTCGTCAG GGGGGCGGAG
8041 CCTATGGAAA AACGCCAGCA ACGCGGCCTT TTTACGGTTC CTGGCCTTTT GCTGGCCTTT
8101 TGCTCACATG TTCTTTCCTG CGTTATCCCC TGATTCTGTG GATAACCGTA TTACCGCCTT
8161 TGAGTGAGCT GATACCGCTC GCCGCAGCCG AACGACCGAG CGCAGCGAGT CAGTGAGCGA
8221 GGAAGCGGAA GAGCGCCCAA TACGCAAACC GCCTCTCCCC GCGCGTTGGC CGATTCATTA
8281 ATC

//

Supplementary Spreadsheet S1. *Nyfedge Maybridge Hits.*

Supplementary Spreadsheet S2. *Number Chemblntd compounds with similarities to Nyfedge's antiplasmodial hits at different thresholds.*

Supplementary Spreadsheet S3. *Overlap between compounds identified as hits in Nyfedge high-throughput screens.*

Supplementary references

1. Gari E., Piedrafita L., Aldea M., Herrero E. 1997 A set of vectors with a tetracycline-regulatable promoter system for modulated gene expression in Saccharomyces cerevisiae. *Yeast* **13**(9), 837-848. (doi:10.1002/(SICI)1097-0061(199707)13:9<837::AID-YEA145>3.0.CO;2-T [pii] 10.1002/(SICI)1097-0061(199707)13:9<837::AID-YEA145>3.0.CO;2-T).
2. Bilsland E., Pir P., Gutteridge A., Johns A., King R.D., Oliver S.G. 2011 Functional expression of parasite drug targets and their human orthologs in yeast. *Plos Neglected Tropical Diseases* **5**(10), e1320./ (doi:10.1371/journal.pntd.0001320/PNTD-D-11-00499 [pii]).
3. Sheff M.A., Thorn K.S. 2004 Optimized cassettes for fluorescent protein tagging in Saccharomyces cerevisiae. *Yeast* **21**(8), 661-670. (doi:10.1002/yea.1130).
4. Christianson T.W., Sikorski R.S., Dante M., Shero J.H., Hieter P. 1992 Multifunctional yeast high-copy-number shuttle vectors. *Gene* **110**(1), 119-122. (doi:0378-1119(92)90454-W [pii]).
5. Keppler-Ross S., Noffz C., Dean N. 2008 A new purple fluorescent color marker for genetic studies in Saccharomyces cerevisiae and Candida albicans. *Genetics* **179**(1), 705-710. (doi:179/1/705 [pii]/10.1534/genetics.108.087080).
6. Brachmann C.B., Davies A., Cost G.J., Caputo E., Li J., Hieter P., Boeke J.D. 1998 Designer deletion strains derived from Saccharomyces cerevisiae S288C: a useful set of strains and plasmids for PCR-mediated gene disruption and other applications. *Yeast* **14**(2), 115-132. (doi:10.1002/(SICI)1097-0061(19980130)14:2<115::AID-YEA204>3.0.CO;2-2 [pii]/ 10.1002/(SICI)1097-0061(19980130)14:2<115::AID-YEA204>3.0.CO;2-2).
